# A Transcriptome Meta-Analysis Proposes Novel Biological Roles for the Antifungal Protein AnAFP in *Aspergillus niger*

**DOI:** 10.1371/journal.pone.0165755

**Published:** 2016-11-11

**Authors:** Norman Paege, Sascha Jung, Paul Schäpe, Dirk Müller-Hagen, Jean-Paul Ouedraogo, Caroline Heiderich, Johanna Jedamzick, Benjamin M. Nitsche, Cees A. van den Hondel, Arthur F. Ram, Vera Meyer

**Affiliations:** 1 Institute of Biotechnology, Department Applied and Molecular Microbiology, Berlin University of Technology, Berlin, Germany; 2 Leiden University, Institute of Biology Leiden, Department Molecular Microbiology and Biotechnology, Leiden, The Netherlands; 3 HiTexaCoat, Gouda, The Netherlands; Woosuk University, REPUBLIC OF KOREA

## Abstract

Understanding the genetic, molecular and evolutionary basis of cysteine-stabilized antifungal proteins (AFPs) from fungi is important for understanding whether their function is mainly defensive or associated with fungal growth and development. In the current study, a transcriptome meta-analysis of the *Aspergillus niger* γ-core protein AnAFP was performed to explore co-expressed genes and pathways, based on independent expression profiling microarrays covering 155 distinct cultivation conditions. This analysis uncovered that *anafp* displays a highly coordinated temporal and spatial transcriptional profile which is concomitant with key nutritional and developmental processes. Its expression profile coincides with early starvation response and parallels with genes involved in nutrient mobilization and autophagy. Using fluorescence- and luciferase reporter strains we demonstrated that the *anafp* promoter is active in highly vacuolated compartments and foraging hyphal cells during carbon starvation with CreA and FlbA, but not BrlA, as most likely regulators of *anafp*. A co-expression network analysis supported by luciferase-based reporter assays uncovered that *anafp* expression is embedded in several cellular processes including allorecognition, osmotic and oxidative stress survival, development, secondary metabolism and autophagy, and predicted StuA and VelC as additional regulators. The transcriptomic resources available for *A*. *niger* provide unparalleled resources to investigate the function of proteins. Our work illustrates how transcriptomic meta-analyses can lead to hypotheses regarding protein function and predict a role for AnAFP during slow growth, allorecognition, asexual development and nutrient recycling of *A*. *niger* and propose that it interacts with the autophagic machinery to enable these processes.

## Introduction

Virtually all prokaryotic and eukaryotic organisms rely on antimicrobial peptides to rapidly ward off microbial competitors, predators and invaders. Although produced in phylogenetically distant kingdoms, a unifying structural signature present in all cysteine-stabilized antimicrobial peptides from bacterial, fungal, plant and (in)vertebrate origin has been discovered: the γ-core motif [1]. It is a conserved three-dimensional structural form that arises from the sequence motif GXC(X_3–9_)C and is characterized by a twisted two-stranded antiparallel β-sheet with an interposed short turn region. Intriguingly, the γ-core motif is not only present in antimicrobial peptides but also in other host defense polypeptides, including human kinocidins (a group of microbicidal cytokines and chemokines), arachnid neurotoxins, reptile venoms, invertebrate toxins and even in sweetener polypeptides such as brazzein [2, 3]. Virtually all peptides that share the γ-core motif typically exert membrane-targeting and/or membrane-modulating mechanisms. Host specificity and differences in microbicidal activity is determined by specific protein features such as the presence of certain amino-acid side chains or other structural motifs. Thus, it is proposed that cysteine-stabilized antimicrobial peptides and related host defense effector polypeptides are descended from early prokaryotic and eukaryotic predecessor molecules with the γ-core as an archetypal membrane-interaction motif. The function of these ancient molecules was likely to defend one microorganism from another [2]. Positive selective pressure during phylogenetic diversification ensured conservation of the γ-core motif but optimized these molecules for new, diverse and host-specific roles. For example, some antimicrobial plant defensins exert functions during root development [4]. Elsewhere, leech antimicrobial peptides participate in neuronal regeneration [5]. Likewise, antimicrobial human defensins also participate in diverse cellular processes such as mast cell degranulation [6] or stimulation of endothelial cells during wound healing [7].

Almost nothing is known about the biological function of cysteine-stabilized antimicrobial peptides from filamentous fungi. These proteins are of particular interest for applied research as the two most intensively studied—AFP from *Aspergillus giganteus* and PAF from *Penicillium chrysogenum*–exclusively inhibit the growth of other fungi by disturbing their cell-membrane integrity (for reviews see [8, 9]. Moreover, as these proteins do not affect growth or viability of bacterial, plant or mammalian systems, AFP and PAF bear great potential for use in medical or agricultural applications. These molecules could be applied to combat human and plant pathogenic fungi, which pose a persistent and growing cause of human disease and mortality, and also a threat to international food security through crop destruction [10, 11].

The recent sequencing of dozens of filamentous fungal genomes, the availability of hundreds of fungal transcriptomic and proteomic datasets and the advent of increasingly sophisticated bioinformatics and genetic manipulation tools [12], make it now for the first time feasible to study the phylogenetic relationship of antifungal peptides from fungal origin, to uncover the underlying gene regulatory network ensuring their expression and to identify co-expressed genes and proteins under different physiological conditions. In turn, this data will significantly advance our understanding of the biological role and evolution of antifungal proteins.

The *A*. *niger* isolate CBS 513.88 produces an antifungal protein (AnAFP), and we selected this isolate as a system to assess the biological role of fungal AFPs for several reasons. Firstly, while the antifungal properties of AnAFP to yeast and filamentous fungi have been previously reported and sequence composition confirmed[13], it has not been determined whether this inhibitory effect is mediated due to membrane permeabilisation. Consequently, AnAFP is an excellent candidate to interrogate the common ancestor hypothesis put forward by Yeaman and Yount [2], *i*.*e*. is AnAFP antifungal efficacy due to a conserved γ-core motif which interacts with fungal membranes. Secondly, genome sequence data of 20 *Aspergillus* species are freely available at the *Aspergillus* Genome Database (AspGD; http://www.aspgd.org/) [14, 15]. This resource is an outstanding toolbox for comparative genomics, allowing us to study phylogenetic relationships and evolution of AnAFP orthologs in the genus *Aspergillus*. Thirdly, the genome of the *A*. *niger* strain CBS 513.88 belongs to the best annotated *Aspergillus* genomes [16] and hundreds of transcriptomics and proteomics data have been published for this strain. Genome-wide expression profiles of strain CBS 513.88 were published for different cultivation conditions, developmental stages and environmental stress conditions (for reviews see [17, 18], thus constituting an invaluable treasure chest which can be systematically mined for expression of the *anafp* gene. Finally, we have developed various bioinformatics pipelines for the analysis of genomic and transcriptomic data of *A*. *niger* CBS 513.88 [19, 20]. Altogether, these circumstances form a well-grounded framework allowing us to study the time- and spatially-dependent expression profile of *anafp* and to determine the biological role for *A*. *niger* at a systems level.

In the current study, a transcriptome meta-analysis of the *Aspergillus niger* γ-core protein AnAFP was performed. This analysis uncovered the highly coordinated temporal and spacial transcriptional profile of *anafp* and led to the identification of putative regulatory elements. Its concomitant expression with key nutritional and developmental processes was corroborated *in vivo*. Our work illustrates how transcriptomic meta-analyses can lead to hypotheses regarding protein function and predict a role for AnAFP during slow growth, allorecognition, asexual development and nutrient recycling of *A*. *niger* and propose that it interacts with the autophagic machinery to enable these processes.

## Results

### AnAFP causes membrane permeabilization in sensitive fungi

Table 1 demonstrates that AnAFP has comparable levels of antifungal efficacy when compared to AFP produced by *A*. *giganteus*. AnAFP efficiently inhibits the growth of multiple filamentous fungi, with minimal inhibitory concentrations (MICs) ranging from 1 μg/ml to 50 μg/ml. Different sensitivities of fungi towards AFPs are in good agreement with our observations for AFP, and can mechanistically be explained by the damage-response framework [21]. Interestingly, both producing strains are only moderate-sensitive towards their own antifungal protein but very sensitive towards the other protein (Table 1). This suggests that *A*. *niger* and *A*. *giganteus* possess innate sensing or defense systems which enable them to discriminate between AFPs from self or non-self origin.

**Table 1:** Minimal inhibitory concentrations (MICs) of AnAFP and AFP on selected fungi.

Strain	MIC AnAFP (μg/ml)	MIC AFP (μg/ml)
*Aspergillus niger* N402	> 50*	1
*Aspergillus giganteus* MDH 18894	30	> 400^§^
*Aspergillus oryzae* ATCC 11488	NE^#^	NE
*Aspergillus nidulans* DSM 969	> 50	200
*Fusarium oxysporum* IfGB 39/1201	1	1
*Fusarium poae* DSM 62376	1	15
*Fusarium sporotrichoides* DSM 62425	2	1
*Penicillium chrysogenum* ATCC 10002	> 50	NE

*The maximum concentration of AnAFP which we could use for the MIC assay was 50 μg/ml as only limited amounts of AnAFP were obtainable from *A*. *niger* cultures.

^§^No complete growth inhibition was detected at concentrations of 400μg/ml.

^#^NE, no effect. Values are averages of triplicate experiments.

**Fig 1:**
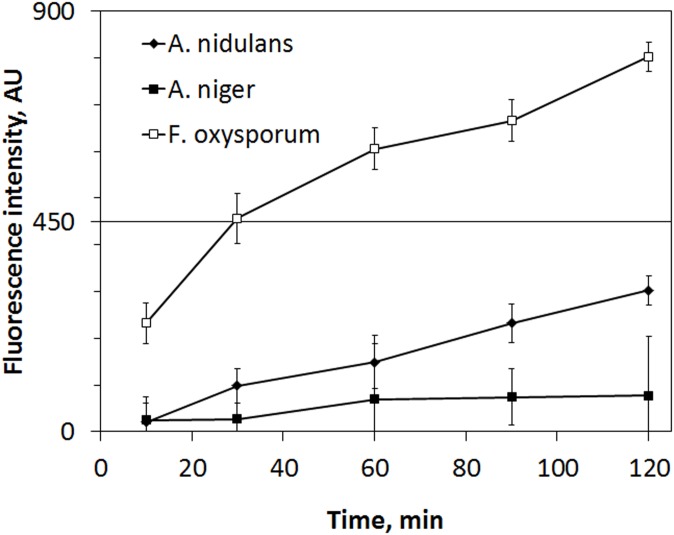
Membrane permeabilization induced by AnAFP in *A*. *niger*. The capability of AnAFP (100 μg/ml) to permeabilize the membranes of viable cells of *A*. *niger*, *A*. *nidulans* and *F*. *oxysporum* was monitored for 2 h of incubation. The fluorescence of the SYTOX Green dye was used as a measure of fungal cells with compromised membranes. Cells with intact membranes do not show a fluorescence signal. Data are averages of triplicate experiments.

To determine whether AnAFP is able to permabilize fungal membranes, a well-established assay based on the uptake of the fluorogenic DNA-binding dye SYTOX green was implemented. This dye can enter compromised membranes and has been used to demonstrate that AFP-mediated growth inhibition is due to permeabilization of fungal plasma membranes [22]. As depicted in Fig 1A, 100 μg/ml AnAFP indeed readily permeabilizes the plasma membrane of the AnAFP-sensitive strain *F*. *oxysporum*. In contrast, the plasma membranes of the strains *A*. *niger* (MIC > 50 μg/ml) remained intact when treated with 100 μg/ml AnAFP, demonstrating that this strain is much less susceptible towards AnAFP. Surprisingly, *A*. *nidulans* is more susceptible to membrane permeabilization than *A*. *niger*, although this strain shows a similar MIC-value. These findings might point to a different damage-response framework in *A*. *nidulans* compared to *A*. *niger*.

### *anafp* expression is carbon-dependent and under CreA control

**Fig 2:**
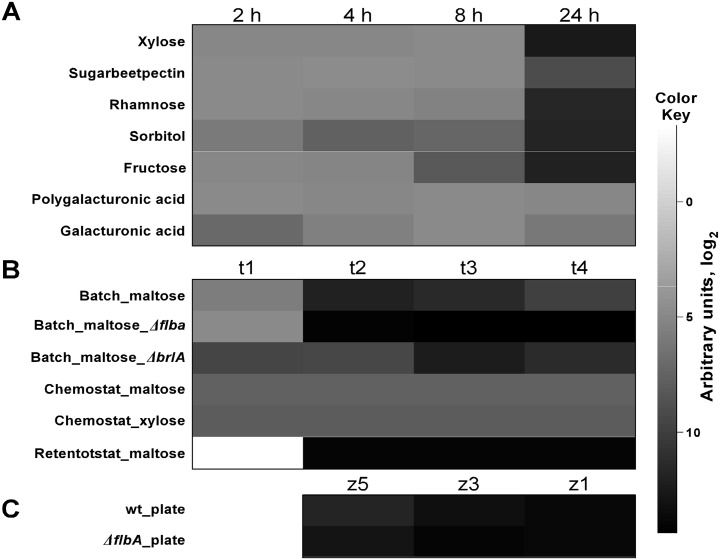
Expression levels of the *anafp* gene of *A*. *niger* strain N402 at different conditions. Transcription levels of the *anafp* gene were analyzed when exposed to different A) carbon sources in shaking flask cultures [23] and B) to carbon limitation in batch, retentostat [27] and chemostat bioreactor cultures [28]. *anafp* transcription levels are also depicted for batch cultivations in bioreactors with *ΔflbA* and *ΔbrlA* mutants, respectively [33]. Samples have been taken at four time points: t1–t4, exponential growth phase, 16 h, 60 h, 140 h post carbon depletion. In C) mRNA levels at different zones of a plated culture are depicted [33, 34]. Whereas zone 1 (z1) marks the center of the colony consisting, i.e. the oldest part of the culture, zone 5 (z5) marks the colony’s periphery. Zone 3 lies in between. Absolute values of transcription levels are depicted as arbitrary units of fluorescence intensity in a logarithmic scale and can be assessed using the legend. Color intensities were normalized to the highest absolute transcription level which is indicated by black color.

Given that carbon and nitrogen sources and availability have critical implications for fundamental fungal processes, such as metabolism, development, and cell morphology, we interrogated *anafp* transcript abundance among several existing datasets where global gene expression profiles have been compared between various carbon (glucose, fructose, xylose, sorbitol, rhamnose, sugarbeet pectin, galacturonic acid, polygalacturonic acid) and nitrogen (ammonium, nitrate) sources and concentrations. In total, 98 different genome-wide transcriptomic data were scrutinized that originated from ten independent submerged cultivations of *A*. *niger* (wild type strain N402 or derivatives thereof). These cultivations were performed in shake flask or bioreactor settings and provided data for both the exponential and stationary growth phase [23–32]. This survey revealed that (i) there was no detectable *anafp* expression during the exponential growth phase of *A*. *niger* (the measured fluorescence signal did not exceed the baseline level), (ii) that *anafp* expression is a response to severe carbon limitation (Fig 2A and 2B) and (iii) that *anafp* expression is independent of the nitrogen source (S1 Fig). Our own unpublished microarray data on a N402 derivative deleted for CpcA, the central regulator of the cross-pathway control network important to survive amino-acid starvation, also did not show altered *anafp* expression when compared to wild-type progenitor strain, further substantiating the conclusion that *anafp* expression is independent of the nitrogen source.

Interestingly, the *anafp* gene displays during submerged batch fermentations an expression profile similar to that of early starvation response genes such as *nagA* (An09g02240, predicted autolytic β-1,6 N-acetylglucosaminidase) and *atg1* (An04g03950, predicted Ser/Thr kinase involved in autophagy [29]). As for *nagA* and *atg1*, *anafp* expression peaked at 16 h after carbon depletion (corresponding to 4×10^3^ AU, see t2 Fig 2B) and gradually decreased during 60 h and 140 h post carbon depletion (see t3 and t4 Fig 2B). Most interestingly, the peak of *anafp*, *nagA* and *atg1* expression coincided with the emergence of a second hyphal population, displaying strongly reduced hyphal diameters (1 μm instead of 3 μm; [29]. Likewise, retentostat cultivation of *A*. *niger*, reaching specific growth rates of almost zero [27], strongly induced *anafp* expression. The severe carbon and energy limitation of retentostat cultivation resulted in two physiologically and morphologically distinct phases, during which *anafp* expression was up-regulated to signal intensities of 15.3×10^3^ and 16.5×10^3^ AU, respectively (Fig 2B): (i) slow vegetative growth of very thin hyphae at day 2 after switch to carbon limitation (μ = 0.014 h^-1^), and (ii) predominant asexual spore forming phase at day 8 after switch to carbon limitation (μ = 0.005 h^-1^). Common for the slow-growing cultures at day 2 and 8 was up-regulation of *nagA* and *atg1* as well as other genes involved in nutrient mobilization and with a predicted role for autophagy, N-acetylglucoseamine metabolism and carbohydrate transport [27].

**Fig 3:**
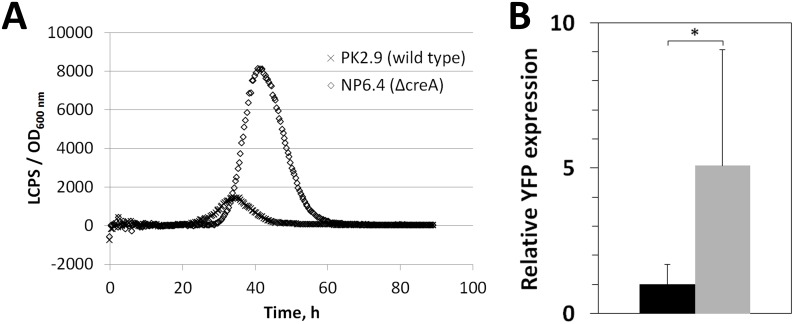
Analysis of *anafp* expression using a fluorescent reporter system. A) Luciferase expression under control of the *anafp* promoter was measured in a *ΔcreA* strain (NP6.4) and the corresponding wild type control strain (PK2.9) during 4 days of cultivation in complete medium using microtiter plates. Protein abundance was measured as luminescent counts per second normalized to culture optical density. Data of a representative experiment (n = 4) are shown. B) eYFP expression under control of the *anafp* promoter was measured in a *ΔcreA* strain (NP2.8, grey) and the corresponding wild type control strain (PK1.22, black) after 3 days of incubation on a complete medium agar. Expression levels were normalized to that of the control strain and are depicted as mean of three independent experiments performed as duplicate. *, p<0.03.

This meta-analysis of 98 *A*. *niger* microarray data indicated that up-regulated *anafp* transcription is a hallmark of severe carbon and energy limitation. We could confirm this conclusion by analyzing a luciferase reporter system, in which the luciferase gene was put under control of the *anafp* promoter (*Panafp*::*mluc*) and integrated into the genome of *A*. *niger* to replace the endogenous *anafp* gene. The resulting strain PK2.9 (*Panafp*::*mluc*, *Δanafp*), was cultivated under submerged conditions in microtiter plate based format. As summarized in S2 Fig, the *anafp* promoter became clearly activated during carbon starvation (about 14-fold) but only marginally during nitrogen starvation (about 2-fold). Based on this result we further hypothesized that transcription of the *anafp* gene might be under control of the carbon catabolite repressor CreA. Notably, eight CreA binding motifs are present within the 1000 bp upstream region of the *anafp* gene, which is twice than the statistical average present within all *A*. *niger* promoter regions according to the Transcription Factor Binding Site Finder (TFBSF) tool [19]. We confirmed a role for CreA in *anafap* regulation with two independent reporter systems. In the first approach, the *creA* gene was deleted in strain PK2.9 giving strain NP6.4. When both strains were cultivated in microtiter plates, a peak in luciferase activity between 40–50 h of cultivation was observed for both strains, whereby strain NP6.4 showed about 6-fold higher expression levels compared to strain PK2.9, thus clearly demonstrating that the *anafp* promoter is under control of CreA (Fig 3A). Note that the observed peak-like temporal expression pattern is in good agreement with the *anafp* expression profile observed for retentostat cultivations (see above and [35]). We also generated reporter strain PK1.22, in which expression of the fluorescent protein eYFP was put under control of the *anafp* promoter. The respective construct was integrated at the endogeneous *anafp* locus by which the *anafp* open reading frame was replaced by the *eyfp* gene (*Panafp*::*eyfp*, *Δanafp*). In this strain, the *creA* gene was deleted, giving strain NP2.8. As depicted in Fig 3B, quantification of eYFP fluorescence revealed a 5-fold higher mycelial fluorescence in the *creA* null strain compared to PK1.22, further substantiating the conclusion that CreA is the candidate regulator that represses *anafp* gene expression under sufficient carbon source availability.

### *anafp* expression is under FlbA control and a hallmark of carbon-starvation induced autolysis

Four independent studies reflecting eight different conditions analyzed the transcriptome of *A*. *niger* strain N402 in dormant conidia [36], during different stages of the germination process [36, 37], or in vegetative mycelium and aerial hyphae of a 7 day old culture grown on maltose [38]. The analysis of these microarray data did not reveal any *anafp* expression in dormant and germinating conidia but showed high intensity levels about 63-fold in the vegetative mycelium and about 227-fold in aerial hyphae compared to the baseline expression level (S1 Table). Interestingly, expression of chitin-remodeling enzymes including *ctcB*, *cfcI* and *nagA* were also expressed at relatively low levels in dormant and germinating conidia, but were highly induced in aerial hyphae (23-, 17- and 11-fold, [39]), thus displaying a similar expression profile as the *anafp* gene. As *A*. *niger* colonies show zonal differences with respect to gene expression, growth and protein secretion, one study scrutinized the transcriptomic landscape of an *A*. *niger* N402 colony at the periphery (zone 5), in the intermediate zone (zone 3) and the central zone (zone 1) and compared it with the spatial expression in a colony deleted for *flbA*. FlbA is a conserved protein in *Aspergilli* and its function is mainly to halt vegetative growth during conidiation and to activate the central regulator of asexual development BrlA in response to the extracellular signaling molecule FluG (for review see [40]). The aconidial, slow growing phenotype of Δ*flbA* is characterized by a fluffy appearance of the colony due to excessive formation of aerial hyphae. It lacks conidiophores, has thinner cell walls compared to the wild type, shows signs of cell wall stress and collapses due to autolytic disintegration of aerial hyphae in the center of the colony [33, 34]. As shown in Fig 2C, xylose-grown cultures of N402 displayed highest *anafp* expression in the center of the colony (zone 1, about 13.7×10^3^ AU), which gradually decreased towards the colony periphery (zone 3, about 9.5×10^3^ AU and zone 5, about 3.5×10^3^ AU). In agreement, a quantitative proteomics approach revealed that the amount of AnAFP secreted by the vegetative mycelium was about 8-fold higher in the central zone 1 and about 4-fold lower in the peripheral zone 5 when compared to the intermediate zone 3 [41]. Lack of *anafp* expression at the periphery of a colony were also reported by an independent study which interrogated tip-localized gene expression of *A*. *niger* in five individual hyphae [42]. Remarkably, deletion of *flbA* resulted in a pronounced increase of *anafp* expression in the intermediate and peripheral zone. Compared to the wild type, *anafp* expression increased only slightly in the center of the colony (3% in zone 1), but its expression drastically increased in the other two zones (65% in zone 3 and 110% in zone 5), suggesting that the slow growing and autolytic phenotype of the *ΔflbA* strain occurs concomitantly with augmented *anafp* expression (Fig 2C).

Van Münster et al. studied the consequences of *flbA* and *brlA* deletion for *A*. *niger* under bioreactor-controlled maltose-limited batch cultivations [33]. Note that deletion of *brlA* also results in an aconidial phenotype; however, aerial hyphae do not collapse in the middle of the colony [33]. Altogether, 12 transcriptomic samples were taken during four different cultivation time points (t1, exponential growth phase; t2–t4, 16 h, 60 h, 140 h post carbon depletion) for the *ΔflbA*, *ΔbrlA* and corresponding wildtype strain N402. As depicted in Fig 2B, *anafp* expression was lowest in all three strains during the exponential growth phase, although slightly higher in *ΔbrlA*. The strong increase of *anafp* expression in N402, which was observable at t2 after the shift to carbon starvation, became delayed in *ΔbrlA* (t3). Most impressively, *anafp* expression was between 4- to 22-fold higher in the autolysing *ΔflbA* strain during all three time points post carbon depletion. This data strongly suggested that the autolytic phenotype of the *flbA* mutant is somehow positively linked to *anafp* expression and corroborated the microarray data for colony growth of *A*. *niger* discussed above [34].

**Fig 4:**
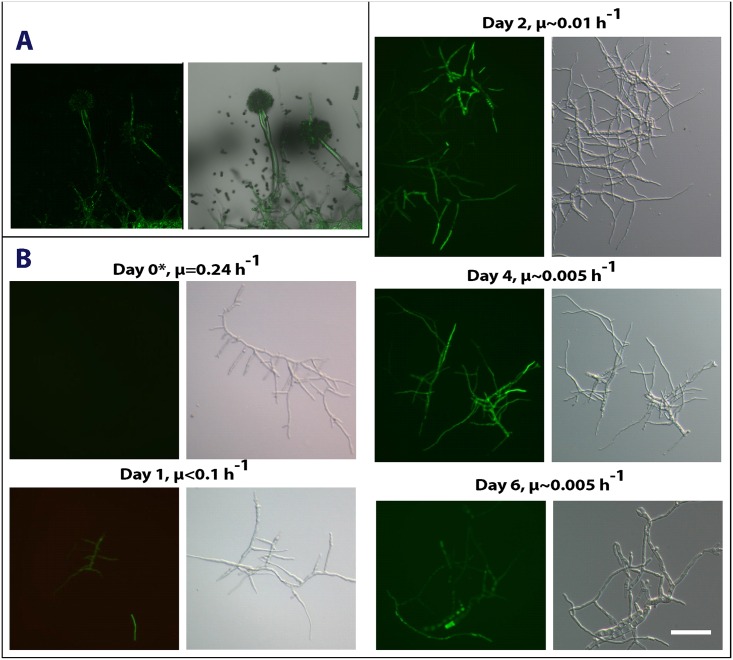
Fluorescence microscopic analysis of *anafp* promoter activity in reporter strain PK1.22. A) Shown is the fluorescence micrograph (left) and the fluorescence bright field overlay of strain PK1.22. CM agar sandwiched between two glass slides was inoculated with spores of strain PK1.22. After incubation at 30°C for two days in a humidified environment, the culture was analyzed using fluorescence microscopy. B) Shown are fluorescent (left column) and corresponding bright-field micrographs (right column) of culture samples taken from retentostat bioreactor cultivations of strain PK1.22 during exponential growth phase at day 0 with growth rate μ = 0.24 h^-1^ and decreasing growth rates after the switch to the retentostat cultivation as indicated. The activity of the *anafp* promoter was followed by monitoring the green fluorescence signal of eYFP. Bar = 20 μm.

To experimentally proof the hypotheses derived from this set of microarray data, we cultivated strain PK1.22 (*Panafp*::*eyfp)* on agar plates and under retentostat conditions and visualized the temporal expression pattern of the *anafp* gene via fluorescence microscopy. Indeed, *Panafp*::*eyfp* fluorescence signals are clearly evident in aerial hyphae and the underlying feeding vegetative substrate mycelium but not above baseline fluorescent levels in most conidia (Fig 4A). When strain PK1.22 was cultivated under retentostat conditions (Fig 4B), no eYFP fluorescence signals were detectable during the exponential growth phase (μ = 0.24 h^-1^), but became evident at day 1 after the switch to the retentostat cultivation mode (μ ~ 0.1 h^-1^) as predicted. Most interestingly, eYFP fluorescence was not evenly distributed in mycelia, but was restricted to only a few compartments. Such spatial control of *anafp* promoter activity was also evident at day 2 (μ ~ 0.01 h^-1^), day 4 (μ ~ 0.005 h^-1^) and day 6 (μ ~ 0.005 h^-1^), unambiguously demonstrating that *anafp* gene expression is tightly regulated with respect to time and space. Three important observations were made: (i) Highest fluorescence was detectable at day 2 and day 4 of retentostat cultivation and mainly occurred in two cellular populations: thin hyphae, thought to correspond to aerial hyphae in surface cultures [27], and in highly vacuolated compartments, potentially reflecting compartments undergoing autophagy; (ii) eYFP fluorescence preceded culture sporulation, which was first observed at day 4. This time difference was supported by previously published Northern data of Jorgenson *et al*. [27], which showed that *anafp* expression started at day 1, but *brlA* transcription at day 2 of retentostat cultivation; (iii) eYFP fluorescence ceased at day 6, whereby undetectable or weak fluorescence was observed in spores and in hyphae producing spores. These observations suggest that AnAFP is involved in processes preceding sporulation and not exploited as molecule to execute sporulation.

### Osmotic and oxidative stress affect *anafp* expression

Overall, 24 different microarray studies have been published that interrogated the responses of *A*. *niger* to different stress conditions, including secretion stress and cell wall stress and confrontation with nutrient competitors (S1 Table). In most of the studies, *A*. *niger* (N402 or derivatives thereof) was cultivated under controlled submerged conditions in bioreactors and stressed either during germination [37, 43, 44] or during the exponential growth phase [45]. Interestingly, none of the secretion stress agents (DTT, tunicamycin) and none of the cell membrane or cell-wall stressors applied in these studies (caspofungin, fenpropimorph, FK506, aureobasidin A, natamycin) provoked an increase in *anafp* expression. Likewise, neither forced overexpression of a heterologous secretion stress response gene (t-PA) [45] nor overexpression of the secretion stress regulator *hacA* [46] had any effect on *anafp* expression. Finally, deletion of two transcription factors important for securing cell wall integrity of *A*. *niger* (RlmA, [44]; TupA, [47] did also not affect *anafp* expression. Likewise, confrontation of *A*. *niger* with *Bacillus subtilis* [48] did not induce *anafp* transcription. Consequently, we hypothesize that *anafp* is not part of an *A*. *niger* defense mechanism to survive secretion or cell wall stress.

**Fig 5:**
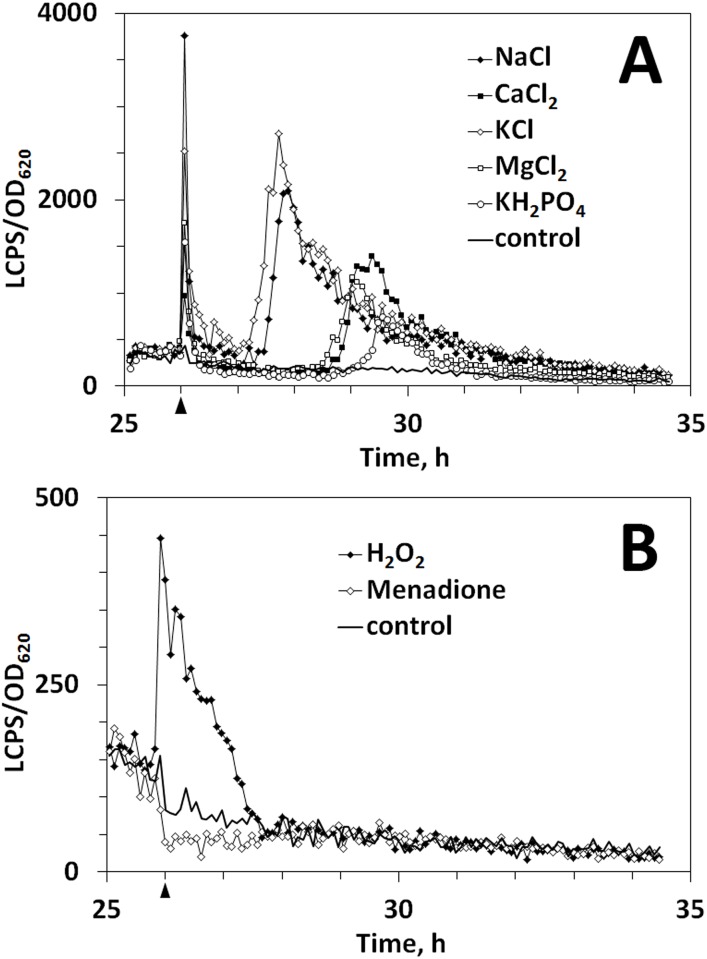
Influence of salt and oxidative stress on *anafp* promoter activity using a luciferase reporter system. **A)** Shown are the results of a representative cultivation experiment out of five using strain PK2.9 as reporter strain. Salt stress was induced 26 h after spore inoculation by addition of NaCl, KCl, CaCl_2_, MgCl_2_ and KH_2_PO_4_ at a final concentration of 100 mM (arrowhead). An untreated culture served as negative control. OD_620_ and luminescence (LCPS, luminescence counts per second) were measured periodically in order to detect *anafp* promoter activity. Note that salt addition also provoked a very immediate, sharp spike within seconds after addition, probably reflecting a transient cellular response which was not observed when simply water was added to the negative control. B) Shown are the results of a representative cultivation experiment of strain PK2.9 out of three. Oxidative stress was induced 26 h after spore inoculation by the addition of 5 mM H_2_O_2_ or 100 μM menadione, respectively (arrowhead). An untreated PK2.9 culture served as negative control. OD_620_ and luminescence (LCPS, luminescence counts per second) were measured periodically in order to detect *anafp* promoter activity.

To examine the impact of growth and stress conditions on *anafp* expression not covered by the available transcriptomic dataset, the luciferase reporter strain PK2.9 was subjected to salt stress using different mono- and bivalent salts, to oxidative stress provoked by H_2_O_2_ or menadione, to different pH and temperature conditions, cell wall stressors, and growth under light and dark conditions. The influence of salt stress on *anafp* promoter activity was investigated using NaCl, CaCl_2_, KCl, MgCl_2_ and KH_2_PO_4_. About 1 h after stress induction through NaCl and KCl, the *anafp* promoter became activated, as LCPS/OD values started to rise continuously (Fig 5A). Maximum LCPS/OD values were reached at about 2 h after stress induction. Afterwards LCPS/OD values decreased again. The addition of CaCl_2_, MgCl_2_ and KH_2_PO_4_ led to an increase in *anafp* promoter activity at about 2–3 h after stress induction as LCPS/OD values rose quickly and reached a maximum between 0.5–1 h later before the values decreased again. Notably, stress induced by salts consisting of monovalent cations (Na^+^, K^+^) led to an earlier response compared to salts consisting of bivalent cations (Mg^2+^, Ca^2+^) when chloride was used as an anion. Interestingly, the response to potassium phosphate was clearly delayed when compared to potassium chloride, suggesting that both ion types (cations and anions) are able to modulate *anafp* gene expression during salt stress. The negative control (cells treated with salt free water) did not display any *anafp* promoter activity, indicating that increased LCPS/OD values were generated in response to salt addition (Fig 5A).

To determine whether oxidative stress affects *anafp* promoter activity, H_2_O_2_ and menadione were added to PK2.9 exponentially growing cultures. A representative graph is shown in Fig 5B. After stress induction with H_2_O_2_, the *anafp* promoter became rapidly activated as LCPS/OD values increased quickly to a maximum. Afterwards LCPS/OD values decreased constantly during the next 2 h. Interestingly, the opposite response was observed for menadione, where LCPS/OD values decreased quickly after stress induction and were below those of the negative control for at least 2 hours, indicating an inhibition of *anafp* promoter activity. Such an opposing effect of the two tested oxidative compounds is in good agreement with findings of Nitsche *et al*. who investigated autophagy deficient *A*. *niger* strains. It was found that autophagy mutants were more sensitive to H_2_O_2_, while their resistance to menadione was increased [49]. This finding is consistent with respect to *anafp* gene expression profile during oxidative stress, and provides further evidence that the function of AnAFP appears to be linked to the process of autophagy.

The cultivation of *A*. *niger* PK2.9 at different pH values (5.8, 7.0, 8.0 compared to 3.0) or temperatures (21°C, 40°C compared to 30°C) as well as its cultivation at constant lightness, constant darkness or darkness interrupted with light pulses was not found to influence *anafp* promoter activity. Also, treatment of PK2.9 with compounds interfering with cell wall integrity of *A*. *niger* including caspofungin (20μg/ml), AFP (20μg/ml), FK506 (20 ng/ml), fenpropimorph (10 μg/ml) and calcofluor white (200 μg/ml) did not provoke any significant *anafp* response (data not shown), further demonstrating that *anafp* gene expression selectively responds to osmotic and oxidative stress conditions but not to cell wall stress.

### *A*. *niger* deleted for the *anafp* gene lacks a clear phenotype

Given the above data, a gross morphological or growth phenotype for an *anafp* deletion strain would be unlikely. Indeed, when we compared the *anafp* deletion strain CH4.2 with its parental wild type strain MA87.6, we could not detect any differences between both strains with respect to resistance to salt stress (1 M NaCl), osmotic stress (1.2 M sorbitol) and cell wall stress (0.1 mg/ml calcofluor white) (S3A Fig and S1 Table).

In addition, neither germination (timing and germination rate), sporulation (timing and efficiency) nor growth rate were affected by the *anafp* deletion. Furthermore, deletion of the *anafp* gene in a *ΔcreA*, *ΔflbA* and *ΔbrlA* strain, respectively, did also not affect the slow growing or autolysing phenotype of *ΔflbA*, nor the bristle phenotype of the *ΔbrlA* strain, when cultivated on medium agar plates (S3B Fig). Likewise, cultivation of these strains under submerged conditions did not reveal any significant differences with respect to biomass formation. The only evident effect was strong acidification of the culture medium in the strain deleted for both *anafp* and *brlA* (NP3.2) but not in the strains individually deleted for *anafp brlA*, *flbA* and *creA*, respectively (S3C Fig). The absence of an unambiguous *anafp* deletion phenotype could suggest (i) that its phenotype might be undetectable in the described assays, (ii) that AnAFP is involved in processes, which have not been assessed in the assays used and/or (iii) that other redundant proteins might have taken over the function of AnAFP.

### Functional analysis of AnAFP using gene co-expression network analysis

Gene co-expression network analysis is a powerful approach for the functional annotation of uncharacterized genes. This approach aims to identify genes with a consistent, correlated expression pattern across phenotypically diverse samples or experimental conditions. Genes within shared expression profiles are tightly connected and are predicted to function in the same regulatory and/or functional pathway (“guilt-by-association” approach; [50, 51]). In order to identify tightly connected genes in *A*. *niger*, we used Bioconductor [52] to calculate pairwise correlation coefficients (Spearman coefficient) for the expression profiles of all ~ 14.000 *A*. *niger* genes under the 155 different cultivations present in our in-house transcriptomic database. Pairs with a Spearman score higher than or equal to 0.5 were considered to be significantly co-expressed (see methods section for details). This gene co-expression network was scrutinized for genes displaying correlated gene expression with the *anafp* gene. As summarized in S2 and S3 Tables, 605 / 198 genes showed a positive correlation with the *anafp* gene (Spearman coefficient ≥ 0.5 / ≥ 0.6) and 381 / 38 genes a negative correlation (Spearman coefficient ≥−0.5 / ≥−0.6).

Gene ontology (GO) enrichment analysis of the 605 positively correlated genes using the FetGOat tool [20] revealed that the processes being positively correlated with *anafp* expression belong to developmental process (GO:0051094), cellular polysaccharide catabolic process (GO:0044247), antioxidant activity (GO:0016209) and O-glycosyl hydrolase activity (GO:0004553) (S4A and S4B Fig), whereas processes being negatively related with *anafp* expression (381 genes) include translation (GO:0006412), amino acid biosynthetic process (GO:0008652), nucleobase biosynthetic process (GO:0046112) and pigment biosynthetic process (GO:0046148) (S4C–S4E Fig).

Table 2 lists selected genes which are among GO enriched cohorts positively correlated with *anafp* expression. These genes are a hallmark of severe carbon and energy limitation in *A*. *niger* as recently reported by [27, 29, 49]. These processes include genes predicted to regulate and mediate endogenous nutrient mobilization during stationary growth, osmotic and oxidative stress, *e*.*g*. An18g03170 (predicted *S*. *cerevisiae* ortholog of the cyclin-dependent kinase Pho85), An01g0330 and An05g00190 (cyclins Pcl1 and Pcl7 of Pho85), glycogen catabolic enzymes (orthologs of *S*. *cerevisiae* Gph1 and GdbA, respectively), genes predicted to encode transcription factors controlling the utilization of amino acids (proline, serine, threonine) and fatty acids (*e*.*g*. orthologs of *S*. *cerevisiae* Cha4, Put3, Oaf1, Oaf3). Furthermore, the expression of autophagy-related genes which are likely to mediate the fueling of conidiation during nutrient starvation (orthologs of *S*. *cerevisiae* Atg4, Atg8 and *A*. *nidulans* metacaspase) correlate with *anafp* expression. In agreement with a role of AnAFP in conidiation, one of the key developmental switches controlling conidiation in filamentous fungi, StuA, is co-expressed with *anafp*. StuA is an APSES transcription factor shown in *A*. *nidulans*, *A*. *fumigatus*, *Acremonium chrysogenum* and *Penicillium chrysogenum* which plays a critical role in the control of conidiophore formation [53–56]. Most interestingly, StuA has been proven to regulate secondary metabolite biosynthesis in *A*. *fumigatus*, *A*. *chrysogenum* and *P*. *chrysogenum*. For example, six secondary metabolite gene clusters were found to be StuA regulated in *A*. *fumigatus* [57], in addition to the penicillin gene cluster in *P*. *chrysogenum* (Sigl et al., 2011) and cephalosporin gene cluster in *A*. *chrysogenum* [55]. The fact that the correlated *anafp* gene network contained 19 fungal homologs of secondary metabolite gene clusters predicted to function in penicillin, sterigmatocystin and lovastatin biosynthesis (Table 2) suggests a similar function of StuA for conidiation and secondary metabolism in *A*. *niger*, and links the *anafp* gene with secondary metabolite genes. In this respect it is interesting to note that also VelC belongs to the *anafp* co-expressed gene network (Table 2). VelC is a member of the *velvet* protein family (VelA, VelB, VelC and VosA), which are fungal-specific transcription factors containing a NF-χB-like DNA binding domain and coordinating secondary metabolism with developmental processes such as (a)sexual sporulation, fruiting body development and sclerotia formation [58]. As VelC is a repressor of BrlA and VosA [59] and the VosA-VelB heterocomplex turns-off β-glucan synthesis in (a)sexual spores of *A*. *nidulans* [60] one might be tempted to speculate that VelC is a negative regulator of *anafp* and other cell wall genes which belong to the *anafp* gene network such as the chitin synthase genes *chsB* and *csmB* (Table 2).

**Table 2:** Genes being positively correlated with *anafp* expression as inferred from 155 cultivation conditions.

ORF and functional group^a^		Predicted gene function^a^	Spearman coefficient
**Development**			
An05g00480	similarity to transcription factor StuA–*A*. *nidulans*		0.6
An04g07320	similarity to velvet family transcription factor VelC–*A*. *nidulans*		0.6
**Secondary metabolites**			
An16g06270	similarity to versicolorin reductase VerA—*A*. *nidulans*		0.65
An04g04340	similarity to nonaketide synthase of the lovastatin biosynthesis LovB—*A*. *terreus*		0.5
An07g01200	similarity to enoyl reductase of the lovastatin biosynthesis LovC—*A*. *terreus*		0.6
An09g01880	similarity to enoyl reductase of the lovastatin biosynthesis LovC–*A*. *terreus*		0.65
An11g06440	similarity to enoyl reductase of the lovastatin biosynthesis LovC–*A*. *terreus*		0.55
An16g01630	similarity to enoyl reductase of the lovastatin biosynthesis LovC–*A*. *terreus*		0.5
An15g02130	similarity to lovastatin diketide synthase LovF–*A*. *terreus*		0.65
An02g08570	similarity to isopenicillin N acyltransferase AatA–*A*. *nidulans*		0.55
An18g01180	similarity to penicillin V amidohydrolase PVA—*Fusarium oxysporum*		0.6
An03g06460	similarity to sterigmatocystin biosynthesis p450 monooxygenase *stcB*–*A*. *nidulans*		0.5
An01g14020	similarity to sterigmatocystin synthesis transcription regulator *aflR*–*A*. *nidulans*		0.5
An18g01200	similarity to O-methylsterigmatocystin oxidoreductase Ord1 –*A*. *flavus*		0.55
An03g01460	similarity to gibberellin 7-oxidase—*Cucurbita maxima*		0.55
An12g05380	similarity to cercosporin transporter CFP—*Cercospora kikuchii*		0.65
An02g13750	similarity to glutaminase A GtaA involved in ochratoxin A biosynthesis–*A*. *oryzae*		0.65
An01g06930	similarity to polyketide synthase FUM5—*Gibberella moniliformis*		0.6
An01g12050	similarity to 15-decalonectrin 15-O-acetyltransferase TRI3 of the trichothecene pathway–*F*. *sporotrichioides*		0.55
An12g02490	similarity to aflatoxin biosynthesis regulator AflR–*A*. *flavus*		0.55
An01g00680	similarity to cytochrome P450 monooxygenase AvnA of the aflatoxin biosynthesis–*A*. *parasiticus*		0.5
**Cell wall biosynthesis and remodeling**			
An09g04010	class III chitin synthase ChsB		0.5
An02g02340	class V chitin synthase CsmB		0.5
An01g05360	chitinase CfcD		0.55
An01g06500	α-1,6 mannanase DfgD		0.5
**Membrane lipid metabolism**			
An09g02180	phospholipase A1		0.65
An16g03700	similarity to phospholipase B–*A*. *oryzae*		0.6
An16g01880	similarity to lysophospholipase–*A*. *foetidus*		0.6
An07g08980	similarity to phosphatidylinositol 3-phosphate 5-kinase Fab1 –*S*. *cerevisiae*, generates phosphatidylinositol (3,5)P2 important for osmotic stress response and vesicle-mediated membrane trafficking and cell wall integrity		0.55
An01g06830	similarity to 3-ketosphinganine reductase Tsc10 –*S*. *cerevisiae*, involved in phytosphingosine synthesis		0.5
**Sensing and signaling**			
An01g09460	similarity to plasma membrane sensor WscA–*A*. *nidulans*, important for osmotic stress sensing		0.5
An03g04690	similarity to osmosensor Sho1p –*S*. *cerevisiae*, involved in activation of the Cdc42p- and MAP kinase-dependent filamentous growth pathway and the high-osmolarity glycerol (HOG) response pathway		0.5
An04g07460	similarity to pH sensor PalF–*A*. *nidulans*, involved in alkaline pH signaling		0.6
An01g06900	similarity to activator of stress genes Asg1 –*S*. *cerevisiae*		0.6
An16g00750	similarity to transcription activator Cha4 –*S*. *cerevisiae*, involved in serine and threonine utilization		0.55
An04g06000	similarity to transcription activator Put3 –S. cerevisiae, involved in proline utilization		0.5
An05g00020	similarity to transcription factor Oaf1 –*S*. *cerevisiae*, involved in beta-oxidation of fatty acids		0.5
An04g08620	similarity to repressor Oaf3 –*S*. *cerevisiae*, involved in beta-oxidation of fatty acids		0.5
An01g13700	similarity to transcription factor Crz1 –*S*. *cerevisiae*, involved in calcium signaling and cell wall integrity		0.55
**Nutrient mobilization and autophagy**			
An18g03170	similarity to cyclin-dependent kinase Pho85—*S*. *cerevisiae*, involved in regulating the cellular response to nutrient levels and environmental conditions		0.5
An05g00190	similarity to cyclin Pcl7 –*S*. *cerevisiae*, interacts with cyclin-dependent kinase Pho85 to control glycogen storage		0.5
An01g03330	*pclA*, similarity to cyclin Pcl1 –*S*. *cerevisiae*, interacts with cyclin-dependent kinase Pho85		0.6
An08g05790	similarity to glycogen phosphorylase Gph1 –*S*. *cerevisiae*, required for the mobilization of glycogen during stationary growth, osmotic or oxidative stress		0.55
An01g06120	glycogen debranching enzyme GdbA		0.5
An07g10020	similarity to autophagy-related ubiquitin modifier Atg8 –*S*. *cerevisiae*		0.5
An11g11320	similarity to autophagy-related cysteine protease Atg4 –*S*. *cerevisiae*		0.5
An11g05400	similarity to metacaspase casA–*A*. *nidulans*		0.6
**Self / nonself recognition**			
An18g00980	similarity to G-protein-coupled receptor protein PTH11—*Magnaporthe grisea*, involved in host recognition		0.7
An13g01870	similarity to G-protein-coupled receptor protein PTH11—*Magnaporthe grisea*, involved in host recognition		0.7
An12g01300	similarity to vegetative incompatibility protein tol—*Neurospora crassa*		0.75
An15g04830	similarity to vegetative incompatibility protein tol–*N*. *crassa*		0.55
An11g09180	similarity to vegetative incompatibility protein tol—*N*. *crassa*		0.55
An12g02900	similarity to heterokaryon incompatibility protein het-6—*N*. *crassa*		0.65
An07g04330	similarity to heterokaryon incompatibility protein het-6—*N*. *crassa*		0.65
An08g09970	similarity to beta-transducin-like protein het-e—*Podospora anserina*		0.6
An04g00900	similarity to beta transducin-like protein het-e1 –*P*. *anserina*		0.6
An02g01260	similarity to beta transducin-like protein het-e1 –*P*. *anserina*		0.55
An12g00850	similarity to beta transducin-like protein het-e1 –*P*. *anserina*		0.5

^a:^ after [16], Aspergillus Genome Database (AspGD, [15]) and the Saccharomyces Genome Database (SGD, [61]).

To our surprise, eleven genes whose predicted function is involved in self/nonself recognition in *Neurospora crassa*, *Podospora anserina* and *Magnaportha grisea* belong to the *anafp* gene co-expression network. Hyphal fusion can occur within a mycelial colony or between colonies of the same or different species. As a result, different nuclei (heterokaryon) can be present in a cytoplasm and genes involved in allorecognition determine the fate of the heterokaryon. If the genotypes are incompatible, heterokaryotic cells become rapidly compartmentalized and subjected to a type of programmed cell death called vegetative (heterokaryon) incompatibility. This process has mainly been studied in *N*. *crassa* and *P*. *anserina* (for reviews see [62, 63]. Three out of eleven *het* loci (each having at least two to three different alleles) have been studied in *N*. *crassa* (*tol*, *het-6*, *pin-c*). For *tol* and *het-6*, three and two orthologs (potential alleles), respectively, are members of the *anafp* gene co-expression gene network. Notably, An12g01300, which is an ortholog of *tol*, is the highest *anafp*-correlated gene of *A*. *niger* (Spearman coefficient 0.75, Table 2).

### The evolution of antifungal proteins

**Fig 6:**
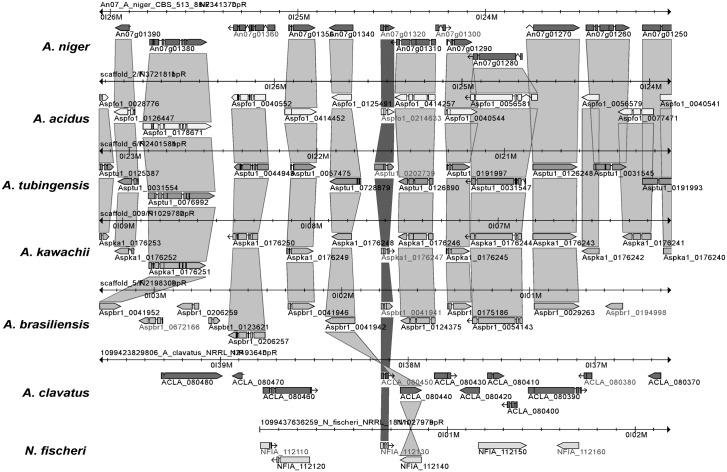
Jaccard orthologous clusters of multiple genomes. Depicted are genome excerpts of different *Aspergilli* species comprising ±15 kb around the *anafp* gene locus. The *anafp* gene and its orthologues are marked by a dark grey ribbon, whereas all other orthologous gene clusters up- and downstream of the *anafp* gene are marked by light grey ribbons. Corresponding genes are marked with their locus names and are depicted as horizontal bars and arrows showing gene orientation. Horizontal axes on top of each genome excerpt show the relative localization on the chromosomes in mega bases.

In order to identify AnAFP orthologs in other fungal species, the predicted sequence of AnAFP (An07g01320) was used for BLASTP analyses against protein sequences deposited in AspGD (www.aspergillusgenome.org) and NCBI (www.blast.ncbi.nlm.nih.gov) databases. BLASTP searches against 20 other *Aspergillus* species housed at AspGD predicted that AnAFP orthologs are also present in the black Aspergilli *A*. *tubingensis*, *A*. *acidus*, *A*. *kawachii* and *A*. *brasiliensis*, but are also encoded in the genomes of *A*. *clavatus* and *Neosartorya fischeri* (Fig 6). Interestingly, the An07g01320 gene is part of a 100 kb syntenic genome region in five out of seven species (*A*. *niger*, *A*. *tubingensis*, *A*. *acidus*, *A*. *kawachii* and *A*. *brasiliensis*) as identified using a Jaccard clustering approach provided on AspGD [15].

**Fig 7:**
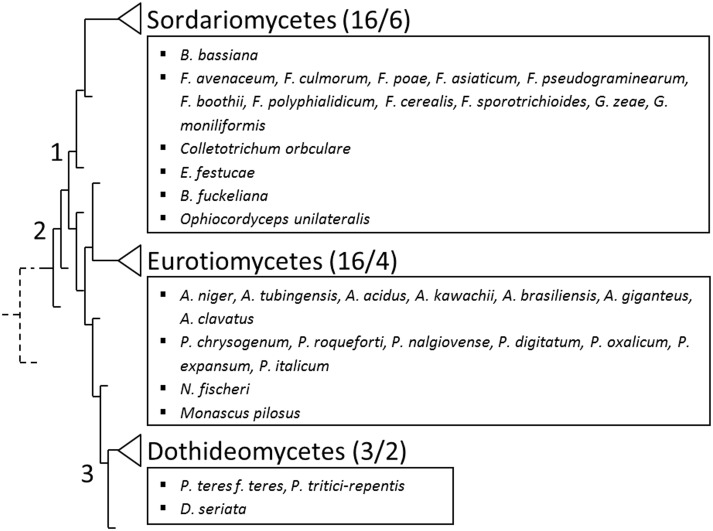
Species owning AnAFP orthologs within the *Ascomycota* tree of life. Depicted is an excerpt of the *Ascomyota* tree of life according to Schoch *et al*., 2009 [64]. Species with AnAFP orthologs are listed according to their class. Corresponding superclasses are indicated by numbers 1–3. 1, *Sordariomyceta*; 2, *Leotiomyceta*; 3, *Dothideomyceta*. Numbers of species/genus are indicated in brackets. Dashed line represents the last common ancestor of the three superclasses.

BLASTP searches using NCBI databases revealed AnAFP orthologs outside the genus *Aspergillus*. In total, 49 AnAFP orthologs are encoded in 35 fungal species all belonging to the phylum *Ascomycota* and include e.g. *Penicillium* and *Fusarium* spp., *Monascus pilosus*, *Gibberella zeae (*teleomorph of *F*. *graminearum)*, *Diplodia seriata*, *Epichloe festucae*, *Beauveria bassiana*, *Botryotinia fuckeliana*, *Colletotrichum orbiculare*, *Ophiocordyceps unilateralis* and *Pyrenophora spp* (Fig 7). Recently, Schoch *et al*. have generated an *Ascomycota* tree of life providing the most diverse and complete *Ascomycota* phylogenetic analysis to date [64]. It spans all 15 currently recognized classes and includes representatives for 90% of the currently recognized orders in the AFTOL (Assembling the Fungal Tree of Life) classification [65]. So far, we have identified AFP orthologs in three out of seven superclasses, *i*.*e*. *Sordariomyceta*, *Leotiomyceta* and *Dothideomyceta* (Fig 7). Within the three superclasses, the class of *Sordariomycetes* harbors the highest number of genera encoding own AnAFP orthologs.

**Fig 8:**
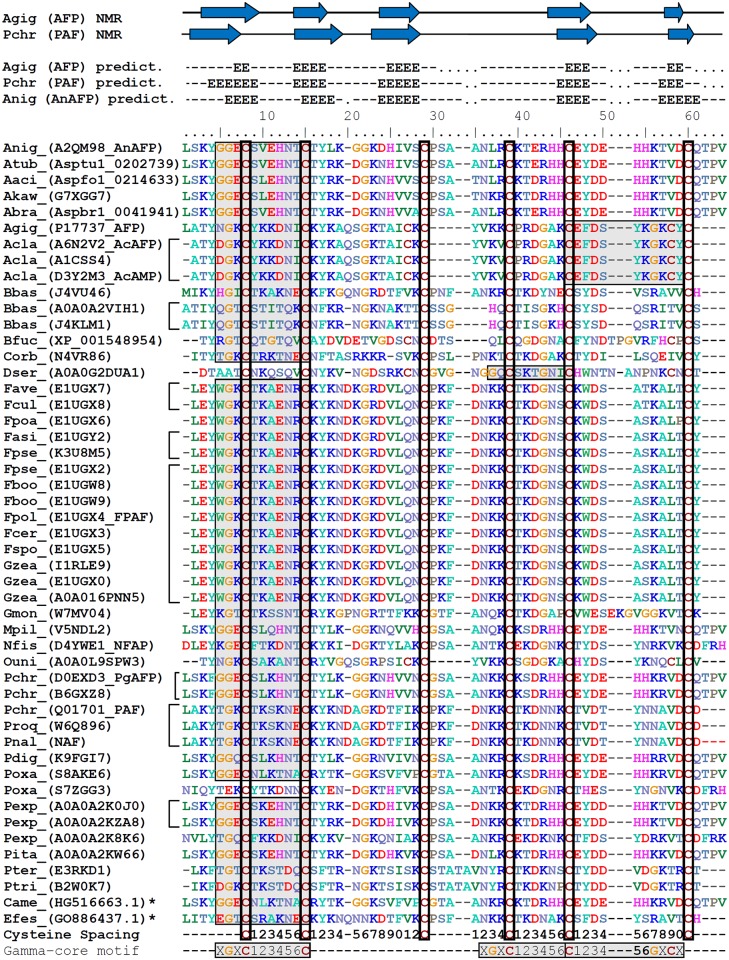
Alignment of primary and secondary structures of AnAFP orthologs. Identified or proposed amino-acid sequences of mature proteins were aligned by CLUSTAL W. Names of corresponding organisms owning the orthologue are abbreviated using the first letter of genus name followed by the first three letters of the species name. Protein/GenBank accession numbers from UniProtKB, AspGD or NCBI, respectively, are indicated in brackets. Amino-acid sequences marked by asterisks were derived from an expressed sequence-tag database analysis. Though some of the listed mature proteins are identical (marked by square brackets), all of them differ at least in one amino acid when considering the pre-pro-regions as well. The overall conserved cysteine residues are highlighted by open boxes. Predicted γ-core motifs are highlighted by shaded boxes. Secondary structures derived from NMR solution structures of AFP [70] and PAF [71] and from a secondary structure prediction of AFP, PAF or AnAFP, respectively, are shown at the top. Secondary structure predictions have been calculated using the PredictProtein server [66] for all mature proteins. Blue arrows and capital E-letters on top depict positions of beta strands whereas lines mean loops or turns, respectively. Gaps are depicted by dots.

The primary structure alignment of 48 orthologs from the 35 *Ascomycetes* species clearly showed that the cysteine-spacing pattern CX_(6)_CX_(11–12)_CX_(4–9)_CX_6_CX_10–13_C characteristic for the *A*. *giganteus* AFP and the *P*. *chrysogenum* PAF is conserved in all orthologs (Fig 8). A secondary structure prediction for the sequences of the corresponding mature proteins using the PredictProtein server [66] showed that 48 orthologs possess almost identical β-strand motifs, which are characteristic for AFP and PAF (Fig 8). A γ-core motif can be found in all AnAFP orthologs, except for *P*. *oxicalum* (Fig 8), implying that the majority of AnAFP orthologs are indeed membrane-interacting proteins which likely exert antimicrobial / antifungal activities as proven for AnAFP, AFP, PAF, AcAFP and NFAP [13, 22, 67–69]. Most of the γ -core motifs are located N-terminally, with the only exception of the AnAFP ortholog of *D*. *seriata*, where the γ-core motif localizes to the central part of the protein. Interestingly, a second motif is present in its levomeric isoform 2 [1] in the *A*. *giganteus* AFP and the *A*. *clavatus* AcAFP.

We screened NCBI’s EST database using TBLASTN in order to identify AnAFP orthologs outside the fungal kingdom. However, only one AnAFP ortholog was found, which was present in pearl millet *Pennisetum glaucum* and identified most recently by Choudary *et al*. to be expressed under drought stress [72]. The plant ortholog contains a γ-core motif and displays 77% sequence similarity to the *A*. *niger* AnAFP but 47% only to *A*. *giganteus* AFP (Fig 8). This was the only AnAFP orthologous protein found outside the fungal kingdom, which suggests that the most common ancestor of AnAFP is from the phylum *Ascomycota*, and that the plant orthologous protein might have originated from fungi and was acquired via horizontal gene transfer and/or it evolved convergently [2].

## Discussion

Cysteine-containing antimicrobial peptides of diverse phylogeny share the structural γ-core motif, which is the major determinant of their membrane-based activity [2, 3]. We here provide evidence that AnAFP of *A*. *niger* has membrane-permeabilising activities which is likely causatively linked with the presence of a γ-core motif in its N-terminal region. We furthermore identified AnAFP orthologs in 48 different fungal species belonging to the *Ascomycota* phylum, 47 of which harbor the γ-core domain. Such high occurrence of AnAFP orthologs implicates an important biological function for the respective producing strain and could explain the evolutionary preservation of this protein. It will only be a matter of time before the availability of more fungal genomes will enable further identification of AnAFP orthologs. As the *A*. *giganteus* AFP was the first antifungal protein discovered from *Ascomycetes* [73] and is the best studied antifungal protein with respect to structure, antifungal mode-of-action and fungal defense strategies [8, 21], we propose to name the group of AnAFP orthologous proteins after its founding member ‘AFP family’.

Given that antimicrobial peptides are widely ustilised by organisms throughout multiple kingdoms, it was remarkable that we could identify only one AFP homolog outside the fungal kingdom. In the plant *Pennisetum glaucum* (pearl millet), an endogenous AFP homolog was found to be present and specifically expressed under drought stress, *i*.*e*. nutrient limitation [72]. Interestingly, although the *P*. *glaucum* AFP displays high sequence similarity to the fungal AFPs, the plant is still highly sensitive to fungal attacks. Surprisingly, heterologous expression of the *A*. *giganteus afp* gene in *P*. *glaucum* increased plant`s resistance by up to 90% [74]. This observation might suggest that the plant AFP adopted additional cellular role(s) for *P*. *glaucum* which is/are apart from its original antifungal activity and thus not sufficiently protective against fungal infections anymore.

Our meta-analysis of available *A*. *niger*–omics data together with the establishment and evaluation of the deduced *anafp* gene co-expression network has revealed exciting new insights into *anafp* expression and propose additional functions for AnAFP which are distinct from its antifungal activity. However, the insights gained raise as many questions as they answer. First, expression of the *anafp* gene is under negative control of CreA, which we proved by analyzing *anafp* expression using a luciferase-based and eYFP-based reporter system in a wild type and *ΔcreA* background. Second, the transcriptomic data strongly suggest that one function of AnAFP is important for the survival of *A*. *niger* during nutrient, mainly carbon limitation, where it becomes derepressed. Its expression profile resembles that of early starvation response genes *nagA* and *atg1* as well as genes of chitin-remodeling enzymes including *ctcB*, *cfcI* and other genes involved in nutrient mobilization and with a predicted role for autophagy, N-acetylglucoseamine metabolism and carbohydrate transport. An interaction of AnAFP with the autophagic machinery was further corroborated by the gene-correlation network analysis, which uncovered Atg4, Atg8 and Pho85 orthologs as being coexpressed with the *anafp* gene, known to control and execute autophagy in *S*. *cerevisiae*. In this respect it is interesting to note that the *anafp* promoter was specifically induced (or derepressed) in highly vacuolated compartments and in foraging hyphal cells during carbon starvation (Fig 4). Our survey of published semi-quantitative proteomics data for the culture filtrates of the N402, *ΔflbA* and *ΔbrlA* bioreactor cultivations [33] revealed that AnAFP was only detectable at 140 h post carbon depletion in the *ΔflbA* strain (S1 Table) [33]. This is in fact true for a number of hydrolytic cell wall enzymes including chitinases and mannanases that had strongly up-regulated transcription during carbon starvation but were not detectable in the culture filtrates [33], suggesting that these enzymes as well as AnAFP might rather function intracellularly during carbon-starvation induced autophagy. Although hypothetical at this point, we speculate that AnAFP thus exerts two functions during nutrient limitation: (i) it acts intracellularly and assists in committing compartments to autophagy. (ii) AnAFP acts extracellularly and becomes secreted by foraging hyphae in order to inhibit the growth of fungal nutrient competitors in the vicinity. Another observation further pointing to a functional link of AnAFP with autophagy is the opposing response of the *anafp* promoter when *A*. *niger* was stressed with the two oxidative compounds H_2_O_2_ and menadione (Fig 5). This response is concomitant with the opposing response of autophagy-deficient *A*. *niger* mutants (*Δatg1* and *Δatg8*) to both compounds [49]. Although not mechanistically understood, these mutants showed an increased and decreased sensitivity towards H_2_O_2_ or menadione when compared to the wild type, which suggested that autophagy may somehow protect or sensitize *A*. *niger* against to these compounds, respectively. Notably, the *P*. *chrysogenum* PAF was recently found to be involved in the regulation of autophagy processes in its host [75]. Kovács *et al*. showed that *Δpaf* mutant strains have significantly reduced apoptosis rates which could be rescued by PAF addition. The reduction of apoptosis capacity in the *Δpaf* mutants was accompanied by the absence of autophagosomes. Moreover, the *Δpaf* mutant showed reduced transcript levels of several autophagy-related genes. PAF and AnAFP share only about 47% similarities in their primary structures (Fig 8), which could explain why both proteins function in the same pathway but to a different extent.

Growth inhibition, compartmentalization of hyphal cells, vacuolization and massive cell death are also common features of incompatible heterokaryotic fungal cells which die approximately 30 min post fusion [62]. Although heterokaryon incompatibility (HI) of *A*. *niger* strains is a well-known phenomenon, it is poorly characterized at the molecular level, with only the *het-c* gene functionally studied so far. Unfortunately, variation of *het-c* among 99 black *Aspergillus* strains taken from a global population was minor, and it was impossible to experimentally link HI to the *het-c* locus in *A*. *niger* [76]. As the *anafp* co-expression network contains several genes predicted to encode known HI genes from *N*. *crassa* and *P*. *anserina*, we thirdly deduce that AnAFP may not only be important for autophagic processes during nutrient limitation but is likely involved in self/nonself recognition during hyphal fusion, where it causes death of fused cells if they are genetically dissimilar. Supporting evidence for this working hypothesis is the fact that AnAFP does not inhibit the growth of *A*. *niger* and its plasma membrane resists the membrane-permeabilizing activity of AnAFP (Fig 1 and Table 1). Hence, *A*. *niger* is able to distinguish between its own and alien antifungal proteins which could provide the mechanistic basis for different cellular processes into which AnAFP is embedded. These processes are distinct in their triggering signals (nutrient limitation *versus* genetic dissimilarity) but similar with respect to their cellular response and execution (cell death). Like *A*. *niger*, *A*. *giganteus* and *P*. *chrysogenum* are also only moderate sensitive towards their own antifungal proteins AFP and PAF, respectively, [22, 67], implying that AFP producing strains possess innate sensing or defense systems which enable them to discriminate between AFPs from self or nonself origin. Such ability would be key to exploit these proteins during the process of hyphal fusion.

The meta-analysis of transcriptomic data uncovered fourthly, that one function of AnAFP is strongly linked to developmental processes and secondary metabolism, *i*.*e*. when *A*. *niger* becomes committed to asexual development. Nutrient limitation in the vegetative mycelium of *Aspergilli* is a basal trigger for reproductive development which requires nutrient mobilization from internal stores, *e*.*g*. by recycling of own biomass [29, 40]. Here, the function of AnAFP becomes important. It is expressed in submerged cultures when the growth rate drops below a certain level (μ = 0.16 h^-1^) and displays its highest expression in the center of a surface colonies, both reflecting severe carbon source limitation. Compared to the wild type, *anafp* expression is two- to tenfold upregulated in a *ΔbrlA* or *ΔflbA* background, respectively. As the *ΔflbA* mutant displays an autolytic phenotype, provides further evidence that AnAFP is likely exploited by *A*. *niger* to support autolysis in the center of a colony to fuel aerial hyphae formation. Note that *anafp* expression precedes *brlA* transcription; hence BrlA cannot be a regulator of *anafp*.

Likely developmental regulators of *anafp* expression are the APSES transcription factor StuA and the NF-χB-like transcription factor VelC as predicted by the gene-correlation network. Both function upstream of BrlA and FlbA and coordinate asexual development with secondary metabolism in *Aspergillus* [40, 58]. Although the *anafp* promoter does not contain a StuA binding motif (the VelC binding motif is unknown), 114 genes of the *anafp* gene correlation network contain StuA binding sites in their promoter as identified with the TFBSF tool. Hence it is likely that at least StuA indirectly controls expression of the *anafp* gene via controlling expression of other transcription factors. Some candidate transfactors might already be among the ones listed in S2 and S3 Tables, a hypothesis worth scrutinizing in future experiments. Expression of the *anafp* gene is not only linked to the expression of many secondary metabolites but also with cell wall biosynthesis and remodeling enzymes mainly involved in chitin metabolism (S2 and S3 Tables). As StuA has been shown to also control the expression of cell wall proteins in *A*. *nidulans* [77] and *A*. *chrysogenum* [55], one might assume that a similar StuA controlled interdependence of *anafp* expression with secondary metabolism, cell wall integrity and sporulation might exist in *A*. *niger*. Further experiments are necessary to prove or disprove this hypothesis.

The gene correlation network analysis predicts fifthly, that the function of the *anafp* gene is somehow linked to genes important to sense and withstand osmotic stress (Table 2). These genes included An03g04690 (ortholog of *S*. *cerevisiae* osmosensor Sho1) and An01g06900 (ortholog of *S*. *cerevisiae* stress gene Asg1) to name but a few. The *Panafp*::*luc* reporter assay unambiguously confirmed that the promoter is responsive to the extracellular presence of high salt amounts (Fig 5). It was found in plants that, beside nutrient starvation and oxidative stress, salt stress also induces autophagy, concomitant with upregulation of expression of the autophagy-related gene *AtATG18a* [78, 79] as a consequence of *AtATG1* activation [80]. *AtATG1* is the initiator of autophagy in plants like its ortholog in *S*. *cerevisiae* (Atg1). The respective Atg1 ortholog in *A*. *niger* is An04g03950, which was found to have an expression profile comparable to that of the *anafp* gene during carbon limitation and starvation (see above). Hence, salt stress response in plants involves autophagy which could supposedly also be the case in *A*. *niger*.

## Concluding Remarks—Similar Is Not the Same

Taken together, we could show that the function AnAFP is embedded in several cellular processes some of which are related to self/nonself recognition and the defense against nutrient competitors, some of which are related to nutrient recycling, development and stress survival. A question which imposes itself is, whether AnAFP acts as molecular sensor, as signaling molecule or whether it is just an effector molecule, which intracellularly destabilizes plasma membranes thereby initiating cell lysis. If *A*. *niger* makes most of the protein, it is conceivable that all of these options are true. Given a recursive relationship such as in the immunobiology of higher eukaryotes, AnAFP could be an effector molecule which in turn activates its sensor / signaling molecule in order to amplify a rapid host defense response. For example, expression of the murine β-defensin-2 is induced by a toll-like receptor (TLR4), which reciprocally induces expression of TLR4, thereby stimulating the maturation of dendritic and memory T cells [81]. Hence, expression of the murine β-defensin-2 expands the sensory array to detect microbial signals. Whether such a scenario is also valid for AnAFP and AFPs in general remains to be determined.

AFPs from *Ascomycota* possess a γ-core motif, which was likely inherited from an ancient predecessor molecule designed as chemical weapon to defend its host from enemies. During evolution, AFPs likely adopted additional cellular roles some of which are shared among different AFP-producing fungi and some of which are highly specific only to its host. This might explain why AFPs partly interfere with the other fungi’s sensing and response pathways and why their growth-inhibitory and membrane-permeabilizing effect is restricted to fungi. They specifically interact with structural and dynamic properties of fungal signaling networks and disturb their functionality in related fungi. This interference provokes growth inhibition in the best case, or cell death in the worst case.

## Materials and Methods

### Strains, growth conditions and molecular techniques

All fungal strains used in this study are given in Table 3. *Escherichia (E*.*) coli* strain XL1-blue served as host for all plasmid work. *A*. *niger* strains were cultivated in minimal medium (MM) [82] containing 1% glucose as a carbon source (if not otherwise stated) or in complete medium (CM), consisting of MM supplemented with 1% yeast extract and 0.5% casamino acids. 10 mM uridine was added when required. Fermentation medium (FM) was composed of 0.75% glucose, 0.45% NH_4_Cl, 0.15% KH_2_PO4, 0.05% KCl, 0.05% MgSO_4_, 0.1% salt solution [82] and 0.003% yeast extract. The pH of FM was adjusted to pH 3. Transformation of *A*. *niger*, selection procedures, genomic DNA extraction and diagnostic PCR were performed using recently described protocols [83]. Standard PCR, general cloning procedures in *E*. *coli* and Southern analyses were done according to [84].

**Table 3:** *Aspergillus niger* strains used in this work. CH4.2 and CH4.9 are different transformant strains from the same transformation approach.

Name	Relevant genotype	Reference
N402	*cspA1*	[85]
AB4.1	*cspA1*, *pyrG*^*-*^	[85]
MA169.4	*cspA1*, *kusA*::DR*-amdS-*DR, *pyrG*^*−*^	[86]
MA70.15	*cspA1*, *ΔkusA*, *pyrG*^*-*^, AB4.1 derivative	[87]
MA78.6	*cspA1*,*ΔkusA*, *pyrG*^*+*^, MA70.15 derivative	[86]
CH4.2	*cspA1*, *ΔkusA*, *Δanafp*, *pyrG*^*+*^, MA70.15 derivative	this work
CH4.9	*cspA1*, *ΔkusA*, *Δanafp*, *pyrG*^*+*^, MA70.15 derivative	this work
PK1.22	*cspA1*, *Panafp*::*eyfp*, *Δanafp*, *pyrG*^*+*^, AB4.1 derivative	this work
PK2.9	*cspA1*, *Panafp*::*mluc*, *Δanafp*, *pyrG*^*+*^, AB4.1 derivative	this work
NP2.8	*cspA1*, *Panafp*::*eyfp*, *Δanafp*, *ΔcreA*, *pyrG*^*+*^, *hyg*^*R*^, AB4.1 derivative	this work
NP3.2	*cspA1*, *Panafp*::*eyfp*, *Δanafp*, *ΔflbA*, *pyrG*^*+*^, *hyg*^*R*^, AB4.1 derivative	this work
NP4.1	*cspA1*, *Panafp*::*eyfp*, *Δanafp*, *ΔbrlA*, *pyrG*^*+*^, *hyg*^*R*^, AB4.1 derivative	this work
XY1.1.1	*cspA1*, *creA*::*hygB*, N402 derivative	this work
BN26.1	*cspA1*, *ΔflbA*::*hygB*, N402 derivative	[88]
BN34.2	*cspA1*, *ΔbrlA*::*hygB*, N402 derivative	[88]
NP6.4	*cspA1*, *Panafp*::*mluc*, *Δanafp*, *ΔcreA*, *pyrG*^*+*^, *hyg*^*R*^, AB4.1 derivative	this work

### Preparation of AnAFP

Batch cultivation of *A*. *niger* strain N402 was performed in BioFlo/CelliGen 115 bioreactors (New Brunswick Scientific) as described earlier [29]. AnAFP was purified from *A*. *niger* batch cultures by a method described earlier for the purification of the *A*. *giganteus* AFP [22]. This method is based on cation-exchange chromatography coupled with size-exclusion chromatography and allowed the isolation of about 5 mg pure AnAFP from 1 liter supernatant, which was harvested during the post-exponential growth phase. Purity and concentration of AnAFP was determined by SDS-PAGE [89] as shown in S5 Fig. The identity of the AnAFP was verified using mass spectrometry performed by EUROGENTEC S.A., Belgium.

### Sensitivity tests towards AnAFP

Sensitivity of filamentous fungal strains against AnAFP and AFP was determined using a protocol according to [22]. In brief, 10^3^ spores were used to inoculate 150 μl yeast extract peptone (YPD) medium in the absence or presence of different AnAFP amounts (0.1–50 μg/ml) and AFP amounts (0.1–400 μg/ml). Cultivations were carried out in technical triplicates in microtiter plate format (28°C, 28 h, 120 rpm) and repeated at least twice. Growth was assessed by measuring the optical density at 600 nm.

### SYTOX green membrane permeabilization assay

The assay was carried out in microtiter plate format using a method described recently [21]. In brief, 10^2^ fungal spores of *A*. *niger*, *A*. *nidulans* and *F*. *oxysporum* were cultivated at 28°C in 150 μl YPD medium for 20 h. SYTOX-Green was added to a final concentration of 0.2 μM and AnAFP up to final concentrations of 100 μg/ml. Fluorescence values were measured after 5 min for up to 2 h of incubation using a CytoFluor 2350 fluorescence measurement system (excitation 480 nm, emission 530 nm). All measurements were carried out in triplicates and corrected by subtracting values from AFP-untreated samples.

### Systematic mining of published *A*. *niger* microarray data

As of March 02, 2016, 283 high-throughput microarray data reflecting 155 different cultivation conditions have been made publically available by the scientific community for *A*. *niger* CBS 513.88 and its cognate strains and deposited at the GEO database (http://www.ncbi.nlm.nih.gov/geo/). These data differ with respect to experimental conditions, design and quality, and contain information that were processed and normalized using a wide variety of methods. We thus used the raw expression CEL-files (platform: GPL6758), and performed the microarray preparation and processing of the entire 283 CEL-files in a single step. The different biological conditions for the 283 microarrays as well as their corresponding publications are summarized in S1 Table. CEL-files were processed using the affy package version 1.42.1 from Bioconductor [52] and R version 3.1.0 [90]. RMA was used for normalization of the data [91]. The final *A*. *niger* transcriptomics database was screened for expression levels of the *anafp* ORF An7g01320.

For establishing the gene co-expression network, background correction was done using MAS5 because it better supports network creation then RMA [92]. Data was normalized using the method of invariant sites [93] and the method of Li and Wong [93, 94]. The correlation of *anafp* expression with other genes was assessed by calculating the Spearman's rank correlation coefficient [95]. To assess a threshold of the Spearman coefficient indicating biological relevance, we calculated 100,000 Spearman correlations using arbitrarily generated data sets simulating gene expression. These data sets corresponded to 155 conditions and total values between 0 and 20,000, which is comparable to the real microarray data. Five independent network simulations were calculated. In all calculations the Spearman coefficient did not exceed a value of 0.4. Moreover, less than 0.1% of calculated Spearman coefficients were higher than 0.3. Hence, we concluded that Spearman coefficient values above 0.5 are unlikely to be generated artificially, i.e. only genes with a Spearman's rank correlation coefficient higher than 0.5 were considered for further analysis as these correlations must originate from a biological context.

FetGOat accessible at www.broadinstitute.org/fetgoat/ was used for gene ontology enrichment analyses [20]. *A*. *niger* CBS513.88 served as reference strain. The false discovery rate was set to 0.05. We solely analyzed overrepresented GO terms and used a minimal annotation group size of 2. Genes which were positively or negatively correlated with *anafp* were analyzed separately.

### Transcription factor binding site analysis

*In silico* analysis of putative transcription factor binding sites localized in the 1,000-bp upstream regions of the *anafp* gene was performed using the transcription factor binding site finder (TFBSF) tool [19]. An updated list of known transcription factors from *Aspergillus* or *Trichoderma* species is summarized in S4 Table. To determine significant over- or underrepresentation of binding sites, the background distribution of the identified motifs in the genome of *A*. *niger* was determined via bootstrapping (50,000 bootstraps).

### Identification of AFP orthologs

Protein sequences encoded by *anafp* orthologous genes were aligned using the freely available online program *Multiple Sequence Alignment by ClustalW* at www.genome.jp/tools/clustalw [96]. For calculation the following parameter set was applied: Pairwise alignment was set to slow/accurate for protein; the gap open penalty and gap extension penalty was set to 10 and 0.1, respectively; weight transition was not allowed but hydrophilic gaps; as hydrophilic residues lysine, arginine, serine, proline, glutamate, aspartate, asparagine, glutamine and glycine were selected, BLOSUM for proteins was used as matrix. Synteny of genomic regions around the *anafp* locus in the genomes of the genus *Aspergillus* was analyzed using the Sybil algorithm [97] at AspGD (www.aspgd.com) [15].

### Bioreactor cultivation

Retentostat cultivation of strain PK1.22 (*Panafp*::*eyfp*, *Δanafp*) was performed according to [27]. In brief, continuous cultivation was started in the late exponential growth phase of a batch culture, after 90% of the maltose had been consumed. Carbon-limited fermentation medium, containing 8 g/l of maltose and 0.01% (vol/vol) PPG, was fed to the culture. The flow rate was 0.125 liter×h^-1^, which corresponded to a substrate feed rate of 0.2 g of maltose×h^-^1 kg^-1^. A cell retention device was inserted aseptically to maintain a culture mass of 5 kg. Samples were taken every 24 h and analyzed by differential-interference contrast (DIC) and fluorescence microscopy. Light and fluorescence microscopic pictures were captured with 20× or 40× objectives and using an Axioplan 2 (Zeiss) equipped with a DKC-5000 digital camera (Sony). DIC and YFP settings were used and images were processed using Adobe Photoshop 6.0 (Adobe Systems).

### Construction of deletion and expression cassettes

Standard PCR and cloning procedures were used for the generation of all constructs [84] and all cloned fragments were confirmed by DNA sequencing. Details on cloning protocols and primers used can be requested from the authors. All methods used for transforming *A*. *niger* and selecting positive transformants followed the protocols described in [83]. The *anafp* deletion cassette was made by PCR amplification of the 1000 bp of 5’- and 3’- flanks of ORF An07g01320 and used the Gateway cloning technology for construction. Genomic DNA of *A*. *niger* strain N402 served as template DNA. The sequences were cloned upstream and downstream of the *A*. *oryzae pyrG* selection marker obtained from pAO4-13 [98]. This construct was used to replace the *anafp* ORF with the *pyrG* selection marker in the *A*. *niger* recipient strain MA70.15 (*pyrG*^-^). Successful deletion was verified by Southern analysis and strain CH4.2 was selected for further analysis.

The *creA*, *brlA* and *flbA* genes, respectively, were deleted in strain PK1.22 (see below), and *creA* was also deleted in strain PK2.9 following a similar approach described above but using the split marker technology for improved homologous recombination as described in [99]. The hygromycin resistance cassette from plasmid pAN7-1 was used as selection marker [100]. The designed bipartites were generated by fusion PCR and assembled using Gibson cloning [101]. Positive clones were confirmed via PCR and Southern analysis and the strains NP2.8 (*ΔcreA*, *Panafp*::*eyfp*, *Δanafp*), NP3.2 (Δ*brlA*, *Panafp*::*eyfp*, *Δanafp*), NP4.1 (Δ*flbA*, *Panafp*::*eyfp*, *Δanafp*) and NP6.4 (*Panafp*::*mluc*, *Δanafp*, *ΔcreA*) were selected for further analysis.

*Panafp*::*mluc*::*TtrpC*::*spyrGAo*::*Tanafp* and *Panafp*::*eyfp*::*TtrpC*::*spyrGAo*::*Tanafp* gene replacement constructs were generated using a fusion PCR approach recently described [102] combined with the Gateway cloning technology. Both constructs were transformed in strain AB4.1 (*pyrG*^-^) and successful gene replacement with the *anafp* gene was confirmed by Southern analysis. Strains PK1.22 (*Panafp*::*eyfp*, *Δafp*) and PK2.9 (*Panafp*::*mluc*, *Δafp*) were selected for further analysis.

### Luciferase-based reporter assay in microtiter format

Wells of a 96-well glass bottom MTP were filled with 300 μl of MM, containing 0.003% yeast extract and 1.4 mM luciferin, and were inoculated with 10^5^ cells of strain PK2.9. The plate was incubated for 26 h at 30°C before 30 μl of pre-warmed salt solutions (NaCl, KCl, CaCl_2_, MgCl_2_ and KH_2_PO_4_ dissolved in MM, final concentration per well: 100 mM) or oxidative compounds (menadione or H_2_O_2_, final concentration per well: 100 μM and 5 mM, respectively) were added. OD at 620 nm and luminescence at 490 nm were measured online during the following 10 h at 30°C (VICTOR3^™^, Perkin Elmer).

For the starvation experiments, 20 ml of culture broth samples of strain PK2.9 were taken during the mid-logarithmic growth phase, the biomass washed with sterile water and transferred to 100 ml Erlenmeyer flasks containing 10 ml MM lacking carbon- or nitrogen source, respectively. The flasks were incubated at 30°C and 250 rpm. After 2 h, 6 h and 8 h of incubation, 130 μl of cell cultures were transferred to a 96-well MTP with glass bottom and mixed with 70 μl of a 1.4 mM luciferine solution. OD at 620 nm and luminescence at 490 nm were measured (VICTOR3^™^, Perkin Elmer).

### eYFP-based reporter assay

CM agar plates were covered with sterile cellophane membranes (Bio-Rad) and incubated with 5–10 μl of a spore or cell suspension of the strains PK1.22, NP2.8, NP3.2, NP4.1, N402, BN26.1, BN34.2 or XY1.1, respectively. After 72 h of incubation at 30°C, biomasses were harvested from the plates and freeze-dried (FreeZone 2.5 Liter Benchtop Freeze Dry System, Labconco, USA). The dried biomass was weighted, grinded and resuspended in 50 mM sodium phosphate buffer (pH 7) to a final concentration of 50 μg/ml. After sonication (Bandelin Sonorex TK52 Transistor, Germany) for 10 min at room temperature, cell debris was sedimented at 19100×g for 10 min. Finally, 100 μl of supernatants were transferred to a MTP to measure fluorescence intensity (GloMax^®^-Multi+ Detection System, Promega, Germany) at 510–570 nm after excitation at 490 nm. Background signal of phosphate buffer was subtracted from all sample values. Unfortunately, and in contrast to the *ΔcreA* strains, *ΔflbA* and *ΔbrlA* strains showed an auto-fluorescence signal, which made it impossible to analyze the impact of *anafp* deletion in these strains via fluorescence measurements.

### Phenotypic analyses of *anafp*-deletion strains

Defined spore titres of *A*. *niger* strains were used to inoculated MM or CM and incubated for 1–4 days at 30–37°C. All quantitative measurements (growth rate, germination rate, sporulation efficiency) were performed in triplicates. To determine the amount of spores produced by a colony, all spores formed were carefully taken off with physiological salt solution and a cotton stick and counted using a Thoma chamber. The effect of antifungals was studied as described in [37]. Growth in liquid media was assessed in quadruplet 100 ml CM shake flask cultures inoculated with 10^6^ spores/ml and in the case of non-sporulating *ΔflbA* and *ΔbrlA* inoculated with a defined amount of substrate mycelium obtained from three-day old colonies grown on CM agar. Residual glucose concentrations were measured using the Glucose GOD / PAP liquicolor assay (MTI Diagnostics) following the instructions of the manufacturer.

## Supporting Information

S1 Fig *anafp* expression in *A*. *niger* when exposed to different nitrogen sources. Absolute values of transcription levels are depicted as arbitrary units (AU) of fluorescence intensity. All samples have been taken from stationary growth phase at pH 4 or pH 5, respectively [31]. Numbers in brackets show time points relative to carbon source depletion. Nitrogen was delivered in different concentrations (4x: 282.4 mM; 8x: 564.8 mM) and in form of NH_4_Cl or NaNO_3_, respectively. Glucose (277.5 mM) or xylose (333.0 mM) were used as carbon sources and are indicated.
(TIF)

S2 Fig Influence of carbon- and nitrogen starvation on *anafp* promoter activity using luciferase reporter strain PK2.9. Given are the mean values of two independent experiments using microtiter-based cultivation as a function of starvation length. During the mid-logarithmic growth phase, when supply of all media components is still given to a sufficient extent, samples were taken and transferred to media lacking either a carbon or a nitrogen source. After 2 h, 6 h and 8 h of carbon- and nitrogen starvation conditions, OD_620_ and luminescence (LCPS, luminescence counts per second) were measured in order to quantify *anafp* promoter activity.
(TIF)

S3 Fig *anafp* deletion phenotype. A) Shown are the macroscopic phenotypes of *A*. *niger* strains CH4.2 (*ΔkusA*, *Δanafp*), CH4.9 (*ΔkusA*, *Δanafp*), N402 (wild type) and MA87.6 (*ΔkusA*). Spores of the tested strains were plated on complete (CM) or minimal medium (MM), respectively. All strains were incubated at 37°C for 72 h and in presence or absence of 0.1 mg/ml calcofluor white (CFW), 1.2 M sorbitol or 1 M NaCl, respectively. B) Shown are the macroscopic phenotypes of *A*. *niger* strains N402, PK1.22, XY1.1, NP2.8, BN26.1, NP3.2, BN34.2 and NP4.1. Genes which were deleted in the corresponding strains are indicated on top and on the left of the photographs, respectively. wt, wild type. C) Determined final biomass (left panel) and pH values (right panel) from shake flask cultures incubated for 11 days. At the last four days the cultures were completely free of glucose, indicating a severe carbon starvation milieu. In brackets, corresponding genotypes of the tested strains are indicated. Depicted are the mean values of two independent experiments each performed as duplicate approach.
(TIF)

S4 Fig Gene ontology (GO) enrichment analysis using the FetGOat tool [20]. Depicted are the enriched A) biological processes (GO:0008150) and B) molecular functions (GO:0003674) which are positively correlated with *anafp* expression, whereas C), D) and E) summarize the biological processes (GO:0008150), cellular components (GO:0005575) and molecular functions (GO:0003674), respectively, which are negatively related to *anafp* expression.
(PDF)

S5 Fig Purity of AnAFP sample isolated from *A*. *niger* wild type strain N402. Shown is the result of an SDS-PAGE gel analysis using Read Gel precast Tris-HCl gradient (4–15%) gels from Bio-Rad. A) Protein molecular standard in kDa. B) Purified AnAFP sample, which shows only one protein band in the expected molecular weight range of 4–6 kDa. Theoretical molecular weight of AnAFP amounts to 6.5 kDa.
(TIF)

S1 Table Survey of the 155 different biological conditions and expression levels of the 283 microarrays as well as their corresponding publications considered for transcriptome meta-analysis. In case of replicate assays, expression levels are shown as average value with corresponding standard deviations (SD). AU, arbitrary units of fluorescence signal. *GSE numbers were provided if made available by the authors of corresponding publications.
(PDF)

S2 Table Survey of genes positively correlated with *anafp* gene sorted by Spearman coefficient. Pairwise correlation coefficients (Spearman coefficient) for the expression profiles of all ~ 14.000 *A*. *niger* genes under the 155 different cultivations present in our in-house transcriptomic database were calculated using Bioconductor [52]. Pairs with a Spearman score ≥ 0.5 were considered to be significantly co-expressed.
(PDF)

S3 Table Survey of genes negatively correlated with *anafp* gene sorted by Spearman coefficient. Pairwise correlation coefficients (Spearman coefficient) for the expression profiles of all ~ 14.000 *A*. *niger* genes under the 155 different cultivations present in our in-house transcriptomic database were calculated using Bioconductor [52]. Pairs with a Spearman score ≥ 0.5 were considered to be significantly co-expressed.
(PDF)

S4 Table List of transcription factors (TF) of *Aspergillus* and *Trichoderma* and their known promoter binding sites used for screening with the TFBSF tool [19]. (PDF)

## Abbreviations

MIC: minimal inhibitory concentration
AFP: antifungal protein
AnAFP: *A*. *niger* antifungal protein
CA: caspofungin
FP: fenpropimorph
AU: arbitrary units
LCPS: luminescence count per second
OD: optical density
eYFP: enhanced yellow fluorescent protein
PAF: *Penicillium chrysogenum* antifungal protein
ORF: open reading frame
