# Supplementary figures and images for: A Transcriptome Meta-Analysis Proposes Novel Biological Roles for the Antifungal Protein AnAFP in Aspergillus niger
Source: PLoS One. 2016 Nov 11;11(11):e0165755. doi: 10.1371/journal.pone.0165755 (PMC5106034; doi:10.1371/journal.pone.0165755)

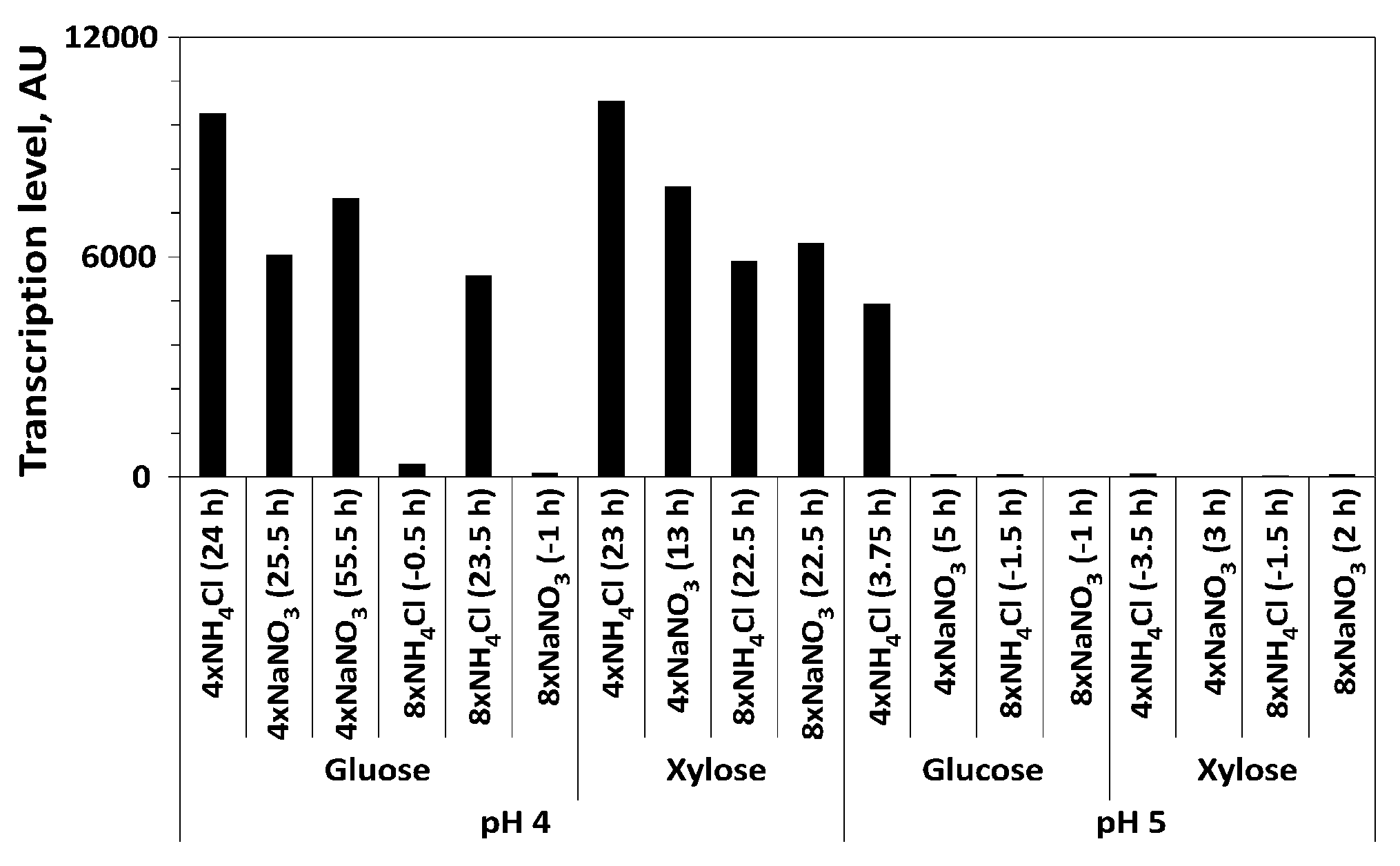

Supplement: S1 Fig — Absolute values of transcription levels are depicted as arbitrary units (AU) of fluorescence intensity. All samples have been taken from stationary growth phase at pH 4 or pH 5, respectively [31]. Numbers in brackets show time points relative to carbon source depletion. Nitrogen was delivered in different concentrations (4x: 282.4 mM; 8x: 564.8 mM) and in form of NH4Cl or NaNO3, respectively. Glucose (277.5 mM) or xylose (333.0 mM) were used as carbon sources and are indicated. (TIF) [file pone.0165755.s001.tif]

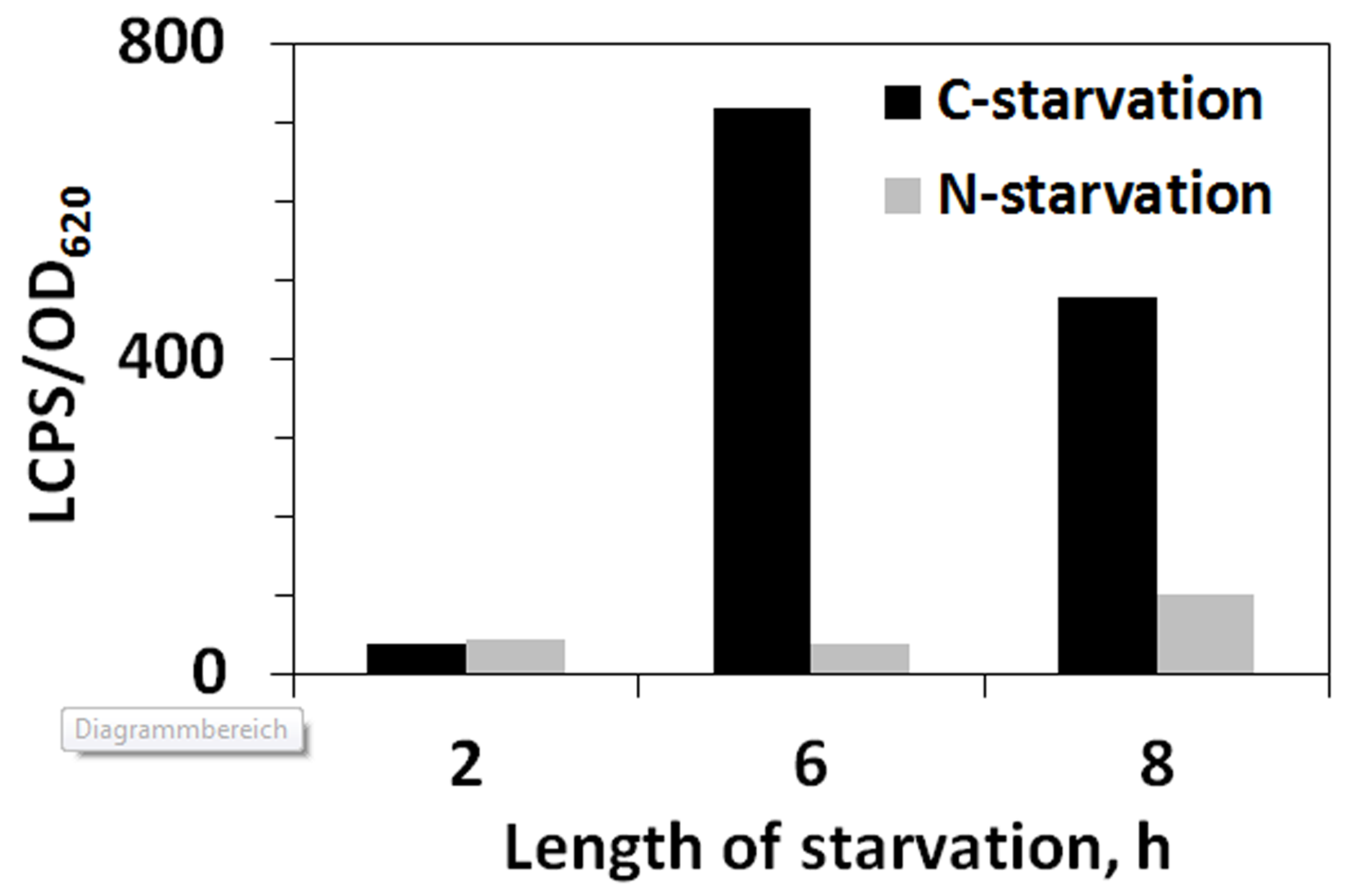

Supplement: S2 Fig — Given are the mean values of two independent experiments using microtiter-based cultivation as a function of starvation length. During the mid-logarithmic growth phase, when supply of all media components is still given to a sufficient extent, samples were taken and transferred to media lacking either a carbon or a nitrogen source. After 2 h, 6 h and 8 h of carbon- and nitrogen starvation conditions, OD620 and luminescence (LCPS, luminescence counts per second) were measured in order to quantify anafp promoter activity. (TIF) [file pone.0165755.s002.tif]

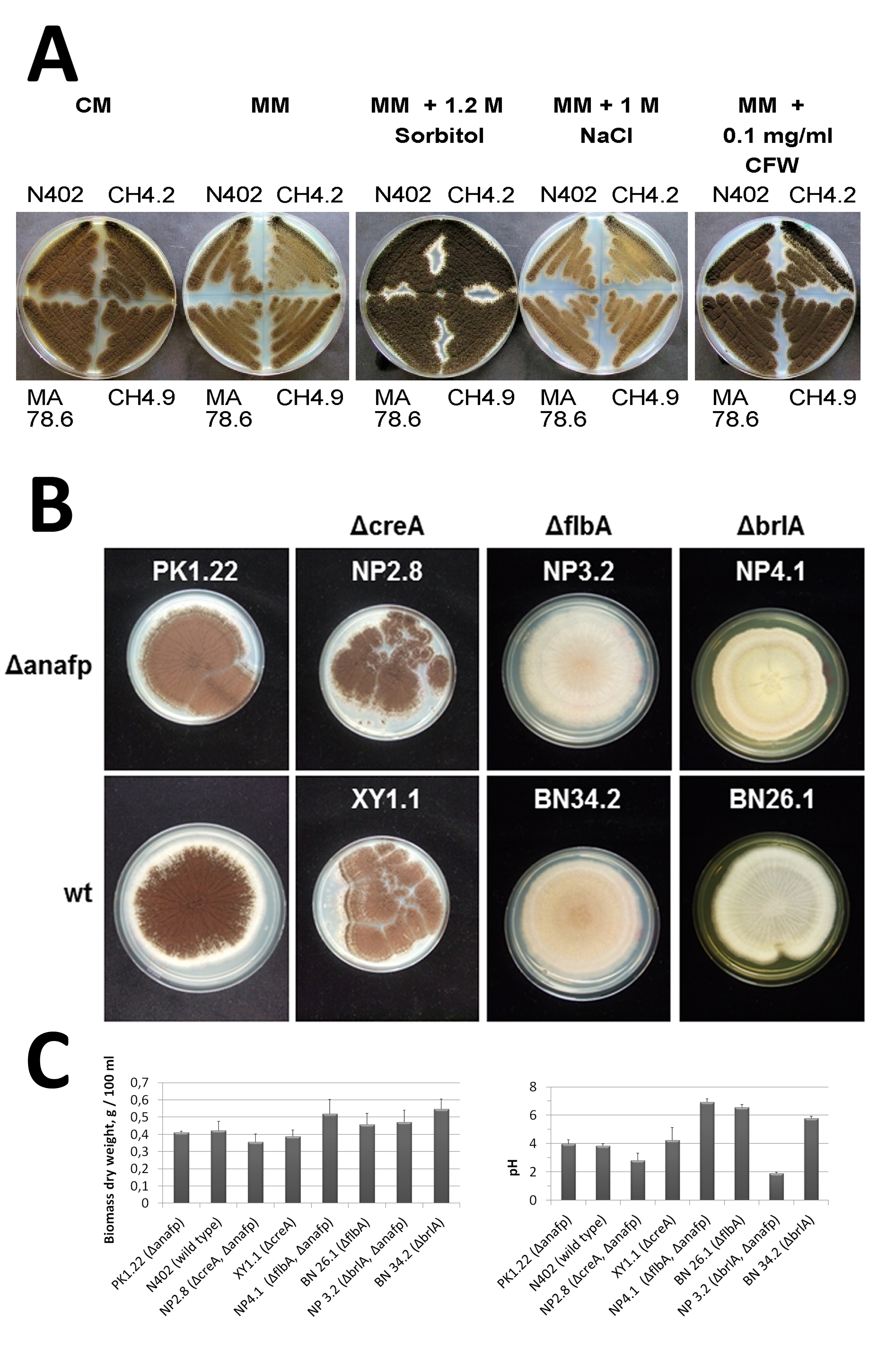

Supplement: S3 Fig — A) Shown are the macroscopic phenotypes of A. niger strains CH4.2 (ΔkusA, Δanafp), CH4.9 (ΔkusA, Δanafp), N402 (wild type) and MA87.6 (ΔkusA). Spores of the tested strains were plated on complete (CM) or minimal medium (MM), respectively. All strains were incubated at 37°C for 72 h and in presence or absence of 0.1 mg/ml calcofluor white (CFW), 1.2 M sorbitol or 1 M NaCl, respectively. B) Shown are the macroscopic phenotypes of A. niger strains N402, PK1.22, XY1.1, NP2.8, BN26.1, NP3.2, BN34.2 and NP4.1. Genes which were deleted in the corresponding strains are indicated on top and on the left of the photographs, respectively. wt, wild type. C) Determined final biomass (left panel) and pH values (right panel) from shake flask cultures incubated for 11 days. At the last four days the cultures were completely free of glucose, indicating a severe carbon starvation milieu. In brackets, corresponding genotypes of the tested strains are indicated. Depicted are the mean values of two independent experiments each performed as duplicate approach. (TIF) [file pone.0165755.s003.tif]

S4A Fig

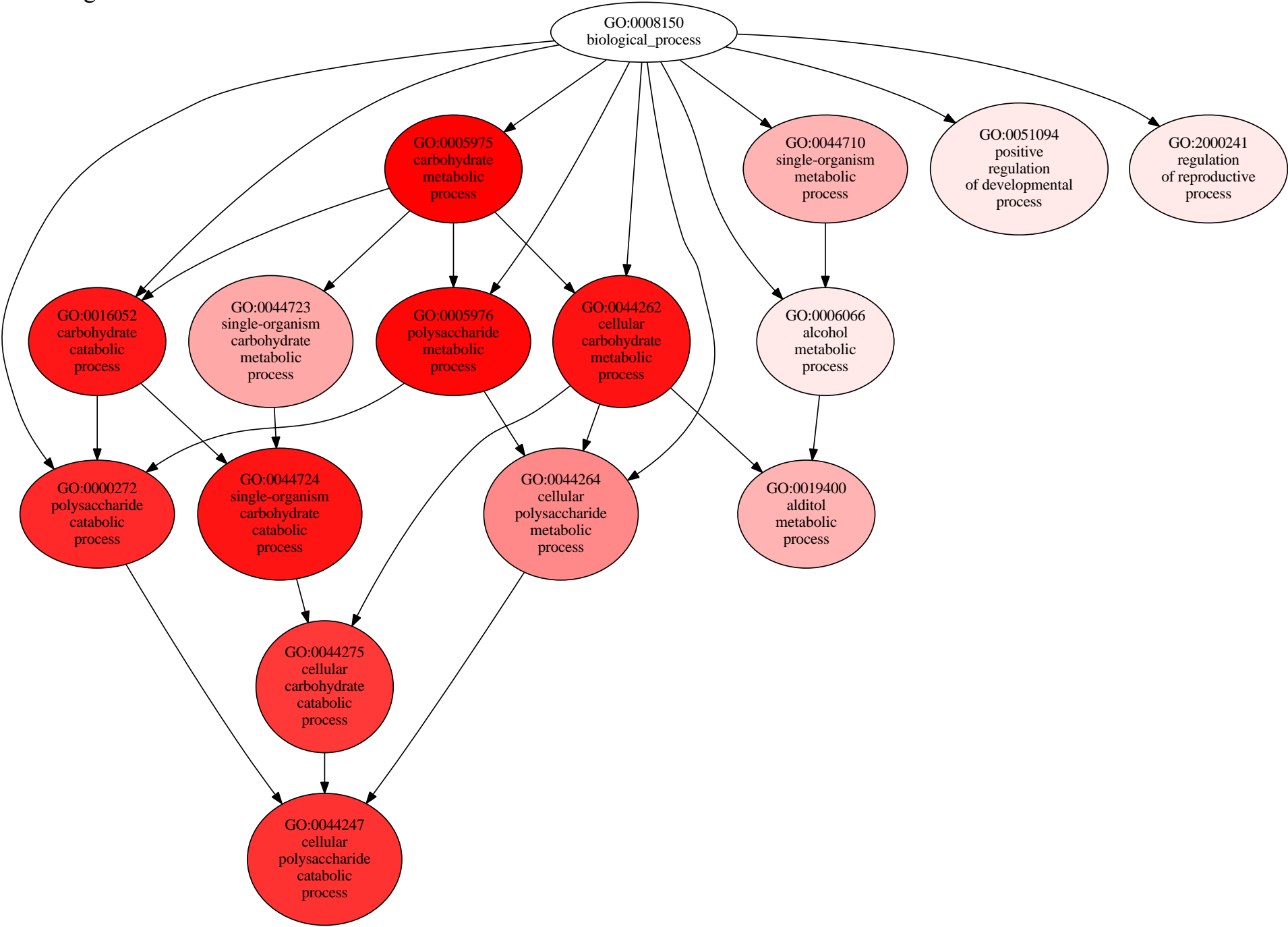

S4B Fig

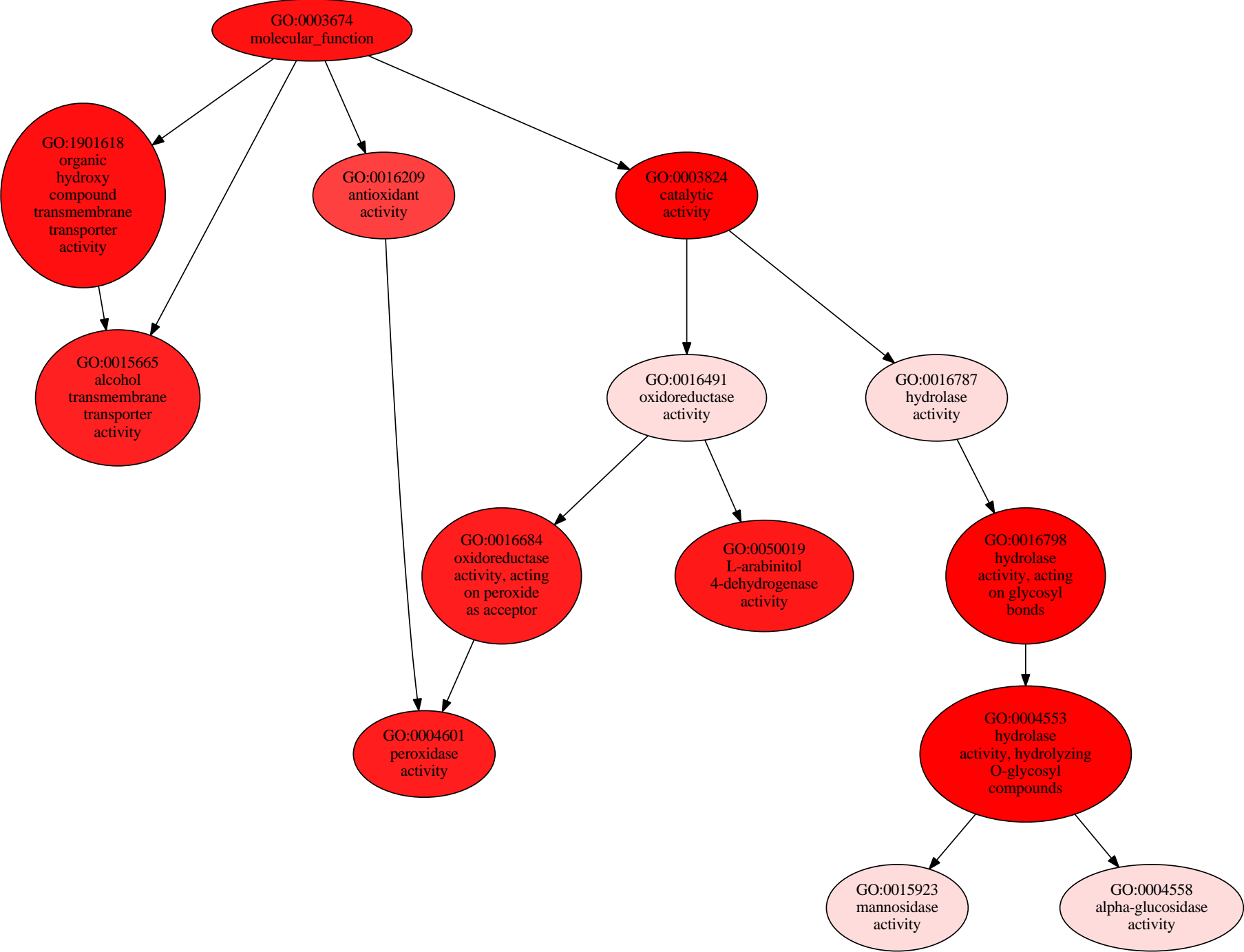

[illegible]

S4D Fig

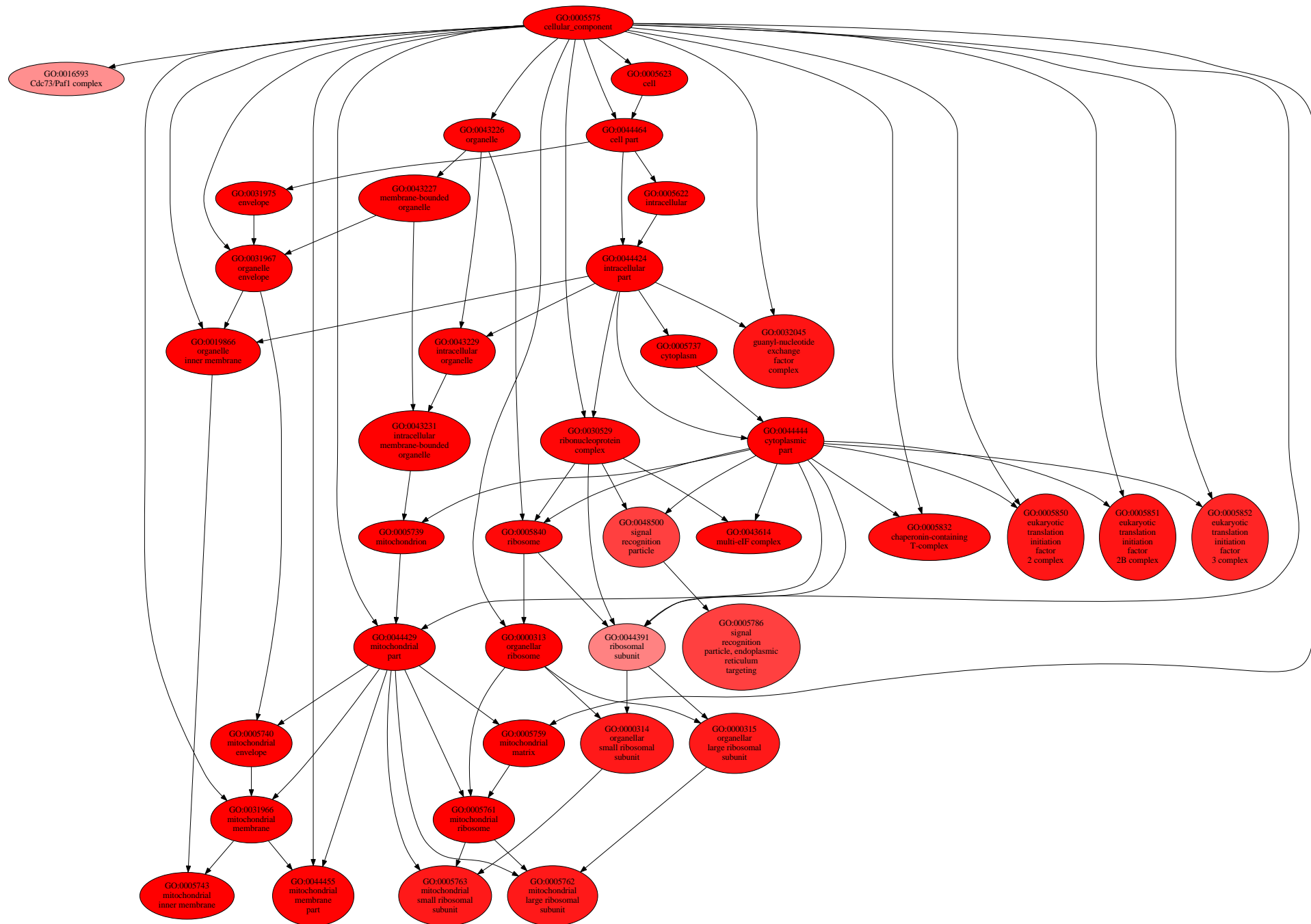

S4E Fig

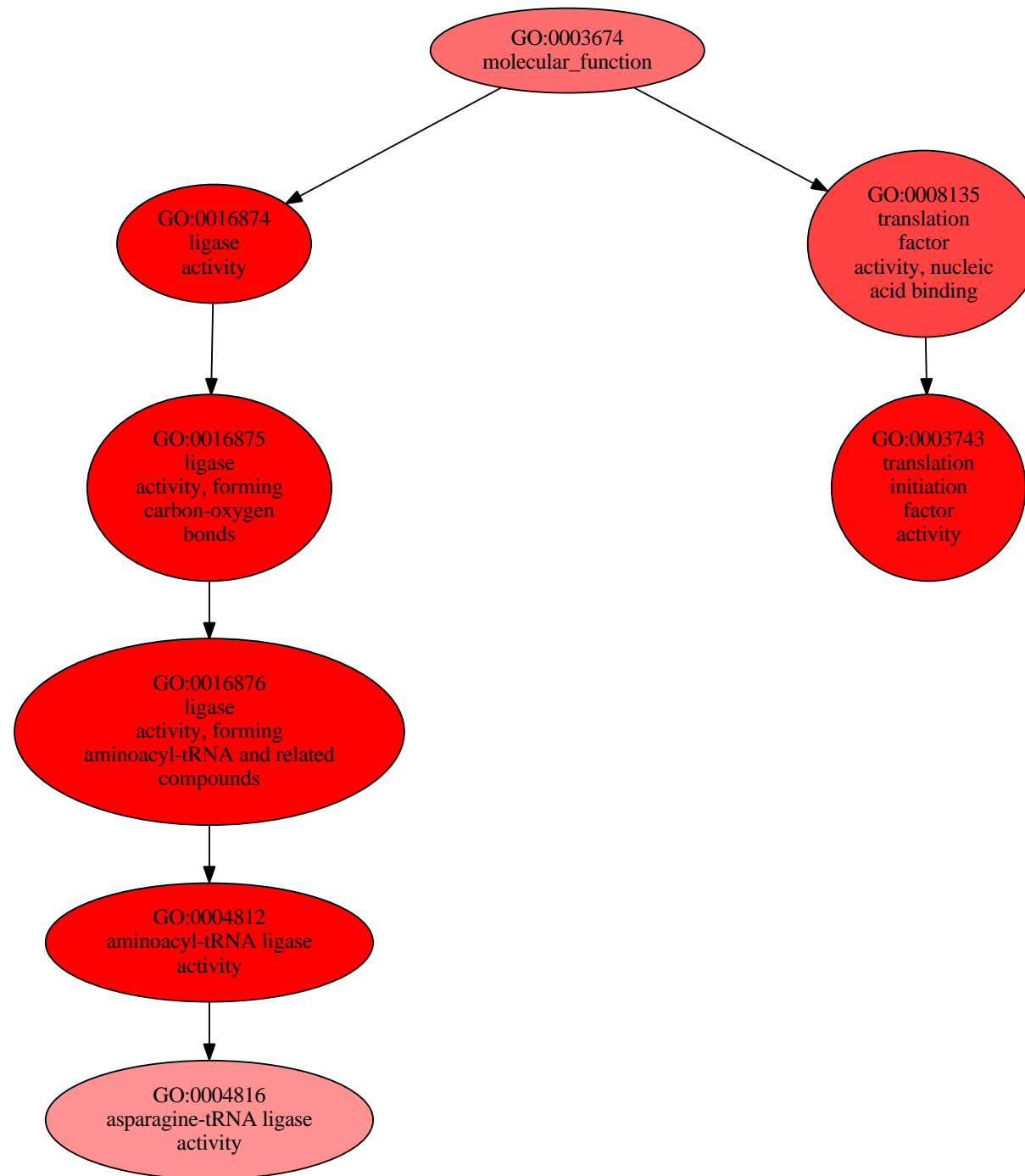

Supplement: S4 Fig — Depicted are the enriched A) biological processes (GO:0008150) and B) molecular functions (GO:0003674) which are positively correlated with anafp expression, whereas C), D) and E) summarize the biological processes (GO:0008150), cellular components (GO:0005575) and molecular functions (GO:0003674), respectively, which are negatively related to anafp expression. (PDF) [file pone.0165755.s004.pdf]

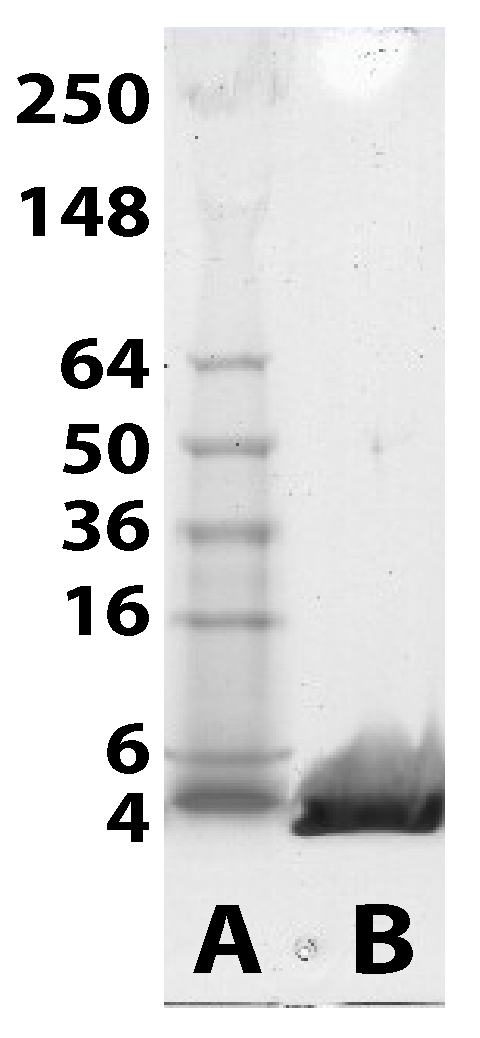

Supplement: S5 Fig — Shown is the result of an SDS-PAGE gel analysis using Read Gel precast Tris-HCl gradient (4–15%) gels from Bio-Rad. A) Protein molecular standard in kDa. B) Purified AnAFP sample, which shows only one protein band in the expected molecular weight range of 4–6 kDa. Theoretical molecular weight of AnAFP amounts to 6.5 kDa. (TIF) [file pone.0165755.s005.tif]
