# Supplementary material for: A Transcriptome Meta-Analysis Proposes Novel Biological Roles for the Antifungal Protein AnAFP in Aspergillus niger
Source: PLoS One. 2016 Nov 11;11(11):e0165755. doi: 10.1371/journal.pone.0165755 (PMC5106034; doi:10.1371/journal.pone.0165755)
Supplement: S1 Table — In case of replicate assays, expression levels are shown as average value with corresponding standard deviations (SD). AU, arbitrary units of fluorescence signal. *GSE numbers were provided if made available by the authors of corresponding publications. (PDF) [file pone.0165755.s006.pdf]

S1 Table

ID condition	ID chip	GEO series*	Expression level, AU	SD Expression level	Publication / Remark
1	1	GSE42258	53,7	17,9	Kwon MJ, et al. The transcriptomic signature of RacA activation and inactivation provides new insights into the morphogenetic network of Aspergillus niger. PLoS One 2013;8(7):e68946.
	2	GSE42258			
	3	GSE42258			
2	4	GSE42258	611,0	118,5	
	5	GSE42258			
3	6	GSE42258	386,5	5,7	
	7	GSE42258			
4	8	GSE42258	481,5	47,1	
	9	GSE42258			
5	10	GSE42258	1219,8	786,8	
	11	GSE42258			
6	12	GSE42258	29,2	0,5	
	13	GSE42258			
	14	GSE42258			
7	15		42,6	18,9	Addtional own unplished data to Kwon MJ, et al. 2013
	16				
8	17		29,9	5,9	
	18				
9	19		30,1	0,3	
	20				
10	21	GSE17641	26,2	5,5	Meyer V, et al. Reconstruction of signaling networks regulating fungal morphogenesis by transcriptomics. Eukaryot Cell 2009 Nov;8(11):1677-91.
	22	GSE17641			
	23	GSE17641			
11	24	GSE17641	27,0	0,9	
	25	GSE17641			
	26	GSE17641			
12	27	GSE56471	27,0	3,8	Fiedler M, et al. The capacity of Aspergillus niger to sense and respond to cell wall stress requires at least three transcription factors: RlmA, MsnA AND CrzA. Fungal Biol Biotechnol 2014, 1(5).
	28	GSE56471			
13	29	GSE56471	26,7	3,6	
	30	GSE56471			
14	31	GSE56471	25,1	0,7	
	32	GSE56471			
15	33	GSE56471	27,2	3,7	
	34	GSE56471			
16	35	GSE56471	26,8	0,3	
	36	GSE56471			
17	37	GSE56471	24,3	1,6	
	38	GSE56471			
18	39	GSE56471	26,4	2,2	
	40	GSE56471			
19	41	GSE56471	26,2	9,7	
	42	GSE56471			
20	43		26,7	1,1	Addtional own unplished data to Fiedler M, et al. 2014; testing of AFP and caspofungin
	44				
21	45		31,7	5,5	
	46				
22	47		257,2	96,3	Jørgensen TR, et al. Transcriptomic comparison of Aspergillus niger growing on two different sugars reveals coordinated regulation of the secretory pathway. BMC Genomics 2009, 10:44.
	48				
	49				
23	50		209,8	80,2	
	51				
	52				
24	53		194,3	56,9	
	54				
	55				
25	56		138,4	18,4	
	57				
	58				
26	59		24,4	1,4	Yuan XL, et al. Identification of InuR, a new Zn(II)2Cys6 transcriptional activator involved in the regulation of inulinolytic genes in Aspergillus niger. Mol Genet Genomics. 2008 Jan;279(1):11-26.
	60				
27	61		25,9	0,6	
	62				
28	63		24,4	1,4	
	64				
29	65		28,2	3,8	
	66				
30	67		30,0	5,1	
	68				
31	69		27,1	2,0	
	70				
32	71		50,9	26,1	
	72				
33	73		29,5	0,7	Guillemette T, et al. Genomic analysis of the secretion stress response in the enzyme-producing cell factory Aspergillus niger. BMC Genomics 2007
	74				

34	75		31,1	1,7	the enzyme-producing cell factory Aspergillus niger. BMC Genomics 2007; 8:158.
	76				
35	77	GSE39559	4013,1	291,0	Nitsche BM, et al. The carbon starvation response of Aspergillus niger during submerged cultivation: insights from the transcriptome and secretome. BMC Genomics 2012 Aug 8;13:380.
	78	GSE39559			
36	79	GSE39559	2854,1	195,4	
	80	GSE39559			
37	81	GSE39559	912,4	89,2	
	82	GSE39559			
38	83		30,1	2,6	van Münster JM, et al. Systems approaches to predict the functions of glycoside hydrolases during the life cycle of Aspergillus niger using developmental mutants ΔbrlA and ΔflbA. PLoS One. 2015 Jan 28;10(1):e0116269.
	84				
39	85		16956,5	762,1	
	86				
40	87		21320,8	152,2	
	88				
41	89		20195,6	1007,9	
	90				
42	91		752,4	15,4	
	92				
43	93		660,5	171,5	
	94				
44	95		5601,0	393,2	
	96				
45	97		2571,6	443,1	
	98				
46	99	GSE21752	15300,4	1362,4	Jørgensen TR, et al. Transcriptomic insights into the physiology of Aspergillus niger approaching a specific growth rate of zero. Appl Environ Microbiol 2010 Aug;76(16):5344-55.
	100	GSE21752			
	101	GSE21752			
47	102	GSE21752	16471,3	2222,5	
	103	GSE21752			
	104	GSE21752			
48	105		28,2	0,9	Own unpublished data; Secretion stress response to tunicamycin
	106				
	107				
49	108		28,3	2,0	
	109				
	110				
50	111		24,5	2,1	
	112				
	113				
51	114		22,8	5,0	
	115				
	116				
52	117	GSE39070	35,6	6,8	Carvalho ND, et al. Genome-wide expression analysis upon constitutive activation of the HacA bZIP transcription factor in Aspergillus niger reveals a coordinated cellular response to counteract ER stress. BMC Genomics 2012 Jul 30;13:350.
	118	GSE39070			
	119	GSE39070			
53	120	GSE39070	28,5	0,3	
	121	GSE39070			
	122	GSE39070			
54	123	GSE39070	27,1	1,0	
	124	GSE39070			
	125	GSE39070			
55	126	GSE39070	26,6	0,5	
	127	GSE39070			
	128	GSE39070			
56	129	GSE14285	31,7		van den Berg RA, et al. Identification of modules in Aspergillus niger by gene co-expression network analysis. Fungal Genet Biol 2010 Jun;47(6):539-50.
57	130	GSE14285	31,0		
58	131	GSE14285	29,7		
59	132	GSE14285	27,0		
60	133	GSE14285	38,2		
61	134	GSE14285	38,0		
62	135	GSE14285	38,8		
63	136	GSE14285	50,4		
64	137	GSE14285	34,9		
65	138	GSE14285	33,3		
66	139	GSE14285	32,4		
67	140	GSE14285	31,6		
68	141	GSE14285	36,3		
69	142	GSE14285	33,1		
70	143	GSE14285	29,5		
71	144	GSE14285	29,4		
72	145	GSE11405	30,8	1,5	van der Veen D, et al. Analysis of variance components reveals the contribution of sample processing to transcript variation. Appl Environ Microbiol 2009 Apr;75(8):2414-22.
	146	GSE11405			
	147	GSE11405			
	148	GSE11405			
	149	GSE11405			
73	150	GSE11405	31,8	0,9	
	151	GSE11405			

74	152		30,8		Martens-Uzunova ES, Schaap PJ. An evolutionary conserved d-galacturonic acid metabolic pathway operates across filamentous fungi capable of pectin degradation. Fungal Genet Biol 2008, 45(11):1449-1457.
75	153		36,7		
76	154		302,9		
77	155		3904,7		
78	156		144,7		
79	157		48,3		
80	158		29,2		
81	159		62,8		
82	160		79,7		
83	161		28,0		
84	162		30,5		
85	163		28,6		
86	164		32,8		
87	165		29,2		
88	166		34,1		
89	167		42,4		
90	168		3217,3		
91	169		61,2		
92	170		202,1		
93	171		164,5		
94	172		3518,0		
95	173		28,4		
96	174		25,9		
97	175		27,5		
98	176		531,0		
99	177		30,2		
100	178		32,0		
101	179		27,3		
102	180		5963,0		
103	181	GSE17329	87,4		van den Berg RA, et al. Identification of modules in Aspergillus niger by gene co-expression network analysis. Fungal Genet Biol 2010 Jun;47(6):539-50.
104	182	GSE17329	27,4		
105	183	GSE17329	6391,4		
106	184	GSE17329	120,5		
107	185	GSE17329	7930,3		
108	186	GSE17329	25,1		
109	187	GSE17329	97,1		
110	188	GSE17329	6077,7		
111	189	GSE17329	7610,8		
112	190	GSE17329	4735,2		
113	191	GSE17329	9921,1		
114	192	GSE17329	70,6		
115	193	GSE17329	10266,5		
116	194	GSE17329	5895,2		
117	195	GSE17329	354,3	20,0	
	196	GSE17329			
118	197	GSE17329	5512,7	336,7	
	198	GSE17329			
119	199	GSE17329	79,8		
120	200	GSE17329	44,0		
121	201		3051,0	409,7	Kwon MJ, et al. The transcriptomic fingerprint of glucoamylase over-expression in Aspergillus niger. BMC Genomics. 2012 Dec 13;13:701.
	202				
	203				
122	204		761,3	28,5	
	205				
	206				
123	207		1520,6	364,1	Ram et al., unplished data; SCLA mutant analysis
	208				
124	209		741,6	116,3	
	210				
125	211	GSE50523	27,5	1,4	Schachtschabel D, et al. The transcriptional repressor TupA in Aspergillus niger is involved in controlling gene expression related to cell wall biosynthesis, development, and nitrogen source availability. PLoS One 2013;8(10):e78102.
	212	GSE50523			
126	213	GSE50523	35,5	9,2	
	214	GSE50523			
127	215	GSE25497	95,4	31,7	de Bekker C, et al. Single tip transcriptomics of neighboring hyphae of Aspergillus niger. Genome Biol 2011 Aug 4;12(8):R71.
	216	GSE25497			
	217	GSE25497			
	218	GSE25497			
	219	GSE25497			
128	220	GSE32123	1944,1	37,4	Bleichrodt R, et al. Cytosolic streaming in vegetative mycelium and aerial structures of Aspergillus niger. Stud Mycol 2013 Mar 15;74(1):31-46.
	221	GSE32123			
	222	GSE32123			
129	223	GSE32123	7023,9	1206,9	
	224	GSE32123			
	225	GSE32123			
130	226	GSE36439	32,3	1,1	van Leeuwen MR, et al. Germination of conidia of Aspergillus niger is
	227	GSE36439			
	228	GSE36439			
131	229	GSE36439	30,4	2,4	
	230	GSE36439			
	231	GSE36439			
132	232	GSE36439	31,5	4,4	

	233	GSE36439			accompanied by major changes in RNA profiles. Stud Mycol 2013 Mar 15;74(1):59-70
	234	GSE36439			
133	235	GSE36439	29,3	0,7	
	236	GSE36439			
	237	GSE36439			
134	238	GSE36439	28,0	3,9	
	239	GSE36439			
	240	GSE36439			
135	241	GSE36440	32,6	1,9	van Leeuwen MR, et al. The effect of natamycin on the transcriptome of conidia of Aspergillus niger. Stud Mycol 2013 Mar 15;74(1):71-85
	242	GSE36440			
	243	GSE36440			
136	244	GSE36440	27,3	1,8	
	245	GSE36440			
	246	GSE36440			
137	247	GSE36440	34,8	1,7	
	248	GSE36440			
	249	GSE36440			
138	250	GSE36440	26,7	0,7	
	251	GSE36440			
	252	GSE36440			
139	253	GSE40219	722,9	585,8	Gruben BS, et al. GalX regulates the D-galactose oxido-reductive pathway in Aspergillus niger. FEBS Lett 2012 Nov 16;586(22):3980-5.
	254	GSE40219			
140	255	GSE40219	259,3	208,8	
	256	GSE40219			
141	257	GSE42480	34,0		Novodvorska M, et al. Trancriptional landscape of Aspergillus niger at breaking of conidial dormancy revealed by RNA-sequencing. BMC Genomics 2013 Apr 11;14:246.
142	258	GSE42480	35,5		
143	259	GSE42480	26,8		
144	260	GSE42480	23,8		
145	261	GSE42480	26,9		
146	262	GSE44391	13659,2	8655,3	Krijgsheld P, Wosten HAB. Transcriptome Analysis of Zones of Colonies of the flbA strain of Aspergillus niger. Fungal Genom Biol 3:109. doi: 10.4172/2165-8056.1000109
	263	GSE44391			
147	264	GSE44391	9540,5	8403,3	
	265	GSE44391			
148	266	GSE44391	3488,1	3407,0	
	267	GSE44391			
149	268	GSE44391	14092,3	582,1	
	269	GSE44391			
150	270	GSE44391	15759,2	1735,0	
	271	GSE44391			
151	272	GSE44391	7312,6	920,7	
	273	GSE44391			
152	274	GSE51023	1447,1	353,7	Gruben BS, et al. Aspergillus niger RhaR, a regulator involved in L-rhamnose release and catabolism. Appl Microbiol Biotechnol 2014 Jun;98(12):5531-40.
	275	GSE51023			
153	276	GSE51023	536,9	58,0	
	277	GSE51023			
154	278	GSE46187	22,0	1,2	Benoit I, et al. Bacillus subtilis attachment to Aspergillus niger hyphae results in mutually altered metabolism. Environ Microbiol 2015 Jun;17(6):2099-113.
	279	GSE46187			
	280	GSE46187			
155	281	GSE46187	22,1	4,6	
	282	GSE46187			
	283	GSE46187			

*If made available by the authors
