# Supplementary material for: A Transcriptome Meta-Analysis Proposes Novel Biological Roles for the Antifungal Protein AnAFP in Aspergillus niger
Source: PLoS One. 2016 Nov 11;11(11):e0165755. doi: 10.1371/journal.pone.0165755 (PMC5106034; doi:10.1371/journal.pone.0165755)
Supplement: S2 Table — Pairwise correlation coefficients (Spearman coefficient) for the expression profiles of all ~ 14.000 A. niger genes under the 155 different cultivations present in our in-house transcriptomic database were calculated using Bioconductor [52]. Pairs with a Spearman score ≥ 0.5 were considered to be significantly co-expressed. (PDF) [file pone.0165755.s007.pdf]

S2 Table

Gene ID	Annotation	Spearman coefficient
An12g01300	similarity to vegetative incompatibility protein tol - Neurospora crassa	0.75
An18g00980	weak similarity to integral membrane protein PTH11 - Magnaporthe grisea	0.7
An18g00200	hypothetical protein	0.7
An13g01870	similarity to hypothetical protein encoded by An04g06980 - Aspergillus niger	0.7
An13g01860	strong similarity to GABA permease gabA - Aspergillus nidulans	0.7
An12g09970	similarity to hypothetical protein encoded by An12g02180 - Aspergillus niger	0.7
An12g09270	strong similarity to lactose permease Lac12 - Kluyveromyces lactis	0.7
An12g05370	strong similarity to O-methyltransferase A omtA - Aspergillus parasiticus	0.7
An09g01810	strong similarity to ketoreductase from patent EP918090-A - Saccharomyces cerevisiae	0.7
An09g00960	hypothetical protein	0.7
An08g00850	weak similarity to hypothetical protein encoded by An13g00450 - Aspergillus niger	0.7
An07g05460	strong similarity to hypothetical protein CAE47856.1 - Aspergillus fumigatus	0.7
An07g03260	similarity to hypothetical protein B15I20.50 - Neurospora crassa	0.7
An06g00170	alpha-galactosidase aglA - Aspergillus niger	0.7
An02g11180	strong similarity to EST an_0208 - Aspergillus niger	0.7
An02g03190	similarity to monocarboxylate transporter MCT3 - Rattus norvegicus	0.7
An02g01030	hypothetical protein	0.7
An01g14000	strong similarity to cyclohexanol dehydrogenase chnA - Acinetobacter sp.	0.7
An17g01450	strong similarity to high-affinity nicotinic acid permease Tna1 - Saccharomyces cerevisiae	0.65
An16g07260	weak similarity to epithelial microtubule-associated protein E-MAP-115 - Homo sapiens	0.65
An16g06270	similarity to versicolorin reductase verA - Aspergillus nidulans	0.65
An16g05180	hypothetical protein	0.65
An16g01600	similarity to monosaccharide transporter 1 pmt1 - Petunia hybrida	0.65
An15g06200	similarity to transcriptional regulator molecule HTRM clone 693452 from patent WO9957144-A2 - Homo sapiens	0.65
An15g02270	hypothetical protein	0.65
An15g02140	weak similarity to dihydrofolate reductase dfr1p - Schizosaccharomyces pombe	0.65
An15g02130	strong similarity to lovastatin diketide synthase lovF - Aspergillus terreus	0.65
An15g01900	strong similarity to choline permease Hnm1 - Saccharomyces cerevisiae	0.65
An14g03850	strong similarity to cytochrome P450 monooxygenase TRI4 - Fusarium sporotrichioides	0.65
An14g02100	strong similarity to cell wall antigen MP1 - Penicillium marneffei	0.65
An14g01370	hypothetical protein	0.65
An14g00600	hypothetical protein	0.65
An13g02730	strong similarity to EST an_3461 - Aspergillus niger	0.65
An13g00630	similarity to protein CAP5 - Colletotrichum gloeosporioides	0.65
An12g10830	similarity to hypothetical protein EAA74834.1 - Gibberella zeae	0.65
An12g05380	strong similarity to cercosporin transporter CFP - Cercospora kikuchii	0.65
An12g02900	similarity to heterokaryon incompatibility protein het-6 - Neurospora crassa	0.65
An12g01820	similarity to ubiquitin thiolesterase L3 - Homo sapiens	0.65
An12g00710	strong similarity to hypothetical protein CAD21276.1 - Neurospora crassa	0.65
An11g07040	strong similarity to EST an_2779 - Aspergillus niger	0.65
An11g06430	similarity to integral membrane protein PTH11 - Magnaporthe grisea	0.65
An11g02980	strong similarity to acetamidase amdS - Aspergillus oryzae	0.65
An11g02950	similarity to calpain nCL-3 from patent DE19650142-A1 - Mus musculus	0.65
An10g00560	strong similarity to hypothetical protein binA - Aspergillus nidulans	0.65
An09g03010	strong similarity to EST an_2241 - Aspergillus niger	0.65
An09g02240	strong similarity to beta-N-acetylhexosaminidase from patent WO9839459-A1 - Penicillium chrysogenum	0.65
An09g02180	strong similarity to phospholipase A1 from patent JP10155493-A - Aspergillus oryzae	0.65

An09g01880	strong similarity to enoyl reductase of the lovastatin biosynthesis lovC - Aspergillus terreus	0.65
An09g00160	similarity to hypothetical protein Rv0839 - Mycobacterium tuberculosis	0.65
An08g10010	hypothetical protein	0.65
An08g10000	strong similarity to cytochrome b245 beta chain CYBB - Homo sapiens [putative frameshift]	0.65
An08g08510	strong similarity to aldehyde dehydrogenase aldh3 - Homo sapiens	0.65
An08g08480	strong similarity to NADPH-dependent carbonyl reductase S1 - Candida magnoliae	0.65
An08g07920	similarity to phenol 2-monooxygenase - Trichosporon beigelii	0.65
An08g00170	hypothetical protein	0.65
An07g07370	weak similarity to PC-MYB2 - Arabidopsis thaliana	0.65
An07g04620	strong similarity to hypothetical protein EAA60744.1 - Aspergillus nidulans	0.65
An07g04500	hypothetical protein	0.65
An07g04350	hypothetical protein	0.65
An07g04330	similarity to heterokaryon incompatibility protein het-6 - Neurospora crassa	0.65
An07g03030	strong similarity to EST SEQ ID NO:4127 from patent WO200056762-A2 - Aspergillus niger	0.65
An07g00540	similarity to hypothetical nucleoside-diphosphate-sugar epimerase AAM31397.1 - Methanosarcina mazei	0.65
An06g02120	weak similarity to Tyk2 non-receptor tyrosine - Mus musculus	0.65
An06g02110	similarity to protein nompC - Drosophila melanogaster	0.65
An06g00920	hypothetical protein	0.65
An06g00620	strong similarity to alpha-glucoside-hydrogen symporter Mal11 - Saccharomyces cerevisiae	0.65
An06g00260	strong similarity to hexose transporter Hxt5 - Saccharomyces cerevisiae	0.65
An05g01150	hypothetical protein	0.65
An04g09490	hypothetical protein	0.65
An04g03530	strong similarity to NAD-dependent D-arabinitol dehydrogenase ard - Candida tropicalis	0.65
An04g03340	weak similarity to hypothetical syntaxin 8 related protein B13N20.240 - Neurospora crassa	0.65
An03g05120	strong similarity to sequence 247 from patent WO0100804 - Corynebacterium glutamicum	0.65
An03g01220	weak similarity to apyrase from patent GB2261878-A - Shigella flexneri	0.65
An02g13750	strong similarity to glutaminase A gtaA - Aspergillus oryzae	0.65
An02g11760	strong similarity to EST SEQ ID NO:4214 from patent WO200056762-A2 - Aspergillus niger	0.65
An02g10550	strong similarity to endo-alpha-1,5-arabinanase abnA - Aspergillus niger	0.65
An02g10480	similarity to chloride peroxidase cpo - Leptoxyphium fumago	0.65
An02g00590	strong similarity to high-affinity glucose transporter HGT1 - Kluyveromyces lactis	0.65
An02g00580	similarity to pyruvate dehydrogenase phosphatase PDPc - Bos taurus	0.65
An02g00090	strong similarity to prolidase - Aureobacterium esteraromaticum	0.65
An01g12550	strong similarity to mannosyl-oligosaccharide 1,2-alpha-mannosidase msdS - Aspergillus saitoi	0.65
An01g06780	similarity to actVA-ORF4-like protein A-ORFP from patent WO9911793-A1 - Homo sapiens	0.65
An01g05370	similarity to hypothetical protein encoded by An09g04380 - Aspergillus niger	0.65
An01g04150	strong similarity to hypothetical protein SPAC926.06c - Schizosaccharomyces pombe	0.65
An01g00510	strong similarity to cytochrome P450 cyp52A3-a - Candida maltosa	0.65
An16g07900	strong similarity to choline permease Hnm1 - Saccharomyces cerevisiae	0.65
An14g02320	weak similarity to splicing coactivator subunit SRm300 - Homo sapiens	0.65
An09g04850	strong similarity to p-sulfobenzyl alcohol dehydrogenase TsaC - Comamonas testosteroni	0.65
An04g08900	strong similarity to hypothetical protein 68B2.50 - Neurospora crassa	0.65
An18g06780	similarity to hypothetical protein B1O14.080 - Neurospora crassa	0.6
An18g05100	similarity to cytosine deaminase codA - Escherichia coli	0.6
An18g03210	strong similarity to quinone oxidoreductase P1 - Arabidopsis thaliana	0.6

An18g03040	similarity to MAP kinase phosphatase XCL100(beta) - Xenopus laevis	0.6
An18g01740	strong similarity to aliphatic nitrilase - Rhodococcus rhodochrous	0.6
An18g01180	strong similarity to penicillin V amidohydrolase PVA from patent US5516679-A - Fusarium oxysporum	0.6
An18g00960	strong similarity to choline permease Hnm1 - Saccharomyces cerevisiae	0.6
An18g00940	strong similarity to FAD dependent L-sorbose dehydrogenase SDH - Gluconobacter oxydans	0.6
An18g00170	weak similarity to kinesin - Syncephalastrum racemosum	0.6
An17g01070	strong similarity to fluconazole resistance protein FLU1 - Candida albicans [putative frameshift]	0.6
An16g09020	strong similarity to hypothetical protein SPAC922.05c - Schizosaccharomyces pombe	0.6
An16g06100	similarity to glutathione S-transferase GST1 - Ascaris suum	0.6
An16g05550	similarity to neutrophil oxidase factor - Homo sapiens	0.6
An16g03700	strong similarity to phospholipase B from patent US6146869-A - Aspergillus oryzae	0.6
An16g03690	strong similarity to iron and manganese transporter Ccc1 - Saccharomyces cerevisiae	0.6
An16g03290	similarity to regulator protein Uga3 - Saccharomyces cerevisiae	0.6
An16g02560	strong similarity to hypothetical beta-lactamase XF1621 - Xylella fastidiosa	0.6
An16g02420	weak similarity to hypothetical CG7701 - Drosophila melanogaster	0.6
An16g01880	strong similarity to lysophospholipase - Aspergillus foetidus	0.6
An16g01610	weak similarity to gamma-butyrobetaine hydroxylase BBH - Rattus norvegicus	0.6
An16g01220	similarity to hypothetical protein CAD21096.1 - Neurospora crassa	0.6
An15g07230	similarity to mitochondrial thioredoxin from patent WO9832863-A2 - Rattus sp.	0.6
An15g06580	uracil phosphoribosyltransferase furA - Aspergillus niger	0.6
An15g05960	weak similarity to hypothetical protein AAF54945.1 - Drosophila melanogaster	0.6
An15g03080	similarity to BIR repeat containing ubiquitin-conjugating enzyme BRUCE - Mus musculus	0.6
An15g02150	similarity to integral membrane protein PTH11 - Magnaporthe grisea	0.6
An15g01200	weak similarity to hypothetical protein PA3340 - Pseudomonas aeruginosa	0.6
An14g06760	similarity to hypothetical conserved protein SCIF3.07c - Streptomyces coelicolor	0.6
An14g06750	strong similarity to hypothetical oxidoreductase PA0147 - Pseudomonas aeruginosa	0.6
An14g06630	hypothetical protein	0.6
An14g05760	strong similarity to cyclohexanol dehydrogenase chnA - Acinetobacter sp.	0.6
An14g05570	similarity to EST an_3478 - Aspergillus niger	0.6
An14g03510	strong similarity to sorbitol dehydrogenase SDH - Rattus norvegicus	0.6
An14g03130	hypothetical protein	0.6
An14g03110	strong similarity to cytochrome P450 monooxygenase TRI4 - Myrothecium roridum	0.6
An13g01930	strong similarity to succinate-semialdehyde dehydrogenase SSADH - Rattus norvegicus	0.6
An13g01800	strong similarity to hypothetical protein encoded by An15g07140 - Aspergillus niger	0.6
An12g10840	hypothetical protein	0.6
An12g07690	similarity to transcription activator of lysine pathway Lys14 - Saccharomyces cerevisiae	0.6
An12g05280	weak similarity to esterase - Boophilus microplus	0.6
An11g09010	weak similarity to choline kinase R2 CK-R2 - Rattus norvegicus	0.6
An11g06450	strong similarity to hypothetical protein encoded by An02g08300 - Aspergillus niger	0.6
An11g05400	weak similarity to metacaspase casA - Aspergillus nidulans	0.6
An11g05230	similarity to hypothetical protein PA1213 - Pseudomonas aeruginosa	0.6
An11g00210	strong similarity to cyclohexanone monooxygenase chnB2 - Brevibacterium sp. [putative sequencing error]	0.6
An09g06000	weak similarity to hypothetical membrane protein YJR151c - Saccharomyces cerevisiae	0.6
An09g04880	hypothetical protein	0.6

An09g02760	hypothetical protein	0.6
An08g11680	strong similarity to 2,5-dicloro-2,5-cyclohexadiene-1,4-diol dehydrogenase linC - Pseudomonas paucimobilis	0.6
An08g09970	similarity to beta-transducin-like protein het-e - Podospora anserina	0.6
An08g09840	strong similarity to cysteine proteinase polypeptide from patent WO200268623-A2 - Aspergillus niger	0.6
An08g09420	strong similarity to protein 4MeS - Metarhizium anisopliae	0.6
An08g06430	similarity to allantoate permease Dal5 - Saccharomyces cerevisiae	0.6
An07g05830	strong similarity to formamidase fmdS - Aspergillus nidulans	0.6
An07g05490	strong similarity to stage V sporulation protein spoVK - Bacillus subtilis	0.6
An07g04040	similarity to hypothetical protein mlr7324 - Mesorhizobium loti	0.6
An07g03110	similarity to hypothetical protein CAD21104.1 - Neurospora crassa	0.6
An07g02540	similarity to carboxylic acid transport protein Jen1 - Saccharomyces cerevisiae	0.6
An07g01430	strong similarity to hypothetical protein Rv1215c - Mycobacterium tuberculosis	0.6
An07g01340	weak similarity to phosphoenolpyruvate carboxylase - Escherichia coli	0.6
An07g01200	strong similarity to enoyl reductase of the lovastatin biosynthesis lovC - Aspergillus terreus	0.6
An06g02150	similarity to hypothetical protein CAD70322.1 - Neurospora crassa	0.6
An05g02210	strong similarity to B-cell mitogen precursor PA45 - Trypanosoma cruzi	0.6
An05g02050	strong similarity to 3-oxoacyl-(acyl-carrier-protein) reductase srb - Bacillus subtilis	0.6
An05g01880	strong similarity to hypothetical chloroperoxidase cpo - Agaricus bisporus	0.6
An05g01720	similarity to hypothetical protein CAD21295.1 - Neurospora crassa	0.6
An05g00970	hypothetical protein	0.6
An05g00480	strong similarity to transcription factor involved in differentiation stuA - Aspergillus nidulans	0.6
An04g10130	3-isopropylmalate dehydrogenase leu2B - Aspergillus niger	0.6
An04g10110	similarity to hypothetical protein encoded by An11g00040 - Aspergillus niger	0.6
An04g09990	strong similarity to 2,5-dichloro-2,5-cyclohexadiene-1,4-diol dehydrogenase linC - Sphingomonas paucimobilis	0.6
An04g08890	strong similarity to aryl-alcohol oxidase precursor aao - Pleurotus eryngii	0.6
An04g08880	similarity to hypothetical protein CAD60573.1 - Podospora anserina	0.6
An04g08440	strong similarity to cytochrome P450 monooxygenase TRI11 - Fusarium sporotrichioides	0.6
An04g07460	strong similarity to ambiental pH regulatory system protein palFp - Aspergillus nidulans	0.6
An04g07430	similarity to hypothetical protein encoded by An07g09430 - Aspergillus niger	0.6
An04g07320	similarity to dark repressor of conidiation velvet veA - Aspergillus nidulans	0.6
An04g04400	similarity to tankyrase2 TANK2 from patent WO200100849-A1 - Homo sapiens	0.6
An04g03180	strong similarity to hypothetical protein EAA64005.1 - Aspergillus nidulans	0.6
An04g00900	weak similarity to beta transducin-like protein het-e1 - Podospora anserina	0.6
An03g06480	strong similarity to Steroid monooxygenase smo - Rhodococcus rhodochrous	0.6
An03g05030	hypothetical protein [truncated ORF]	0.6
An03g01530	strong similarity to xanthine dehydrogenase hxA - Aspergillus nidulans	0.6
An03g01370	strong similarity to hypothetical oxidoreductase of gene CC3685 - Caulobacter crescentus	0.6
An03g00970	strong similarity to hypothetical protein encoded by An08g12110 - Aspergillus niger	0.6
An02g13240	strong similarity to alpha-1-6-glucosidase glcA - Aspergillus parasiticus	0.6
An02g12650	hypothetical protein [truncated ORF]	0.6
An02g08470	similarity to hypothetical protein YGL079w - Saccharomyces cerevisiae	0.6
An02g08230	strong similarity to high affinity glucose transporter HGT1 - Kluyveromyces lactis	0.6
An02g07610	strong similarity to mannitol transporter mat1 - Apium graveolens	0.6
An02g05760	weak similarity to glucan 1,4-alpha-glucosidase Muc1 - Saccharomyces cerevisiae	0.6
An02g04920	similarity to hypothetical protein encoded by An09g02960 - Aspergillus niger [truncated ORF]	0.6
An02g02310	similarity to bone sialoprotein precursor IBSP - Homo sapiens	0.6
An01g12240	strong similarity to hypothetical protein CAC28784.2 - Neurospora crassa	0.6

An01g12170	strong similarity to alcohol dehydrogenase alcB - Aspergillus nidulans	0.6
An01g09020	strong similarity to hypothetical protein ymaE - Bacillus subtilis	0.6
An01g06930	strong similarity to polyketide synthase FUM5 - Gibberella moniliformis	0.6
An01g06900	weak similarity to transcription regulator of maltose utilization amyR - Aspergillus oryzae	0.6
An01g06890	similarity to peptide synthase pesA - Metarhizium anisopliae	0.6
An01g06440	similarity to hypothetical protein CAE28089.1/RPA2648 - Rhodopseudomonas palustris	0.6
An01g04140	similarity to EST an_2919 - Aspergillus niger	0.6
An01g03330	strong similarity to Pcl-like cyclin pclA - Aspergillus nidulans	0.6
An01g01950	similarity to aluminium resistance protein Alr1 - Saccharomyces cerevisiae	0.6
An01g01830	strong similarity to catalase/peroxidase cpeB - Streptomyces reticuli	0.6
An01g01720	strong similarity to bleomycin hydrolase Blh1 - Saccharomyces cerevisiae	0.6
An01g01030	weak similarity to hypothetical protein encoded by An16g05360 - Aspergillus niger	0.6
An15g04990	similarity to EST an_2920 - Aspergillus niger	0.6
An09g03470	strong similarity to hypothetical verprolin related protein encoded by B24P7.40 - Neurospora crassa	0.6
An05g02260	strong similarity to xylitol dehydrogenase xdh - Galactocandida mastotermitis	0.6
An01g05100	weak similarity to ribonucleoprotein B cp29B - Nicotiana sylvestris	0.6
An19g00290	similarity to NAD+-dependent 15-hydroxyprostaglandin dehydrogenase PDGH - Homo sapiens	0.55
An18g06570	strong similarity to hypothetical protein YGR266w - Saccharomyces cerevisiae [truncated ORF]	0.55
An18g05790	hypothetical protein	0.55
An18g05780	similarity to lanthionine synthase C-like protein 1 LANCL1 - Homo sapiens	0.55
An18g05500	strong similarity to mitochondrial ceramidase AAF86240.1 - Homo sapiens	0.55
An18g03630	similarity to hypothetical protein encoded by An07g02000 - Aspergillus niger	0.55
An18g03380	strong similarity to mitochondrial thioredoxin Trx3 - Saccharomyces cerevisiae	0.55
An18g02730	similarity to transmembrane protein PTH11 - Magnaporthe grisea	0.55
An18g02510	similarity to hypothetical protein SPAC31A2.16 - Schizosaccharomyces pombe	0.55
An18g01200	strong similarity to O-methylsterigmatocystin oxidoreductase ord1 - Aspergillus flavus	0.55
An17g02260	similarity to hnf-3/forkhead transcription factor sep1p - Schizosaccharomyces pombe [deleted ORF]	0.55
An17g00550	weak similarity to dTDP-glucose 4,6-dehydratase protein_id CAB05932.1 - Streptococcus pneumoniae	0.55
An16g09280	weak similarity to hypothetical protein AAF79893.1 - Arabidopsis thaliana	0.55
An16g09160	strong similarity to EST AN05D03 - Aspergillus niger	0.55
An16g09010	strong similarity to carboxypeptidase I protein from patent WO9814599-A1 - Aspergillus oryzae [putative frameshift]	0.55
An16g06830	hypothetical protein	0.55
An16g05640	similarity to hypothetical protein encoded by An11g07160 - Aspergillus niger	0.55
An16g04230	strong similarity to general amino acid permease Gap1 - Saccharomyces cerevisiae	0.55
An16g02820	strong similarity to fatty acid omega-hydroxylase CYP505 - Fusarium oxysporum	0.55
An16g02710	hypothetical protein	0.55
An16g02360	strong similarity to pyridoxine 4-dehydrogenase PLR - Schizosaccharomyces pombe	0.55
An16g02170	epoxide hydrolase hyl1 - Aspergillus niger	0.55
An16g01850	similarity to blastomyces yeast phase-specific protein 1 bys1 - Ajellomyces dermatitidis	0.55
An16g00920	strong similarity to peroxisomal membrane protein PMP20 - Candida boidinii	0.55
An16g00750	similarity to transcription activator Cha4 - Saccharomyces cerevisiae	0.55
An15g04830	similarity to vegetative incompatibility protein tol - Neurospora crassa	0.55
An15g04220	strong similarity to hypothetical protein EAA59582.1 - Aspergillus nidulans	0.55
An15g04150	strong similarity to oxidoreductase involved in actinorhodin production encoded by Orf11 - Streptomyces lividans	0.55
An15g02930	strong similarity to ABC transporter CDR4 - Candida albicans	0.55

An15g02470	weak similarity to hypothetical variant surface glycoprotein MVAT5 homolog - Trypanosoma brucei	0.55
An15g01970	similarity to unassigned homeobox protein Brn-3.2 - Mus sp.	0.55
An15g00120	similarity to Wilms tumor susceptibility protein WT1 - Homo sapiens	0.55
An14g06500	strong similarity to glycerone kinase isoform I dak1p - Schizosaccharomyces pombe	0.55
An14g04910	weak similarity to GABA-A receptor epsilon-like subunit epsilon - Rattus norvegicus	0.55
An14g04710	aspartic proteinase aspergillopepsin I pepA - Aspergillus niger	0.55
An14g02090	hypothetical protein	0.55
An14g00590	similarity to tartrate transport protein ttuB - Agrobacterium vitis	0.55
An14g00320	hypothetical protein	0.55
An14g00170	strong similarity to hypothetical Ca2+/H+ antiporter YNL321w - Saccharomyces cerevisiae	0.55
An14g00160	strong similarity to D-ribulokinase rbtK - Klebsiella pneumoniae	0.55
An13g03110	strong similarity to high-affinity nicotinic acid permease Tna1 - Saccharomyces cerevisiae	0.55
An13g02780	similarity to alpha-adducin alpha-ADD - Rattus norvegicus	0.55
An13g02090	strong similarity to allantoate permease Dal5 - Saccharomyces cerevisiae	0.55
An13g00980	similarity to mucin-like protein Muc1 - Saccharomyces cerevisiae	0.55
An13g00320	strong similarity to EST SEQ ID NO:4066 from patent WO200056762-A2 - Aspergillus niger	0.55
An12g09750	strong similarity to hypothetical protein CAD70289.1 - Neurospora crassa	0.55
An12g04460	similarity to EST SEQ ID NO:4349 from patent WO200056762-A2 - Aspergillus niger	0.55
An12g04090	similarity to hypothetical protein encoded by An04g09490 - Aspergillus niger	0.55
An12g02490	weak similarity to aflatoxin biosynthesis regulator aflR - Aspergillus flavus	0.55
An12g00010	questionable ORF	0.55
An11g10840	strong similarity to GTPase Ras2p - Yarrowia lipolytica	0.55
An11g10260	strong similarity to alphaN-acetylglucosamine transferase - Kluyveromyces lactis	0.55
An11g09180	similarity to mediator of mating-type-associated vegetative incompatibility tol - Neurospora crassa	0.55
An11g07830	strong similarity to hypothetical peptide transporter mtd1 - Schizophyllum commune	0.55
An11g07290	similarity to spectinomycin adenylyltransferase spc - Staphylococcus aureus	0.55
An11g06050	hypothetical protein	0.55
An11g05110	similarity to death-associated protein kinase DAPK1 - Homo sapiens	0.55
An11g03120	similarity to endo-1,4-beta-xylanase XynD - Bacillus polymyxa	0.55
An11g02570	hypothetical protein [truncated ORF]	0.55
An11g01550	strong similarity to cytochrome P450 52A4 - Candida maltosa	0.55
An11g01220	strong similarity to precursor of tannase - Aspergillus oryzae	0.55
An10g00350	similarity to GTP cyclohydrolase II ribA - Actinobacillus pleuropneumoniae	0.55
An09g06450	strong similarity to hypothetical protein CAD71235.1 - Neurospora crassa [truncated ORF]	0.55
An09g06200	strong similarity to transmembrane protein PTH11 - Magnaporthe grisea	0.55
An09g04540	strong similarity to hypothetical protein EAA63983.1 - Aspergillus nidulans [truncated ORF]	0.55
An09g03380	similarity to hypothetical protein B24P7.110 - Neurospora crassa	0.55
An09g03330	similarity to hypothetical protein encoded by SCE66.10c - Streptomyces coelicolor	0.55
An09g02830	strong similarity to acylaminoacyl-peptidase DPP V - Aspergillus fumigatus	0.55
An09g02560	similarity to polyamine oxidase PAO - Zea mays	0.55
An09g01190	endo 1,5-alpha-arabinanase abnA - Aspergillus niger	0.55
An09g00710	similarity to haloacetate dehalogenase H-1 - Moraxella sp.	0.55
An09g00680	strong similarity to hypothetical protein SC9B2.03 - Streptomyces coelicolor	0.55
An08g09940	strong similarity to cytochrome P450 monooxygenase TRI11 - Fusarium sporotrichioides	0.55
An08g09780	weak similarity to hypothetical protein SC8F4.26 - Streptomyces coelicolor	0.55
An08g08930	similarity to hypothetical protein CAE41807.1 - Bordetella pertussis	0.55

An08g08440	similarity to RNA-directed RNA polymerase RdRP - Lycopersicon esculentum	0.55
An08g08370	similarity to alpha-1,2-mannosidase aman2 - Bacillus sp.	0.55
An08g08250	strong similarity to aryl-alcohol oxidase precursor aao - Pleurotus pulmonarius	0.55
An08g05790	strong similarity to glycogen phosphorylase Gph1 - Saccharomyces cerevisiae	0.55
An08g05350	strong similarity to hypothetical protein encoded by An18g00300 - Aspergillus niger	0.55
An08g05070	similarity to hypothetical protein SCIF3.09c - Streptomyces coelicolor	0.55
An08g04490	endoprotease Endo-Pro - Aspergillus niger	0.55
An08g04350	similarity to chitin synthase III like Chs7 - Saccharomyces cerevisiae	0.55
An08g03060	strong similarity to hypothetical protein CC0533 - Caulobacter crescentus	0.55
An08g02670	strong similarity to EST an_3167 - Aspergillus niger	0.55
An08g01950	strong similarity to hypothetical 30.2 KD protein Y4OV - Rhizobium sp.	0.55
An07g09090	weak similarity to hypothetical protein encoded by An18g02770 - Aspergillus niger	0.55
An07g08980	strong similarity to phosphatidylinositol 3-phosphate 5-kinase Fab1 - Saccharomyces cerevisiae	0.55
An07g08650	hypothetical protein	0.55
An07g06480	similarity to cytochrome 4F8 cyp4F8 - Homo sapiens	0.55
An07g06310	strong similarity to FAD dependent L-sorbose dehydrogenase SDH - Gluconobacter oxydans	0.55
An07g06230	hypothetical protein	0.55
An07g05970	strong similarity to ankyrin Ank2 - Homo sapiens	0.55
An07g04490	weak similarity to trans-Golgi p230 - Homo sapiens	0.55
An07g04480	strong similarity to hypothetical protein EAA64298.1 - Aspergillus nidulans	0.55
An07g04120	hypothetical protein	0.55
An07g04060	strong similarity to EST SEQ ID NO:4312 from patent WO200056762-A2 - Aspergillus niger	0.55
An07g03530	hypothetical protein	0.55
An07g02060	similarity to myo-inositol transporter 2 - Schizosaccharomyces pombe	0.55
An07g02050	2,3-dihydroxybenzoic acid decarboxylase dhbD - Aspergillus niger	0.55
An06g01050	strong similarity to hypothetical protein SPAC17G6.02c - Schizosaccharomyces pombe	0.55
An05g02410	strong similarity to beta-glucuronidase GUSB - Canis lupus	0.55
An05g01810	strong similarity to 2-nitropropane dioxygenase precursor ncd-2 - Neurospora crassa	0.55
An05g01490	strong similarity to hypothetical protein PA3762 - Pseudomonas aeruginosa	0.55
An04g05880	strong similarity to linoleate diol synthase - Gaeumannomyces graminis	0.55
An04g05780	questionable ORF	0.55
An04g02700	similarity to wheat raffinose synthase from patent WO200024915-A2 - Triticum aestivum	0.55
An04g02670	strong similarity to NADPH-dependent aldehyde reductase - Sporobolomyces salmonicolor	0.55
An04g01430	weak similarity to hypothetical protein encoded by B11A5.120 - Neurospora crassa	0.55
An04g00340	strong similarity to myo-inositol transport protein Itr2 - Saccharomyces cerevisiae	0.55
An03g06670	weak similarity to myosin-like protein Mlp1 - Saccharomyces cerevisiae	0.55
An03g06660	strong similarity to peptide transporter PTR2 - Arabidopsis thaliana	0.55
An03g06330	strong similarity to 2,3-dihydroxybiphenyl-1,2-dioxygenase bphC - Pseudomonas stutzeri	0.55
An03g06140	strong similarity to hypothetical protein encoded by 123A4.300 - Neurospora crassa	0.55
An03g06090	strong similarity to molasses resistency protein Rtm1 - Saccharomyces cerevisiae	0.55
An03g05040	similarity to EST an_2087 - Aspergillus niger	0.55
An03g04480	hypothetical protein	0.55
An03g02800	strong similarity to EST an_2890 - Aspergillus niger	0.55
An03g02300	strong similarity to metal-activated pyridoxal enzyme D-threonine aldolase - Arthrobacter sp.	0.55
An03g01460	strong similarity to gibberellin 7-oxidase - Cucurbita maxima	0.55

An03g00330	hypothetical protein	0.55
An02g13980	strong similarity to trichodiene oxygenase cytochrome P450 CYP58 - Fusarium sporotrichioides	0.55
An02g13800	strong similarity to hypothetical membrane protein YPR157w - Saccharomyces cerevisiae	0.55
An02g12600	similarity to hypothetical hydrolase MJ0301 - Methanococcus jannaschii	0.55
An02g11150	alpha-galactosidase aglB - Aspergillus niger	0.55
An02g08570	strong similarity to isopenicillin N acyltransferase aAT - Aspergillus nidulans	0.55
An02g07940	strong similarity to hypothetical protein YEL023c - Saccharomyces cerevisiae	0.55
An02g07410	hypothetical protein	0.55
An02g05240	strong similarity to histone 4 from patent WO9919502-A1 - Homo sapiens	0.55
An02g05120	hypothetical protein	0.55
An02g04850	similarity to protein SEQ ID NO:7804 from patent WO200253728-A2 - Candida albicans	0.55
An02g03740	weak similarity to protein-tyrosine kinase erbB2 - Homo sapiens	0.55
An02g03380	strong similarity to hypothetical protein EAA61757.1 - Aspergillus nidulans	0.55
An02g01550	strong similarity to secreted serine protease 19 kDa CS antigen CS-Ag - Coccidioides immitis	0.55
An02g01260	weak similarity to beta transducin-like protein het-e1 - Podospora anserina	0.55
An02g00080	strong similarity to NAD-GSH-dependent formaldehyde dehydrogenase flhA - Paracoccus denitrificans	0.55
An01g14940	similarity to nonhemolytic phospholipase C PC-PLC - Burkholderia pseudomallei	0.55
An01g14520	strong similarity to carbohydrate oxidase CHO from patent WO9931990-A1 - Microdochium nivale	0.55
An01g13700	similarity to calcineurin responsive zinc-finger transcription factor Crz1 - Saccharomyces cerevisiae	0.55
An01g13000	strong similarity to glu/asp-tRNA amidotransferase subunit A gatA - Thermus thermophilus	0.55
An01g12050	similarity to 15-decalonectrin 15-O-acetyltransferase TRI3 - Fusarium sporotrichioides	0.55
An01g11580	similarity to hypothetical protein encoded by An01g11570 - Aspergillus niger	0.55
An01g10930	strong similarity to enzyme with sugar transferase activity from patent JP11009276-A - Acremonium sp.	0.55
An01g10920	strong similarity to sorbitol dehydrogenase SDH - Rattus norvegicus	0.55
An01g07660	similarity to hypothetical protein B7F18.80 - Neurospora crassa	0.55
An01g06880	similarity to dihydroflavonol 4-reductase BAA12723.1 - Rosa hybrid cultivar	0.55
An01g06710	similarity to hypothetical phosphorylase AAM06921.1 - Methanosarcina acetivorans	0.55
An01g05600	hypothetical protein	0.55
An01g05360	strong similarity to 42 kDa endochitinase Tham-ch - Trichoderma hamatum	0.55
An01g04530	similarity to hypothetical MAP3K protein kinase-like protein MLD14.3 - Arabidopsis thaliana	0.55
An01g01000	similarity to hypothetical protein BAC11036.1 - Homo sapiens	0.55
An18g01700	strong similarity to quinate transport protein qutD - Aspergillus nidulans	0.55
An16g01930	hypothetical protein	0.55
An16g01690	similarity to X-linked PEST-containing monocarboxylate transporter XPCT - Homo sapiens	0.55
An15g06780	similarity to vesicular acetylcholine transporter VAChT - Homo sapiens	0.55
An15g02280	similarity to WW domain-containing oxidoreductase WOX1 - Mus musculus	0.55
An11g06440	strong similarity to enoyl reductase of the lovastatin biosynthesis lovC - Aspergillus terreus	0.55
An04g00040	strong similarity to S-malonyltransferase fabD - Bacillus subtilis	0.55
An02g11720	strong similarity to alpha-mannosidase msd2 - Aspergillus nidulans	0.55
An02g02080	hypothetical protein	0.55
An02g01330	similarity to hypothetical nadh-dependent flavin oxidoreductase SPBC23G7.10c - Schizosaccharomyces pombe	0.55
An01g11780	weak similarity to dATP pyrophosphohydrolase NMB0642 - Neisseria meningitidis	0.55
An19g00300	strong similarity to kinesin light chain KLC - Plectonema boryanum	0.5

An19g00210	similarity to hemolysin ASP-HS - Aspergillus fumigatus	0.5
An18g06310	strong similarity to L-fucose permease fucP - Escherichia coli	0.5
An18g05210	similarity to multifunctional beta-oxidation protein - Neurospora crassa	0.5
An18g03170	strong similarity to cyclin-dependent protein kinase Pho85 - Saccharomyces cerevisiae	0.5
An18g02770	weak similarity to hypothetical protein encoded by An07g09090 - Aspergillus niger	0.5
An18g02600	hypothetical protein	0.5
An18g02150	similarity to hypothetical protein CAF05965.1 - Neurospora crassa	0.5
An18g01010	strong similarity to fluconazole resistance protein FLU1 - Candida albicans	0.5
An18g01000	strong similarity to EST an_2192 - Aspergillus niger	0.5
An18g00340	hypothetical protein	0.5
An17g02370	strong similarity to uracil phosphoribosyltransferase Fur1 - Saccharomyces cerevisiae [putative frameshift]	0.5
An17g00620	hypothetical protein	0.5
An17g00180	hypothetical protein	0.5
An16g07920	strong similarity to hypothetical protein EAA64623.1 - Aspergillus nidulans	0.5
An16g07710	similarity to cocaine esterase cocE - Rhodococcus sp.	0.5
An16g07500	strong similarity to acetamidase amdS - Aspergillus nidulans	0.5
An16g07150	strong similarity to soluble cytoplasmic fumarate reductase YEL047c - Saccharomyces cerevisiae	0.5
An16g06740	strong similarity to cadmium resistance protein Ycf1 - Saccharomyces cerevisiae [truncated ORF]	0.5
An16g05340	similarity to trans-2-enoyl-ACP reductase II fabK - Streptococcus pneumoniae	0.5
An16g04500	strong similarity to meiosis control kinase ran1p - Schizosaccharomyces pombe	0.5
An16g04220	similarity to 2-haloacid halidohydrolase IVa - Pseudomonas cepacia	0.5
An16g04060	similarity to hypothetical protein BAB55393.1 - Homo sapiens	0.5
An16g04040	weak similarity to membrane protein yeeO - Escherichia coli	0.5
An16g03270	hypothetical protein	0.5
An16g03040	strong similarity to hypothetical AMMECR1 - Homo sapiens	0.5
An16g02760	strong similarity to hypothetical protein BH0842 - Bacillus halodurans	0.5
An16g01680	strong similarity to gluthathione S-transferase III GST3 - Zea mays	0.5
An16g01630	strong similarity to enoyl reductase of the lovastatin biosynthesis lovC - Aspergillus terreus	0.5
An16g01490	similarity to ubiquitin carboxyl-terminal hydrolase uch2p - Schizosaccharomyces pombe	0.5
An16g01020	similarity to hypothetical transcription regulator SPBC530.05 - Schizosaccharomyces pombe	0.5
An16g00830	similarity to hypothetical protein encoded by An14g02830 - Aspergillus niger	0.5
An16g00810	strong similarity to oligopeptide transporter Opt1 - Saccharomyces cerevisiae	0.5
An15g07200	similarity to hypothetical transmembrane protein - Erysiphe pisi	0.5
An15g07090	strong similarity to hypothetical protein encoded by An12g10350 - Aspergillus niger	0.5
An15g06560	weak similarity to hypothetical proline-rich protein T20D16.24 - Arabidopsis thaliana	0.5
An15g02590	strong similarity to dimethylglycine dehydrogenase precursor DMGDH - Homo sapiens	0.5
An15g01580	weak similarity to cell envelope protein 05ae20220orf32 from patent WO9640893-A1 - Helicobacter pylori	0.5
An15g01220	hypothetical protein	0.5
An15g00280	strong similarity to hypothetical protein jhp0295 - Helicobacter pylori	0.5
An14g07190	similarity to hypothetical protein CAD21260.1 - Neurospora crassa	0.5
An14g05840	strong similarity to O-methyltransferase omtB - Aspergillus flavus	0.5
An14g05480	hypothetical protein	0.5
An14g05410	strong similarity to hypothetical protein CAD21189.1 - Neurospora crassa	0.5
An14g04550	hypothetical protein	0.5
An14g04240	strong similarity to hypothetical conserved protein CC0533 - Caulobacter crescentus	0.5

An14g04190	strong similarity to 1,4-alpha-glucan branching enzyme Glc3 - Saccharomyces cerevisiae	0.5
An14g03090	strong similarity to glucose/galactose transporter gluP - Brucella abortus	0.5
An14g02470	strong similarity to protein PRO304 from patent WO200104311-A1 - Homo sapiens	0.5
An14g02140	weak similarity to Ca-dependent protein kinase CDPK1 - Marchantia polymorpha	0.5
An14g02080	strong similarity to prolidase - Aureobacterium esteraromaticum	0.5
An14g01790	hypothetical protein	0.5
An14g01680	similarity to monocarboxylate transporter MCT3 - Rattus norvegicus	0.5
An14g01660	strong similarity to cyclohexanone monooxygenase chnB2 - Brevibacterium sp.	0.5
An14g01520	similarity to hypothetical protein encoded by An12g09920 - Aspergillus niger	0.5
An14g00640	strong similarity to hypothetical protein EAA61679.1 - Aspergillus nidulans	0.5
An13g03600	strong similarity to phosphatidylcholine-hydrolyzing phospholipase C PLCN - Burkholderia pseudomallei	0.5
An13g02410	weak similarity to hypothetical cation transporter DRA0361 - Deinococcus radiodurans	0.5
An13g02080	strong similarity to ATP-hydrolyzing 5-oxoprolinase - Rattus norvegicus	0.5
An13g00950	strong similarity to alcohol dehydrogenase alcB - Aspergillus nidulans	0.5
An13g00130	hypothetical protein	0.5
An12g10630	similarity to acid phosphatase aphA - Aspergillus ficuum	0.5
An12g10220	similarity to leucoanthocyanidin dioxygenase LDOX - Vitis vinifera	0.5
An12g10030	weak similarity to membrane protein YIL140w Rev7 - Saccharomyces cerevisiae	0.5
An12g09190	similarity to nucleic acid-binding protein NuABP-10 from patent WO200044900-A2 - Homo sapiens	0.5
An12g09180	hypothetical protein	0.5
An12g08620	strong similarity to MFS drug transporter Bcmfs1 - Botryotinia fuckeliana	0.5
An12g08270	strong similarity to L-lactate 2-monooxygenase LA2M - Mycobacterium smegmati	0.5
An12g07860	strong similarity to esterase P1-8LC from patent WO9730160-A1 - Sulfolobus solfataricus	0.5
An12g05360	strong similarity to cholesterol 24-hydroxylase - Mus musculus	0.5
An12g05220	similarity to transcription factor nft1p - Schizosaccharomyces pombe	0.5
An12g04990	strong similarity to glucitol 6-phosphate dehydrogenase gutD - Clostridium beijerinckii	0.5
An12g03760	similarity to hypothetical protein encoded by An12g03780 - Aspergillus niger	0.5
An12g03500	weak similarity to hypothetical protein CAD71089.1 - Neurospora crassa	0.5
An12g03460	strong similarity to proline permease prnB - Aspergillus nidulans	0.5
An12g03280	hypothetical protein	0.5
An12g02180	similarity to hypothetical protein encoded by An12g09970 - Aspergillus niger	0.5
An12g01950	strong similarity to hypothetical protein CAD60583.1 - Podospora anserina	0.5
An12g01870	similarity to positive regulator of the lactose-galactose regulon LAC9 - Kluyveromyces lactis	0.5
An12g01850	strong similarity to glycosidase from patent WO9824799-A1 - Thermotoga maritima	0.5
An12g01540	similarity to hypothetical protein dJ1042K10.5 - Homo sapiens	0.5
An12g00850	similarity to beta transducin-like protein het-e1 - Podospora anserina	0.5
An12g00600	similarity to monodehydroascorbate reductase MDA - Cucumis sativus	0.5
An11g11320	strong similarity to protein involved in autophagy Aut2 - Saccharomyces cerevisiae	0.5
An11g11220	hypothetical protein	0.5
An11g11130	strong similarity to 26S proteasome-associated ubiquitin carboxyl-terminal hydrolase uch2p - Schizosaccharomyces pombe	0.5
An11g10780	similarity to hypothetical conserved protein B8L3.060 - Neurospora crassa [truncated ORF]	0.5
An11g10160	strong similarity to hypothetical oxetanocin A resistance protein oxrB - Bacillus megaterium	0.5
An11g09240	similarity to Gpi-anchored aspartic protease Yps1 - Saccharomyces cerevisiae	0.5
An11g07940	hypothetical protein	0.5
An11g07720	hypothetical protein	0.5

An11g07060	weak similarity to actinomycin synthase III acmC - Streptomyces chrysomallus	0.5
An11g07030	similarity to plasma membrane protein Pth11 - Magnaporthe grisea	0.5
An11g03780	strong similarity to hypothetical transcription regulator SPBC530.05 - Schizosaccharomyces pombe	0.5
An11g03640	strong similarity to transmemrane oligopeptide transporter OPT1 - Candida albicans	0.5
An11g03450	strong similarity to hypothetical protein dag11 - Agaricus bisporus	0.5
An11g03240	strong similarity to NAD-dependent D-arabinitol dehydrogenase ard - Candida tropicalis	0.5
An11g03100	strong similarity to cyclin-dependent protein kinase phoa - Aspergillus nidulans	0.5
An11g02700	similarity to transcriptional activator Haa1 - Saccharomyces cerevisiae	0.5
An11g02540	strong similarity to mitochondrial 2-oxoglutarate/malate translocator clone OMT103 - Panicum miliaceum	0.5
An11g01970	similarity to pyroglutamyl-peptidase I PGPI - Pyrococcus furiosus	0.5
An11g01720	strong similarity to high-affinity nicotinic acid permease Tna1 - Saccharomyces cerevisiae	0.5
An11g01480	weak similarity to hypothetical RNA polymerase - Pelargonium leaf curl virus	0.5
An11g01250	weak similarity to cyclin-dependent kinase Cdk8 - Drosophila melanogaster	0.5
An11g01200	similarity to hypothetical protein CAD21260.1 - Neurospora crassa	0.5
An09g04360	weak similarity to hypothetical protein SPy1903 - Streptococcus pyogenes	0.5
An09g04010	strong similarity to chitin synthase C chsC - Aspergillus fumigatus	0.5
An09g03730	strong similarity to acyl CoA dehydrogenase aidB - Escherichia coli	0.5
An09g02090	weak similarity to hypothetical protein encoded by An12g05320 - Aspergillus niger	0.5
An09g01820	strong similarity to 4-coumarate-CoA ligase 4CL - Arabidopsis thaliana	0.5
An09g01050	similarity to platelet-activating factor-acetylhydrolase - Cavia porcellus	0.5
An08g10210	weak similarity to suppressor protein SEF1 - Kluyveromyces lactis	0.5
An08g10190	weak similarity to hypothetical protein VeA - Neurospora crassa	0.5
An08g09850	strong similarity to phosphate-repressible acid phosphatase precursor phoA - Penicillium chrysogenum	0.5
An08g08910	strong similarity to mitochondrial sulfite oxidase SUOX - Homo sapiens	0.5
An08g08400	strong similarity to toluenesulfonate zinc-independent alcohol dehydrogenase TsaC - Comamonas testosteroni	0.5
An08g07320	strong similarity to hypothetical conserved protein SCD10.11 - Streptomyces coelicolor	0.5
An08g07280	strong similarity to methicillin resistance gene HmrA - Staphylococcus aureus	0.5
An08g06730	weak similarity to hypothetical protein CAD29600.1 - Aspergillus fumigatus	0.5
An08g06250	strong similarity to benzoate 4-monooxygenase cytochrome P450 53 bphA - Aspergillus niger	0.5
An08g05230	strong similarity to hypothetical endoglucanase IV - Trichoderma reesei	0.5
An08g04040	strong similarity to quinate transport protein qutD - Aspergillus nidulans	0.5
An08g03360	strong similarity to protein kinase kin1p - Schizosaccharomyces pombe	0.5
An08g02330	strong similarity to multidrug resistance protein MLP-2 - Rattus norvegicus	0.5
An08g02090	hypothetical protein	0.5
An08g01810	similarity to S-layer protein - Clostridium thermocellum [truncated ORF]	0.5
An08g01710	strong similarity to alpha-L-arabinofuranosidase abfA - Bacillus stearothermophilus	0.5
An08g01500	weak similarity to hypothetical protein T4D2.50 - Arabidopsis thaliana	0.5
An08g00540	strong similarity to EST SEQ ID NO:4140 from patent WO200056762-A2 - Aspergillus niger	0.5
An07g10020	strong similarity to microtubule-associated protein Aut7 - Saccharomyces cerevisiae	0.5
An07g09590	strong similarity to glutathione S-transferase GST from patent US5962229-A - Zea mays	0.5
An07g09060	strong similarity to hypothetical protein CAE76244.1 - Neurospora crassa	0.5
An07g08630	strong similarity to hypothetical protein At2g25280 - Arabidopsis thaliana	0.5
An07g08200	weak similarity to secreted form of protein F - Human respiratory syncytial virus	0.5
An07g07700	similarity to hypothetical glycosyl hydrolase BAC68337.1 - Streptomyces avermitilis	0.5

An07g06780	strong similarity to hypothetical phosphomannomutase homolog - Schizosaccharomyces pombe	0.5
An07g06400	strong similarity to copper amine oxidase AO-I - Aspergillus niger	0.5
An07g06150	strong similarity to lanosterol synthase erg7p - Schizosaccharomyces pombe	0.5
An07g05840	strong similarity to multidrug resistance protein Hol1 - Saccharomyces cerevisiae	0.5
An07g05210	strong similarity to long-chain-fatty-acid acyl-CoA ligase fenL - Bacillus subtilis	0.5
An07g05020	weak similarity to regulator protein Uga3 - Saccharomyces cerevisiae	0.5
An07g04650	similarity to glucan 1,3-beta-glucosidase Bgl2 - Saccharomyces cerevisiae	0.5
An07g04360	hypothetical protein	0.5
An07g04320	strong similarity to NAD-dependent D-arabinitol dehydrogenase ard - Candida tropicalis	0.5
An07g04160	strong similarity to FLO11 gene expression regulator An34 from patent WO200257456-A2 - Unclassified organism	0.5
An07g02950	hypothetical protein	0.5
An07g02880	strong similarity to hypothetical protein EAA57627.1 - Aspergillus nidulans	0.5
An07g01690	weak similarity to hypothetical protein CAC28841.2 - Neurospora crassa	0.5
An07g01410	hypothetical protein	0.5
An07g00150	strong similarity to multidrug transporter bmr3 - Bacillus subtilis	0.5
An06g01890	similarity to histidine triad protein Hnt1 - Saccharomyces cerevisiae	0.5
An06g01440	similarity to heterogeneous nuclear ribonucleoprotein (hnRNP) Tom34 - Saccharomyces cerevisiae	0.5
An06g00930	weak similarity to hypothetical protein CAD21295.1 - Neurospora crassa	0.5
An06g00500	hypothetical protein	0.5
An06g00200	hypothetical protein	0.5
An05g02280	similarity to esterase from patent WO9802556-A2 - Alcaligenes sp.	0.5
An05g00390	strong similarity to acetyl coenzyme A synthase - Tetrahymena pyriformis	0.5
An05g00300	similarity to microsomal cytochrome b5 cytb5 - Musca domestica	0.5
An05g00190	similarity to cyclin like protein Pcl7 - Saccharomyces cerevisiae	0.5
An05g00020	weak similarity to transcription factor Ume6 - Saccharomyces cerevisiae	0.5
An04g09900	strong similarity to hypothetical protein encoded by An07g06270 - Aspergillus niger	0.5
An04g08730	strong similarity to hypothetical conserved protein SPAC12B10.16c - Schizosaccharomyces pombe	0.5
An04g08620	similarity to heme activator protein Hap1 - Saccharomyces cerevisiae	0.5
An04g06000	similarity to transcription activator Put3 - Saccharomyces cerevisiae	0.5
An04g05280	similarity to mucin-like protein Muc1 - Saccharomyces cerevisiae	0.5
An04g05070	hypothetical protein	0.5
An04g05060	similarity to protein XFIN - Xenopus laevis	0.5
An04g04340	strong similarity to nonaketide synthase lovB - Aspergillus terreus	0.5
An04g03440	similarity to novel polypeptide sequence SEQ ID NO:1786 from patent WO2003029271-A2 - Homo sapiens	0.5
An04g03290	strong similarity to long-chain acyl-CoA dehydrogenase - Rattus norvegicus	0.5
An04g02840	strong similarity to flavocytochrome b2 L-lactate dehydrogenase CYB2 - Pichia anomala [putative frameshift]	0.5
An04g02300	similarity to trans-2-enoyl-ACP reductase II fabK - Streptococcus pneumoniae	0.5
An04g00550	strong similarity to EST an_1834 - Aspergillus niger	0.5
An03g06980	similarity to hypothetical protein B24M22.160 - Neurospora crassa [truncated ORF]	0.5
An03g06460	strong similarity to hypothetical sterigmatocystin biosynthesis p450 monooxygenase stcB - Aspergillus nidulans	0.5
An03g06390	strong similarity to 3-(3-hydroxyphenyl)propionate hydroxylase MhpA - Comamonas testosteroni	0.5
An03g05110	similarity to hypothetical protein BAB11562.1 - Arabidopsis thaliana	0.5
An03g04840	hypothetical protein	0.5
An03g04770	similarity to hypothetical protein CAD11383.1 - Neurospora crassa	0.5
An03g04690	strong similarity to hypothetical osmosensor Sho1p - Candida utilis	0.5
An03g03300	similarity to almond N-glycosidase from patent EP676473-A2 - Prunus dulcis	0.5
An03g03010	similarity to hypothetical serine/threonine protein phosphatase topA - Fervidobacterium islandicum	0.5

An03g02690	strong similarity to hypothetical protein SCF91.02c - Streptomyces coelicolor	0.5
An03g01550	similarity to gluconate:NADP 5-oxidoreductase GNO - Gluconobacter oxydans [truncated ORF]	0.5
An03g01130	strong similarity to hypothetical protein encoded by An07g06330 - Aspergillus niger	0.5
An03g00690	hypothetical protein	0.5
An03g00640	similarity to neutral amino acid permease mtr - Neurospora crassa [truncated ORF]	0.5
An02g14910	similarity to human peptidase HPEP-14 from patent WO200042201-A2 - Homo sapiens [truncated orf]	0.5
An02g13300	similarity to hypothetical protein ID880 - Bradyrhizobium japonicum	0.5
An02g13180	strong similarity to beta-1,3-exoglucanase lam1.3 - Trichoderma atroviride	0.5
An02g12930	similarity to tetracycline efflux pump tcrC - Streptomyces aureofaciens	0.5
An02g11650	hypothetical protein	0.5
An02g09810	strong similarity to mitochondrial nicotinamide nucleotide transhydrogenase NNTM - Bos taurus	0.5
An02g09090	strong similarity to mutarotase enzyme from patent JP07039380-A - Sus scrofa	0.5
An02g08810	hypothetical protein	0.5
An02g07930	strong similarity to precursor of linoleate diol synthase - Gaeumannomyces graminis	0.5
An02g07340	similarity to hypothetical protein CAE76294.1 - Neurospora crassa	0.5
An02g07290	weak similarity to hypothetical protein encoded by An11g05410 - Aspergillus niger	0.5
An02g05920	strong similarity to GABA permease Uga4 - Saccharomyces cerevisiae	0.5
An02g05330	hypothetical protein	0.5
An02g04970	hypothetical protein	0.5
An02g04910	strong similarity to multidrug resistance ABC transporter bfr1p - Schizosaccharomyces pombe	0.5
An02g04110	similarity to phenylacetaldehyde dehydrogenase padA - Escherichia coli	0.5
An02g03700	weak similarity to nicotinic acetylcholine receptor alpha-4 chain ACh4 - Rattus norvegicus	0.5
An02g03450	weak similarity to hypothetical protein AAA72367.1 - Homo sapiens	0.5
An02g02920	strong similarity to triosephosphate isomerase tpiA - Bacillus stearothermophilus	0.5
An02g02870	strong similarity to alcohol dehydrogenase adhIII - Aspergillus nidulans	0.5
An02g01410	hypothetical protein	0.5
An01g14020	similarity to sterigmatocystin synthesis transcription regulator aflR - Aspergillus nidulans	0.5
An01g13500	hypothetical protein	0.5
An01g13480	strong similarity to protein SEQ ID NO:7805 from patent WO200253728-A2 - Candida albicans	0.5
An01g10620	hypothetical protein	0.5
An01g09980	strong similarity to hemolysin Asp-HS - Aspergillus fumigatus	0.5
An01g09460	similarity to glucoamylase III Wsc2 - Saccharomyces cerevisiae [putative frameshift]	0.5
An01g08600	similarity to hypothetical protein At2g26280 - Arabidopsis thaliana	0.5
An01g06920	strong similarity to multidrug resistance protein ABCC2 - Homo sapiens	0.5
An01g06830	similarity to 3-ketosphinganine reductase Tsc10 - Saccharomyces cerevisiae	0.5
An01g06120	strong similarity to 4-alpha-glucanotransferase / amylo-1,6-glucosidase Gdb1 - Saccharomyces cerevisiae	0.5
An01g05960	similarity to cyanovirin-N CV-N - Nostoc ellipsosporum	0.5
An01g05920	strong similarity to hypothetical methyltransferase AAO34671.1 - Gibberella zeae	0.5
An01g04970	strong similarity to lysosomal cystine transporter cystinosin CTNS - Homo sapiens	0.5
An01g04560	strong similarity to mixed-linked glucanase precursor MLG1 - Cochliobolus carbonum	0.5
An01g03290	weak similarity to hypothetical lipoprotein nlpD - Synechocystis sp. [truncated ORF]	0.5
An01g02240	similarity to 1-aminocyclopropane-1-carboxylic acid oxidase - Cucumis sativus	0.5
An01g02110	similarity to hypothetical protein CAD11409.1 - Neurospora crassa	0.5

An01g01990	hypothetical protein	0.5
An01g00680	strong similarity to cytochrome P450 monooxygenase avnA - Aspergillus parasiticus	0.5
An01g00520	hypothetical protein	0.5
An16g04510	strong similarity to EST an_0311 - Aspergillus niger	0.5
An16g02910	strong similarity to hypothetical protein CC0533 - Caulobacter crescentus	0.5
An16g01710	strong similarity to L-iditol 2-dehydrogenase (sorbitol dehydrogenase) SORD - Homo sapiens	0.5
An15g00290	strong similarity to acetamidase amdS - Aspergillus nidulans	0.5
An12g05550	hypothetical protein	0.5
An11g03380	similarity to monoglyceride lipase mgll - Mus musculus	0.5
An08g10110	strong similarity to lipid transfer protein POX18 - Candida tropicalis	0.5
An08g09430	similarity to notchless Nle - Drosophila melanogaster	0.5
An07g10320	strong similarity to hypothetical conserved protein B13I18.30 - Neurospora crassa	0.5
An04g04170	strong similarity to NAD-dependent D-arabinitol dehydrogenase ard - Candida tropicalis	0.5
An04g01600	strong similarity to hypothetical protein B1D1.80 - Neurospora crassa	0.5
An03g02500	similarity to transmembrane protein from patent WO9927105-A2 - Chlamydia pneumoniae	0.5
An02g14850	similarity to barley containing SNP protein fragment SEQ ID NO:660 from patent WO2003057877-A1 - Hordeum vulgare	0.5
An02g02340	strong similarity to chitin synthase with a myosin motor-like domain csmA - Aspergillus nidulans	0.5
An01g06500	strong similarity to filamentous growth protein Dfg5 - Saccharomyces cerevisiae	0.5
An01g01660	weak similarity to alkyl salicylate esterase salE - Acinetobacter sp.	0.5
