# Supplementary material for: A Transcriptome Meta-Analysis Proposes Novel Biological Roles for the Antifungal Protein AnAFP in Aspergillus niger
Source: PLoS One. 2016 Nov 11;11(11):e0165755. doi: 10.1371/journal.pone.0165755 (PMC5106034; doi:10.1371/journal.pone.0165755)
Supplement: S3 Table — Pairwise correlation coefficients (Spearman coefficient) for the expression profiles of all ~ 14.000 A. niger genes under the 155 different cultivations present in our in-house transcriptomic database were calculated using Bioconductor [52]. Pairs with a Spearman score ≥ 0.5 were considered to be significantly co-expressed. (PDF) [file pone.0165755.s008.pdf]

S3 Table

Gene ID	Annotation	Spearman coefficient
An08g00760	similarity to RNA helicase Prp22 - Saccharomyces cerevisiae	-0.75
An16g02510	strong similarity to alcohol dehydrogenase C ADHC - Mycobacterium smegmatis	-0.65
An15g01650	similarity to tetracycline resistance protein TetH - Pasteurella multocida	-0.65
An12g08570	similarity to type 2 peroxiredoxin PrxII - Brassica napus	-0.65
An09g05010	strong similarity to choline permease Hnm1 - Saccharomyces cerevisiae	-0.65
An09g03110	strong similarity to alpha-amylase precursor amy - Bacillus amyloliquefaciens	-0.65
An08g06370	strong similarity to GTP-cyclohydrolase II RIB1 - Pichia guilliermondii	-0.65
An04g04970	strong similarity to Yta11 - Saccharomyces cerevisiae	-0.65
An02g01730	strong similarity to mitochondrial dicarboxylate transport protein Dtp - Saccharomyces cerevisiae	-0.65
An01g11930	strong similarity to histidinol-phosphate transaminase his3p - Schizosaccharomyces pombe	-0.65
An18g06760	strong similarity to NAD(+)-isocitrate dehydrogenase subunit I idh1 - Ajellomyces capsulatus	-0.6
An18g05880	similarity to nucleolar protein Nulp1 - Mus musculus	-0.6
An16g03130	strong similarity to mitochondrial asparagine--tRNA ligase YCR024c - Saccharomyces cerevisiae	-0.6
An15g07500	strong similarity to ribokinase rbsK - Escherichia coli	-0.6
An15g02360	strong similarity to acetylornithine aminotransferase arg8 - Kluyveromyces lactis	-0.6
An15g02320	strong similarity to chaperone protein Atp11 - Saccharomyces cerevisiae	-0.6
An14g04090	strong similarity to hypothetical protein she9 - Candida albicans	-0.6
An13g01080	strong similarity to ATP phosphoribosyltransferase his1p - Schizosaccharomyces pombe [putative sequencing error]	-0.6
An12g04930	strong similarity to hypothetical mitochondrial carrier protein SPBP23A10.06 - Schizosaccharomyces pombe	-0.6
An12g03940	strong similarity to hypothetical pyridoxine synthesis protein PDX2 - Cercospora nicotianae	-0.6
An11g02760	strong similarity to protein involved in vesicle transport in the secretory pathway YAL048c - Saccharomyces cerevisiae	-0.6
An11g02650	strong similarity to hypothetical gtpase activating protein SPAC824.09c - Schizosaccharomyces pombe	-0.6
An09g05860	strong similarity to methylenetetrahydrofolate reductase (NADPH) Met12 - Saccharomyces cerevisiae	-0.6
An09g02530	strong similarity to hypothetical protein EAA60542.1 - Aspergillus nidulans	-0.6
An08g00670	similarity to hypothetical membrane protein YGR101w - Saccharomyces cerevisiae	-0.6
An07g09360	strong similarity to maleylacetate reductase MacA - Rhodococcus opacus	-0.6
An06g01990	weak similarity to integral membrane protein PTH11 - Magnaporthe grisea	-0.6
An04g07060	strong similarity to peptide ABC transporter protein Mdl1 - Saccharomyces cerevisiae	-0.6
An04g06890	similarity to 72-kD protein of the signal recognition particle SRP72 - Canis lupus	-0.6
An04g00220	strong similarity to methionine-N-acetyltransferase Nat2 - Saccharomyces cerevisiae	-0.6
An02g08010	similarity to hypothetical serine-rich protein SPBC215.13 - Schizosaccharomyces pombe	-0.6
An02g07870	similarity to prolyl-4-hydroxylase-alpha - Drosophila melanogaster	-0.6
An02g06860	strong similarity to glutamate decarboxylase 1 - Felis silvestris catus	-0.6
An02g06150	strong similarity to branched-chain amino acid aminotransferase Bat2 - Saccharomyces cerevisiae	-0.6
An02g05180	hypothetical protein	-0.6
An02g01040	similarity to hypothetical protein EAA71860.1 - Gibberella zeae	-0.6
An01g08570	strong similarity to thioredoxin reductase TrxB - Penicillium chrysogenum	-0.6
An08g04080	strong similarity to mitochondrial processing peptidase alpha chain MPP - Neurospora crassa	-0.6
An18g05690	strong similarity to hypothetical protein YDR372c - Saccharomyces cerevisiae	-0.55
An18g04400	strong similarity to GTP-binding protein DRG - Xenopus laevis	-0.55

An18g03580	similarity to hypothetical protein SPBC543.08 - Schizosaccharomyces pombe	-0.55
An18g03300	strong similarity to cytosolic phenylalanine--tRNA ligase alpha subunit Frs1 - Saccharomyces cerevisiae	-0.55
An17g01270	strong similarity to mitochodrial ribosomal protein of the large subunit Mrpl7 - Saccharomyces cerevisiae	-0.55
An16g08850	strong similarity to origin recognition complex associated protein p81 - Xenopus laevis	-0.55
An16g07840	similarity to M protein emm55 - Streptococcus pyogenes	-0.55
An16g06120	strong similarity to glycosylphosphatidylinositol-anchored beta(1-3)glucanosyltransferase gel3 - Aspergillus fumigatus	-0.55
An16g06010	similarity to phosphoglycerate mutase pgm - Zymomonas mobilis	-0.55
An16g05880	strong similarity to neutral amino acid permease mtr - Neurospora crassa	-0.55
An16g05470	weak similarity to hypothetical RNA-binding protein - Schizosaccharomyces pombe	-0.55
An16g03870	weak similarity to transcription termination factor Rho - Micrococcus luteus	-0.55
An16g02520	strong similarity to threonine synthase Thr4 - Saccharomyces cerevisiae	-0.55
An15g03220	strong similarity to member of the subfamily of yeast glutaredoxins Grx4 - Saccharomyces cerevisiae	-0.55
An15g02420	similarity to eukaryotic translation initiation factor eIF2B subunit 3 - Homo sapiens	-0.55
An15g01020	strong similarity to cardiolipin synthase Crd1 - Saccharomyces cerevisiae	-0.55
An15g00930	strong similarity to chaperone for ubiquinol-cytochrome c reductase Abc1 - Saccharomyces cerevisiae	-0.55
An15g00550	strong similarity to hypothetical protein YBR101c - Saccharomyces cerevisiae	-0.55
An15g00420	strong similarity to mitochondrial leucine--tRNA ligase leu-5 - Neurospora crassa	-0.55
An14g07200	strong similarity to catalase C catC - Aspergillus nidulans	-0.55
An14g07130	strong similarity to neutral amino acid permease mtr - Neurospora crassa	-0.55
An14g05750	strong similarity to 12-oxo-phytodienoate reductase OPR3 - Arabidopsis thaliana	-0.55
An14g05000	strong similarity to protein Ria1 - Saccharomyces cerevisiae	-0.55
An14g03330	similarity to hypothetical protein SCF12.05 - Streptomyces coelicolor	-0.55
An14g00550	strong similarity to hypothetical protein YBL036c - Saccharomyces cerevisiae	-0.55
An14g00310	strong similarity to beta-succinyl CoA synthase precursor scsB - Neocallimastix frontalis	-0.55
An13g03510	hypothetical protein	-0.55
An13g01000	weak similarity to death associated protein DAP3 from patent WO9839429-A2 - Homo sapiens	-0.55
An13g00440	strong similarity to uridine-monophosphate kinase Ura6 - Saccharomyces cerevisiae	-0.55
An12g07580	strong similarity to acetylglutamate kinase/N-acetyl-gamma-glutamyl-phosphate reductase precursor arg-6 - Neurospora crassa	-0.55
An12g04750	strong similarity to prohibitin Phb2 - Saccharomyces cerevisiae	-0.55
An12g04660	strong similarity to thiamin-phosphate pyrophosphorylase/hydroxyethylthiazole kinase Thi6 - Saccharomyces cerevisiae	-0.55
An12g02520	strong similarity to clavulanic acid dehydrogenase from patent WO9503416-A - Streptomyces clavuligerus	-0.55
An12g00130	strong similarity to mRNA processing protein of cytochrome c oxidase Mss51 - Saccharomyces cerevisiae	-0.55
An11g09510	strong similarity to aspartate-semialdehyde dehydrogenase Hom2 - Saccharomyces cerevisiae	-0.55
An11g06790	weak similarity to DEAH-box RNA helicase Mut6 - Chlamydomonas reinhardtii	-0.55
An11g04520	strong similarity to hypothetical protein B23I11.70 - Neurospora crassa	-0.55
An11g03730	weak similarity to integral membrane protein PTH11 from patent WO9913094-A2 - Magnaporthe grisea	-0.55
An11g00090	similarity to hypothetical membrane protein pth - Blumeria graminis	-0.55
An10g00360	strong similarity to nuclear pore-associated DEAD-box protein Dbp5 - Saccharomyces cerevisiae	-0.55
An09g03500	benzoate 4-monooxygenase cytochrome P450 53 bphA - Aspergillus niger	-0.55
An09g03030	strong similarity to glutathione synthase Gsh2 - Saccharomyces cerevisiae [putative sequencing error]	-0.55

An08g10340	strong similarity to 3,4-dihydroxy-2-butanone 4-phosphate synthase Rib3 - Saccharomyces cerevisiae	-0.55
An08g06080	anthranilate synthase multifunctional protein trpC - Aspergillus niger	-0.55
An08g02800	strong similarity to nuclear WD protein PRL1 - Arabidopsis thaliana	-0.55
An07g09550	strong similarity to mitochondrial ribosomal protein Nam9 - Saccharomyces cerevisiae	-0.55
An07g09120	strong similarity to precursor of methionyl aminopeptidase Map1 - Saccharomyces cerevisiae	-0.55
An07g07420	strong similarity to splicing factor PRP8 - Homo sapiens	-0.55
An07g07300	strong similarity to prostaglandin f(2alpha) synthase TbPGFS - Trypanosoma brucei	-0.55
An07g05620	strong similarity to transcription elongation factor Rtf1 - Saccharomyces cerevisiae	-0.55
An07g05510	strong similarity to ferric reductase Fre2 - Saccharomyces cerevisiae	-0.55
An07g02770	strong similarity to hypothetical protein YKL195w - Saccharomyces cerevisiae	-0.55
An07g01580	strong similarity to nuclear import protein Mtr10 - Saccharomyces cerevisiae	-0.55
An05g01290	strong similarity to hexose transporter Hxt3 - Saccharomyces cerevisiae	-0.55
An04g07120	similarity to protein FRM2 involved in fatty acid regulation - Saccharomyces cerevisiae	-0.55
An04g07080	similarity to hypothetical protein SPBC2G5.01 - Schizosaccharomyces pombe	-0.55
An04g06490	strong similarity to 3,4-dihydroxy-5-hexaprenylbenzoate methyltransferase - Rattus norvegicus	-0.55
An04g05650	strong similarity to hypothetical protein SPBC1709.13c - Schizosaccharomyces pombe	-0.55
An04g03060	strong similarity to mitochondrial tyrosine--tRNA ligase CYT-18 - Neurospora crassa	-0.55
An04g02690	strong similarity to alcohol dehydrogenase ADH from patent EP0845532-A - Unclassified organism	-0.55
An04g02580	strong similarity to threonine deaminase ILV - Arxula adeninivorans	-0.55
An04g02460	strong similarity to heart muscle protein mitofilin HMP - Homo sapiens	-0.55
An04g02320	strong similarity to metalloprotease 1 MP1 - Homo sapiens	-0.55
An04g01460	strong similarity to acyl-CoA thioesterase act48.1 - Mus musculus	-0.55
An04g01340	strong similarity to asparagine synthase Asn1 - Saccharomyces cerevisiae	-0.55
An04g01160	strong similarity to hypothetical ABC transporter SPBC15C4.02 - Schizosaccharomyces pombe	-0.55
An03g03640	strong similarity to mitochondrial sulfide dehydrogenase (coenzyme Q2) SPBC2G5.06c - Schizosaccharomyces pombe	-0.55
An03g03530	similarity to protein fragment SEQ ID NO:9681 from patent EP1033405-A2 - Arabidopsis thaliana	-0.55
An03g03500	strong similarity to hypothetical protein CAD36989.1 - Neurospora crassa	-0.55
An03g03470	similarity to deoxyribodipyrimidine photolyase phrA - Escherichia coli	-0.55
An03g03000	similarity to molybdopterin-converting factor activator chlN - Escherichia coli	-0.55
An03g01310	strong similarity to CTP synthase Ura7 - Saccharomyces cerevisiae	-0.55
An02g12420	strong similarity to trifunctional C1-tetrahydrofolate synthase Ade3 - Saccharomyces cerevisiae [putative frameshift]	-0.55
An02g11970	strong similarity to 4-nitrophenylphosphatase pho13p - Schizosaccharomyces pombe	-0.55
An02g11800	strong similarity to hypothetical membrane protein YPR125w - Saccharomyces cerevisiae	-0.55
An02g07880	strong similarity to quinate transport protein qutD - Aspergillus nidulans	-0.55
An02g06850	similarity to hypothetical membrane protein YLR077w - Saccharomyces cerevisiae	-0.55
An02g04880	strong similarity to arginine--tRNA ligase Msr1 - Saccharomyces cerevisiae	-0.55
An02g02380	strong similarity to hypothetical suppressor of deletion of TFIIS Ssm1 - Saccharomyces cerevisiae	-0.55
An02g01540	strong similarity to ferredoxin-NADP+ reductase FNR - Homo sapiens	-0.55
An01g13920	strong similarity to adenylosuccinate synthase ade2p - Schizosaccharomyces pombe	-0.55
An01g13830	strong similarity to GTP-binding protein cgtA - Caulobacter crescentus	-0.55

An01g10990	strong similarity to mitochondrial threonine--tRNA ligase Mst1 - Saccharomyces cerevisiae	-0.55
An01g06110	strong similarity to glycerol-3-phosphate dehydrogenase (NAD+) precursor Gpd1 - Saccharomyces cerevisiae	-0.55
An01g05400	weak similarity to hypothetical protein 6R55.1 - Caenorhabditis elegans	-0.55
An01g00550	strong similarity to allergen rAsp f 4 - Aspergillus fumigatus	-0.55
An18g04570	strong similarity to cleavage/polyadenylation factor IA subunit Clp1 - Saccharomyces cerevisiae	-0.55
An16g08840	strong similarity to hypothetical protein CAE76483.1 - Neurospora crassa	-0.55
An14g04260	strong similarity to hypothetical protein PA4181 - Pseudomonas aeruginosa	-0.55
An14g00220	strong similarity to hypothetical protein SPAC17C9.06 - Schizosaccharomyces pombe	-0.55
An12g09280	similarity to hypothetical protein encoded by An01g02930 - Aspergillus niger	-0.55
An04g03120	strong similarity to assembly factor of cytochrome c oxidase Cox10 - Saccharomyces cerevisiae	-0.55
An02g05640	strong similarity to hypothetical protein SPAC11D3.03c - Schizosaccharomyces pombe	-0.55
An02g03760	strong similarity to precursor of endopeptidase La-like enzyme Pim1 - Saccharomyces cerevisiae	-0.55
An01g08830	similarity to hypothetical protein CAD70783.1 - Neurospora crassa	-0.55
An01g03110	similarity to ribosomal protein of the small subunit Rsm7 - Saccharomyces cerevisiae	-0.55
An18g06260	similarity to eukaryotic translation initiation factor eIF3 subunit p35 - Homo sapiens	-0.5
An18g06010	similarity to ribosomal protein of the large subunit Yml40 - Saccharomyces cerevisiae	-0.5
An18g05740	similarity to ARF binding protein GGA2 - Homo sapiens	-0.5
An18g05120	strong similarity to subunit of succinyl-CoA:benzylsuccinate CoA-transferase bbsF - Thauera aromatica	-0.5
An18g04650	strong similarity to guanine nucleotide exchange factor eIF-2B 34 kDa alpha subunit Gcn3 - Saccharomyces cerevisiae	-0.5
An18g03770	strong similarity to ATPase Mot1 - Saccharomyces cerevisiae	-0.5
An18g03120	strong similarity to ATP dependent RNA helicase and pre-mRNA splicing factor Prp43 - Saccharomyces cerevisiae	-0.5
An18g03060	similarity to leucine carboxyl methyltransferase LCMT - Homo sapiens	-0.5
An18g02970	strong similarity to hydroxymethylbilane synthase Hem3 - Saccharomyces cerevisiae	-0.5
An18g02850	strong similarity to mtRNA splice defect-suppressing mitochondrial carrier Mrs3 - Saccharomyces cerevisiae	-0.5
An18g02350	strong similarity to protein phosphatase X PPX - Homo sapiens	-0.5
An18g02140	strong similarity to spi1-GTPase binding protein sbp1p - Schizosaccharomyces pombe	-0.5
An18g01970	strong similarity to pantoate--beta-alanine ligase panC - Corynebacterium glutamicum	-0.5
An18g01610	strong similarity to RNA polymerase II suppressor protein Srb4 - Saccharomyces cerevisiae	-0.5
An18g01400	strong similarity to monocarboxylate transporter 3 MCT3 - Rattus norvegicus	-0.5
An17g01950		-0.5
An17g01890		-0.5
An17g01080	weak similarity to member of the trichothecene gene cluster TRI7 - Fusarium sporotrichioides	-0.5
An17g00890	strong similarity to phosphoprotein phosphatase PPT - Rattus norvegicus	-0.5
An17g00880	strong similarity to endometrium tumour EST encoded protein 137 from patent DE19817948-A1 - Homo sapiens	-0.5
An17g00210	strong similarity to hypothetical oxidoreductase CAB46711.1 - Schizosaccharomyces pombe	-0.5
An16g08390	similarity to sequence 4 from patent WO0063399 - Aureobasidium pullulans	-0.5
An16g08380	strong similarity to U4/U6 snRNP 52kD protein Prp4 - Saccharomyces cerevisiae	-0.5

An16g08020	strong similarity to valine--tRNA ligase cyt-20 - Neurospora crassa [putative frameshift]	-0.5
An16g07400	strong similarity to NAD-dependent 5,10-methylenetetrahydrafolate dehydrogenase Mtd1 - Saccharomyces cerevisiae	-0.5
An16g05430	strong similarity to hypothetical protein EAA58013.1 - Aspergillus nidulans	-0.5
An16g05090	strong similarity to endonuclease SceI 75 kDa subunit Ens1 - Saccharomyces cerevisiae	-0.5
An16g03950	strong similarity to amadoriase II - Aspergillus fumigatus	-0.5
An16g03590	strong similarity to phosphatidylinositol-phosphatidylcholine transfer protein Sec14 - Saccharomyces cerevisiae	-0.5
An16g03100	strong similarity to TCP-1-containing cytosolic chaperonin zeta subunit CctZ - Mus musculus	-0.5
An16g02470	strong similarity to FAD-linked sulfhydryl oxidase Erv1 - Saccharomyces cerevisiae	-0.5
An16g02200	similarity to hypothetical protein YBR246w - Saccharomyces cerevisiae	-0.5
An16g01560	strong similarity to protein Gcd10 - Saccharomyces cerevisiae	-0.5
An15g06360	similarity to multifunctional glutamine-proline--tRNA ligase Aats-glupro - Drosophila melanogaster	-0.5
An15g03670	similarity to hypothetical C2H2-type zinc-finger protein - Schizosaccharomyces pombe	-0.5
An15g03200	strong similarity to mitochondrial uncoupling protein MUP1 from patent WO200061614-A2 - Homo sapiens	-0.5
An15g02660	strong similarity to essential protein SEQ ID NO:22 from patent WO2003076464-A2 - Aspergillus fumigatus	-0.5
An15g01290	strong similarity to protein D123 - Homo sapiens	-0.5
An15g00890	strong similarity to hypothetical regulator protein Rms1 - Saccharomyces cerevisiae	-0.5
An15g00770	strong similarity to mitochondrial ribosomal protein of the small subunit S9 - Saccharomyces cerevisiae	-0.5
An15g00190	strong similarity to mitochondrial import receptor MOM38 - Neurospora crassa	-0.5
An14g06980	strong similarity to delta-12 fatty acid desaturase - Mortierella alpina	-0.5
An14g06780	strong similarity to heat-shock protein Hsp40 - Homo sapiens	-0.5
An14g06550	strong similarity to hypothetical membrane protein YNL011c - Saccharomyces cerevisiae	-0.5
An14g06320	strong similarity to mitochondrial protease Imp1 - Saccharomyces cerevisiae	-0.5
An14g06310	strong similarity to mitochondrial ribosomal protein of the large subunit Yml35 - Saccharomyces cerevisiae	-0.5
An14g06290	strong similarity to 3-hydroxyisobutyryl-coenzyme A hydrolase from patent WO9851782-A2 - Homo sapiens	-0.5
An14g06190	similarity to translation initiation factor eIF2B 71 kDa delta subunit Gcd2 - Saccharomyces cerevisiae	-0.5
An14g04980	strong similarity to splicing protein Prp19 - Saccharomyces cerevisiae	-0.5
An14g04790	strong similarity to peptidyl-prolyl cis-trans isomerase SPAC21E11.05C - Schizosaccharomyces pombe	-0.5
An14g04740	weak similarity to hypothetical protein SPBC18H10.17c - Schizosaccharomyces pombe	-0.5
An14g03780	strong similarity to mitochondrial RNA helicase Suv3 - Saccharomyces cerevisiae	-0.5
An14g03740	strong similarity to hypothetical protein SPCC14G10.02 - Schizosaccharomyces pombe	-0.5
An14g03030	similarity to spermidine/spermine N(1)-acetyltransferase SAT - Homo sapiens [putative frameshift]	-0.5
An14g01560	strong similarity to zuotin Zuo1 - Saccharomyces cerevisiae	-0.5
An14g01030	strong similarity to translation initiation factor eIF-3 subunit - Homo sapiens [truncated ORF]	-0.5
An14g00630	strong similarity to salt tolerance protein Met22 - Saccharomyces cerevisiae	-0.5
An13g02710	weak similarity to hypothetical protein YIL064w - Saccharomyces cerevisiae	-0.5
An13g01530	weak similarity to hypothetical protein ycnE - Bacillus subtilis	-0.5

An13g00600	weak similarity to hypothetical protein Rv3439c - Mycobacterium tuberculosis [truncated ORF]	-0.5
An12g07950	strong similarity to mitochondrial RNA splicing protein Mrs2 - Saccharomyces cerevisiae	-0.5
An12g07870	strong similarity to cytosolic aspartate transaminase Aat2 - Saccharomyces cerevisiae	-0.5
An12g07100	strong similarity to cucumopine synthase cus - Agrobacterium rhizogenes	-0.5
An12g05510	strong similarity to triacetylfusarinine C transporter Taf1 - Saccharomyces cerevisiae	-0.5
An12g03850	similarity to DEAD box RNA helicase CHR1 - Candida albicans [putative frameshift]	-0.5
An12g00760	strong similarity to hypothetical nicotinate phosphoribosyltransferase Npt1 - Saccharomyces cerevisiae	-0.5
An11g10470	strong similarity to glutaryl-CoA dehydrogenase GCHD - Homo sapiens	-0.5
An11g10370	strong similarity to trans-pentaprenyltranstransferase Coq1 - Saccharomyces cerevisiae	-0.5
An11g10140	strong similarity to NADH:ubiquinone reductase intermediate-associated protein CIA30 - Neurospora crassa	-0.5
An11g10070	weak similarity to hypothetical protein SPAC13G7.11 - Schizosaccharomyces pombe	-0.5
An11g09830	weak similarity to hypothetical protein B0280.2 - Caenorhabditis elegans	-0.5
An11g09790	strong similarity to sulfate adenylyltransferase sC - Aspergillus nidulans	-0.5
An11g07970	strong similarity to hypothetical protein SPBC16H5.12c - Schizosaccharomyces pombe	-0.5
An11g07650	strong similarity to antioxidant protein AOP2 - Mus musculus	-0.5
An11g06810	strong similarity to mitochondrial ribosomal protein of the large subunit L6 - Saccharomyces cerevisiae	-0.5
An11g06230	similarity to oxidoreductase from patent WO0100844 - Corynebacterium glutamicum	-0.5
An11g05840	strong similarity to hypothetical short chain dehydrogenase L3377.10 - Leishmania major	-0.5
An11g05810	similarity to 2,4-dihydroxyacetophenone dioxygenase dad - Alcaligenes sp.	-0.5
An11g04850	hypothetical protein	-0.5
An11g03170	similarity to hypothetical phosphoglycerate mutase SPAC5H10.03 - Schizosaccharomyces pombe	-0.5
An11g02830	similarity to resistance to o-dinitrobenzene protein Rod1 - Saccharomyces cerevisiae [truncated ORF]	-0.5
An11g02670	strong similarity to hypothetical protein SPBC1703.02 - Schizosaccharomyces pombe	-0.5
An11g02400	strong similarity to cytosolic lysine--tRNA ligase Krs1 - Saccharomyces cerevisiae	-0.5
An11g02360	strong similarity to T-complex protein 1 epsilon subunit CCT5 - Homo sapiens	-0.5
An11g02030	similarity to hypothetical protein T23E23.20 - Arabidopsis thaliana	-0.5
An11g01580	similarity to protoporphyrinogen oxidase PPO - Mus musculus	-0.5
An11g00770	strong similarity to SUMO-1 activating enzyme subunit 2 SAE2 - Homo sapiens	-0.5
An11g00480	strong similarity to spindle assembly checkpoint protein sldA - Aspergillus nidulans	-0.5
An09g06680	citrate synthase citA - Aspergillus niger	-0.5
An09g06580	strong similarity to ras-GTPase-activating protein SH3-domain binding protein G3BP - Mus musculus	-0.5
An09g06070	strong similarity to subunit of transcription initiation factor TFIID p80 - Xenopus laevis	-0.5
An09g05980	similarity to ribosomal protein of the large subunit L1 rplA - Escherichia coli	-0.5
An09g05970	strong similarity to cytosolic glutamate--tRNA ligase - Arabidopsis thaliana	-0.5
An09g05840	strong similarity to thymidylate kinase CDC8 - Homo sapiens	-0.5
An09g05100	similarity to Impact - Mus musculus	-0.5
An09g03950	strong similarity to cytosolic tryptophan--tRNA ligase Wrs1 - Saccharomyces cerevisiae	-0.5
An08g10610	strong similarity to hypothetical protein EAA59931.1 - Aspergillus nidulans	-0.5

An08g10500	strong similarity to hypothetical protein EAA62682.1 - Aspergillus nidulans	-0.5
An08g07000	similarity to hypothetical protein CAF32094.1/AfA19D12.030 - Aspergillus fumigatus	-0.5
An08g06180	similarity to 6-hydroxy-D-nicotine oxidase 6-HDNO - Arthrobacter oxidans	-0.5
An08g05580	strong similarity to precursor of isocitrate dehydrogenase (NAD+) chain Idh2 - Saccharomyces cerevisiae	-0.5
An08g05500	strong similarity to mitochondrial ribosomal protein of the large subunit Yml3 - Saccharomyces cerevisiae	-0.5
An08g05060	strong similarity to hypothetical protein encoded by AfA14E5.29 - Aspergillus fumigatus	-0.5
An08g04670	strong similarity to hypothetical sugar transporter encoded by DRA0271 - Deinococcus radiodurans	-0.5
An08g04260	similarity to hypothetical protein YNL191w - Saccharomyces cerevisiae	-0.5
An08g04240	strong similarity to alternative NADH:ubiquinone reductase NDH2 - Yarrowia lipolytica	-0.5
An08g03920	strong similarity to hypothetical protein CAE47894.1 - Aspergillus fumigatus	-0.5
An08g02990	strong similarity to adenylylsulfate kinase Met14 - Saccharomyces cerevisiae	-0.5
An08g01920	similarity to acetate regulatory DNA binding protein facB - Aspergillus nidulans	-0.5
An08g01790	strong similarity to eukaryotic initiation factor 3H1 subunit TIF3H1 - Arabidopsis thaliana	-0.5
An08g01740	strong similarity to uronate dehydrogenase from patent DE19604798-A1 - Saccharomyces cerevisiae	-0.5
An08g01650	similarity to mitochondrial import protein Mpi1 - Saccharomyces cerevisiae	-0.5
An08g00980	strong similarity to translation initiation factor eIF2B guanine nucleotide exchange factor 43 kDa subunit Gcd7 - Saccharomyces cerevisiae	-0.5
An08g00730	similarity to transitional endoplasmic reticulum ATPase - Rattus norvegicus	-0.5
An08g00500	similarity to hypothetical protein CAD70990.1 - Neurospora crassa	-0.5
An07g10040	strong similarity to coproporphyrinogen oxidase III Hem13 - Saccharomyces cerevisiae	-0.5
An07g10010	strong similarity to mitochondrial carrier protein Yhm1 - Saccharomyces cerevisiae	-0.5
An07g09340	similarity to hypothetical protein YPR169w - Saccharomyces cerevisiae	-0.5
An07g09110	similarity to monooxygenase VioC - Chromobacterium violaceum	-0.5
An07g08430	strong similarity to mitochondrial preprotein import complex subunit metaxin MTX - Homo sapiens [truncated ORF]	-0.5
An07g07880	similarity to ORFX ORF2689 polypeptide sequence SEQ ID NO:5378 from patent WO200058473-A2 - Homo sapiens	-0.5
An07g07000	strong similarity to mitochondrial m-AAA protease subunit Yta12 - Saccharomyces cerevisiae	-0.5
An07g06630	hypothetical protein	-0.5
An07g06000	strong similarity to cytosolic aspartate--tRNA ligase Dps1 - Saccharomyces cerevisiae	-0.5
An07g05290	similarity to hypothetical mitochondrial ribosomal protein - Schizosaccharomyces pombe	-0.5
An07g03940	strong similarity to zinc-finger transcription factor amdA - Aspergillus nidulans	-0.5
An07g03150	strong similarity to nuclear tRNA export receptor exportin-t - Homo sapiens	-0.5
An07g03080	hypothetical protein	-0.5
An06g01820	similarity to hypothetical ribosomal protein B13I18.70 - Neurospora crassa	-0.5
An06g01780	strong similarity to (beta)-transducin like protein TBL1 - Homo sapiens	-0.5
An06g01540	strong similarity to serine C-palmitoyltransferase chain Lcb1 - Saccharomyces cerevisiae	-0.5
An06g01520	similarity to hypothetical protein SPBC24C6.10c - Schizosaccharomyces pombe	-0.5
An06g00280	strong similarity to hypothetical protein YBR096w - Saccharomyces cerevisiae	-0.5
An05g00220	strong similarity to L-ornithine N5-hydroxylase psbA - Pseudomonas sp.	-0.5
An04g08210	strong similarity to pyruvate water dikinase ppsA - Escherichia coli [putative sequencing error]	-0.5
An04g06210	strong similarity to homocitrate synthase lys1 - Penicillium chrysogenum	-0.5
An04g05420	strong similarity to alpha-aminoadipate reductase large subunit lys2 - Penicillium chrysogenum	-0.5

An04g03110	strong similarity to mitochondrial aspartate--tRNA ligase Msd1 - Saccharomyces cerevisiae	-0.5
An04g02270	strong similarity to hypothetical protein SPAC577.03c - Schizosaccharomyces pombe	-0.5
An04g02230	strong similarity to mitochondrial methionine--tRNA ligase MetRS - Candida albicans	-0.5
An04g01940	strong similarity to translation initiation factor eIF2 gamma chain Gcd11 - Saccharomyces cerevisiae	-0.5
An04g01650	strong similarity to hypothetical protein B23I11.250 - Neurospora crassa	-0.5
An04g01620	similarity to transcription factor ZFM1 - Homo sapiens	-0.5
An04g00490	strong similarity to regulator subunit of the casein kinase II Ckb1 - Saccharomyces cerevisiae	-0.5
An04g00390	strong similarity to chromatin-remodeling complex protein Sfh1 - Saccharomyces cerevisiae	-0.5
An04g00210	strong similarity to ribosomal protein of the large subunit Yml9 - Saccharomyces cerevisiae	-0.5
An04g00200	strong similarity to hypothetical membrane protein YIL157c - Saccharomyces cerevisiae	-0.5
An03g05170	similarity to sterol regulatory element binding protein-1 srebp-1 - Homo sapiens	-0.5
An03g05080	similarity to hypothetical protein YKL069w - Saccharomyces cerevisiae	-0.5
An03g04790	strong similarity to mitochondrial outer membrane protein Tom70 - Podospora anserina	-0.5
An03g04660	weak similarity to hypothetical protein YGR165w - Saccharomyces cerevisiae	-0.5
An03g04330	strong similarity to glutamate N-acetyltransferase Arg7 - Saccharomyces cerevisiae	-0.5
An03g04280	strong similarity to pyridoxine synthesis component pyroA - Aspergillus nidulans	-0.5
An03g03710	strong similarity to quinate 5-dehydrogenase qutB - Aspergillus nidulans	-0.5
An03g03660	strong similarity to glutathione reductase Glr1 - Saccharomyces cerevisiae	-0.5
An03g03460	strong similarity to L-galactonolactone oxidase Alo - Saccharomyces cerevisiae	-0.5
An03g02810	hypothetical protein	-0.5
An03g02620	strong similarity to protein fragment SEQ ID NO:34719 from patent EP1033405-A2 - Arabidopsis thaliana	-0.5
An02g14750	strong similarity to actin-related protein - Homo sapiens	-0.5
An02g14240	strong similarity to mRNA guanylyltransferase - Saccharomyces cerevisiae	-0.5
An02g14080	strong similarity to asparagine--tRNA ligase ASNS - Thermus aquaticus	-0.5
An02g13360	strong similarity to assembly factor of cytochrome c oxidase Sco1 - Saccharomyces cerevisiae	-0.5
An02g12920	strong similarity to hypothetical protein SPAC10F6.14c - Schizosaccharomyces pombe	-0.5
An02g12870	hypothetical protein	-0.5
An02g12750	strong similarity to cytoskeleton specific chaperonin subunit Cct4 - Saccharomyces cerevisiae	-0.5
An02g12620	strong similarity to copper metallochaperone Cox17 - Saccharomyces cerevisiae [putative sequencing error]	-0.5
An02g12610	strong similarity to Ran-GTPase-activating protein 1 rna1p - Schizosaccharomyces pombe	-0.5
An02g12320	strong similarity to translation initiation factor eIF-2 beta chain Sui3 - Saccharomyces cerevisiae	-0.5
An02g11570	similarity to mitochondrial ribosomal protein of the large subunit Yml49 - Saccharomyces cerevisiae	-0.5
An02g11300	similarity to mitochondrial ribosomal protein of the large subunit Yml37 - Saccharomyces cerevisiae	-0.5
An02g10670	strong similarity to protein D-lactate dehydrogenase YEL071w - Saccharomyces cerevisiae	-0.5
An02g10450	strong similarity to GTP-binding protein Vps1 - Saccharomyces cerevisiae	-0.5
An02g10390	strong similarity to eukaryotic translation initiation factor eIF-4A II - Mus musculus	-0.5
An02g09470	strong similarity to hypothetical Ca2+-transporting ATPase Spf1 - Saccharomyces cerevisiae [truncated ORF]	-0.5

An02g09370	similarity to translation initiation factor eIF-2 alpha chain EIF2A - Homo sapiens [truncated ORF]	-0.5
An02g09190	strong similarity to mitochondrial ribosomal protein of the small subunit S15 - Saccharomyces cerevisiae	-0.5
An02g08920	strong similarity to TCP1-related protein Cct3 - Saccharomyces cerevisiae	-0.5
An02g08820	strong similarity to hypothetical protein SPBC31F10.16 - Schizosaccharomyces pombe	-0.5
An02g07430	strong similarity to homoserine dehydrogenase YJR139c - Saccharomyces cerevisiae	-0.5
An02g07120	strong similarity to viral integration site protein int-6/EIF-3 P48 - Mus musculus	-0.5
An02g06530	strong similarity to protein required for dispersion of mitochondria cluA - Dictyostelium discoideum	-0.5
An02g06390	strong similarity to cytoplasmic ribosomal protein of the small subunit Rp30 - Saccharomyces cerevisiae	-0.5
An02g05880	strong similarity to 29.9 kD subunit of NADH:ubiquinone reductase - Neurospora crassa	-0.5
An02g05870	strong similarity to coatomer beta subunit copB2 - Homo sapiens [putative frameshift]	-0.5
An02g04830	strong similarity to extragenic suppressor of the bimD6 mutation sudD - Aspergillus nidulans	-0.5
An02g03850	similarity to small nuclear ribonucleoprotein G snRPG - Homo sapiens	-0.5
An02g03620	strong similarity to protein involved in cephalosporin C biosynthesis from patent JP09009966-A - Acremonium chrysogenum	-0.5
An02g03510	similarity to arylalkylamine N-acetyltransferase AANAT1 - Esox lucius	-0.5
An02g03290	strong similarity to hypothetical protein SPCC4G3.11 - Schizosaccharomyces pombe	-0.5
An02g03180	strong similarity to DNA-directed RNA polymerase II accessory protein Cdc73 - Saccharomyces cerevisiae	-0.5
An02g02980	strong similarity to protein influencing Itr1 expression Die2 - Saccharomyces cerevisiae	-0.5
An02g02910	strong similarity to orotate reductase pyrE - Aspergillus nidulans	-0.5
An02g02900	strong similarity to RNA polymerase II-associated factor Paf1 - Saccharomyces cerevisiae	-0.5
An02g02320	similarity to RNA polymerase II transcription factor Upc2 - Saccharomyces cerevisiae	-0.5
An02g02170	strong similarity to tryptophan synthase Trp5 - Saccharomyces cerevisiae	-0.5
An02g01740	strong similarity to mitochondrial phenylalanine--tRNA ligase alpha subunit Msf1 - Saccharomyces cerevisiae	-0.5
An02g01630	similarity to spastin protein Spast - Homo sapiens	-0.5
An02g00890	strong similarity to phosphoserine transaminase Ser1 - Saccharomyces cerevisiae	-0.5
An01g14680	similarity to hypothetical protein SPCC576.01c - Schizosaccharomyces pombe	-0.5
An01g14110	strong similarity to phosphatidylserine decarboxylase 1 Psd1 - Saccharomyces cerevisiae	-0.5
An01g12180	strong similarity to tRNA (cytosine-5-)-methyltransferase Ncl1 - Saccharomyces cerevisiae	-0.5
An01g12020	strong similarity to hypothetical protein encoded by An04g09400 - Aspergillus niger	-0.5
An01g11960	similarity to brefeldin A resistance protein Bfr1 - Saccharomyces cerevisiae	-0.5
An01g11920	similarity to hypothetical protein SPBC1A4.06c - Schizosaccharomyces pombe	-0.5
An01g11650	strong similarity to formyltetrahydrofolate deformylase purU - Corynebacterium sp.	-0.5
An01g10900	similarity to hypothetical la costa protein lcs - Drosophila melanogaster	-0.5
An01g10570	similarity to hypothetical protein EAA65998.1 - Aspergillus nidulans	-0.5
An01g10340	similarity to hypothetical fatty acid elongation protein Sur4 - Saccharomyces cerevisiae	-0.5
An01g10070	strong similarity to signal recognition particle chain Sec65 - Saccharomyces cerevisiae	-0.5

An01g09910	strong similarity to GPI-anchor biosynthesis protein Pig-a - Saccharomyces cerevisiae	-0.5
An01g09550	similarity to hypothetical dihydrolipoamide dehydrogenase Rv2713 - Mycobacterium tuberculosis	-0.5
An01g09500	strong similarity to cytosolic threonine--tRNA ligase Ths1 - Saccharomyces cerevisiae	-0.5
An01g09100	strong similarity to actin-related protein arpA - Aspergillus oryzae	-0.5
An01g08860	strong similarity to hypothetical protein 12F11.240 - Neurospora crassa	-0.5
An01g08490	strong similarity to histidine--tRNA ligase Hts1 - Saccharomyces cerevisiae	-0.5
An01g08280	strong similarity to hypothetical protein CAC28582.1 - Neurospora crassa	-0.5
An01g08090	strong similarity to aminoimidazole ribonucleotide carboxylase ADE2 - Candida glabrata	-0.5
An01g07500	strong similarity to hypothetical protein CAF06125.1 - Neurospora crassa	-0.5
An01g07460	similarity to beta transducin-like protein het-e1 - Podospora anserina	-0.5
An01g07430	strong similarity to mitochondrial ribosomal protein of the large subunit L2 - Saccharomyces cerevisiae	-0.5
An01g07200	similarity to N-acetylglucosaminyltransferases chain p110 - Rattus norvegicus	-0.5
An01g07190	strong similarity to component of the translocase of mitochondrial inner membrane Tim54 - Saccharomyces cerevisiae	-0.5
An01g06250	strong similarity to hypothetical zinc-finger protein SPCC1223.01 - Schizosaccharomyces pombe	-0.5
An01g06230	strong similarity to translation initiation factor 3 subunit eIF3 beta - Homo sapiens	-0.5
An01g06210	strong similarity to F-box protein Rcy1 - Saccharomyces cerevisiae	-0.5
An01g05820	strong similarity to homoprotocatechuate operon enzyme hpcE - Escherichia coli	-0.5
An01g05760	strong similarity to ubiquitin fusion degradation protein Ufd1 - Saccharomyces cerevisiae	-0.5
An01g05050	strong similarity to pyridoxal kinase PKH - Homo sapiens	-0.5
An01g04900	strong similarity to hypothetical protein CAC18296.1 - Neurospora crassa	-0.5
An01g04410	strong similarity to N-acetyl transferase NACTH from patent US6017744-A - Homo sapiens	-0.5
An01g04230	strong similarity to hypothetical WD-repeat transcription regulation protein SPCC18.13 - Schizosaccharomyces pombe	-0.5
An01g02800	strong similarity to signal recognition particle 68K protein SRP68 - Canis lupus	-0.5
An01g02000	similarity to aromatic-L-amino-acid decarboxylase DDC - Rattus norvegicus	-0.5
An01g01390	similarity to chromatin remodeling Snf/Swi complex subunit Arp9 - Saccharomyces cerevisiae	-0.5
An18g03560	weak similarity to human protein MLN 51 from patent WO9706256-A2 - Homo sapiens	-0.5
An17g02280	strong similarity to aspartate kinase Hom3 - Saccharomyces cerevisiae	-0.5
An16g04700	similarity to mitochondrial ribosomal protein of the small subunit Mrp51 - Saccharomyces cerevisiae	-0.5
An15g02670	strong similarity to essential protein #22 from patent WO2003076464-A2 - Aspergillus fumigatus [truncated ORF]	-0.5
An15g00600	weak similarity to hypothetical protein CAD11363.1 - Neurospora crassa	-0.5
An14g07060	strong similarity to fatty acid regulation protein Frm2 - Saccharomyces cerevisiae	-0.5
An13g00090	strong similarity to eburicol 14 alpha-demethylase cyp51 - Uncinula necator	-0.5
An12g04940	strong similarity to mitochondrial heat shock protein Hsp60 - Saccharomyces cerevisiae	-0.5
An08g06400	strong similarity to actin-related protein arp2p - Schizosaccharomyces pombe [truncated ORF]	-0.5
An08g04590	similarity to hypothetical scaffold protein CG6385 - Drosophila melanogaster	-0.5
An08g01190	strong similarity to subunit of the major N alpha-acetyltransferase Ard1 - Saccharomyces cerevisiae	-0.5
An04g07340	strong similarity to hypothetical cytoskeleton specific chaperonin subunit Cct8 - Saccharomyces cerevisiae	-0.5
An02g03770	strong similarity to precursor of heat shock protein Mdj1 - Saccharomyces cerevisiae	-0.5
