# Supplementary material for: A Transcriptome Meta-Analysis Proposes Novel Biological Roles for the Antifungal Protein AnAFP in Aspergillus niger
Source: PLoS One. 2016 Nov 11;11(11):e0165755. doi: 10.1371/journal.pone.0165755 (PMC5106034; doi:10.1371/journal.pone.0165755)
Supplement: S4 Table — (PDF) [file pone.0165755.s009.pdf]

S4 Table

Motif	Consensus	TF	Publication
AbaA	CATTCY	AbaA	Andrianopoulos and Timberlake, 1994
AmyR	CGGNNNNNNNNMGG	AmyR	Petersen KL, et al. 1999
AreA	HGATAR	AreA	Peters and Caddick, 1994
AtfB	AGCCS	AtfB	Roze LV, et al. 2011
BRE	MRAGGGR	BrlA	Chang and Timberlake, 1998
Cbf1-A	TCACGTG	Cbf1	Caruso ML, et al. 2002
Cbf1-B	RTCACRTGA	Cbf1	Caruso ML, et al. 2002
CDRE-A	GAGGCTG	CrzA	Yoshimoto, et al. 2002
CDRE-B	GTGGCTG	CrzA	Stathopoulus and Cyert,1997
CDRE-C	GAGGCTC	CrzA	Spielvogel, et al. 2008
CpcA-A	TTGACTCT	CpcA	Hoffmann, et al. 2001
CpcA-B	ATGACTCA	CpcA	Hoffmann, et al. 2001
CreA	SYGGRG	CreA	Kulmburg P, et al. 1994
FarA/FarB	CCTCGG	FarA/FarB	Vogsangnak W, et al. 2010
Fkh1-A	GTAAACAA	AcFKH1	Schmitt EK, et al. 2004
Fkh1-B	GGTAAACAA	AcFKH1	Schmitt EK, et al. 2004
Fkh2-A	GTAAACA	AcFKH1	Schmitt EK, et al. 2004
Fkh2-B	GGTAAACAA	AcFKH1	Schmitt EK, et al. 2004
Flo8p	TTTGCNGCAAA	SomA	Lin CJ, et al. 2015
GARE	CCTCGG	GARE	Niu J, et al. 2015
Gcn4p-A	TGASTCA	CpcA	Wanke C, et al. 1997
Gcn4p-B	ARTGACTCW	CpcA	Wanke C, et al. 1997
HacA-A	CANRNTGKCCT	HacA	Mulder, et al. 2006
HacA-B	CANNTGKCCT	HacA	Mulder, et al. 2006
HAP	CCAAT	HAP	Kato, et al. 1998
LeuB	CCGNNNNCGG	LeuB	Downes DJ, et al. 2013
MARE-A	TGCTGAC	L-Maf	Yoshida, et al. 2005
MARE-B	TGCTGACNTCAGCA	L-Maf	Yoshida, et al. 2005
MeaB	TTGCACCAT	MeaB	Wong KH, et al. 2007
PacC	GCCARG	PacC	Espeso and Penalva, 1996
PecR	CCCTGA	PecR	Niu J, et al. 2015
RhaR	CGGXXXXXXXXXXXCCG	RhaR	Pardo E, et al. 2014
Rlm1p-A	CTAWWWWTAG	Rlm1p	Jung and Levin 1999
Rlm1p-B	TAWWWWTAG	Rlm1p	Jung and Levin 1999
Rlm1p-C	CTAWWWWTAR	Rlm1p	Jung and Levin 2002
RsmA	TGACACA	RsmA	Yin WB, et al. 2012
SltA	CAGGCAT	SltA	Spielvogel, et al. 2008
STRE-A	AAGGGG	Msn2p/Msn4p	Peterbauer CK, et al. 2002
StuA	NWWCGCGWNM	StuA	Dutton, et al. 1997
Swi5	KGCTGR	AcFKH1	Schmitt EK, et al. 2004
TamA	CCCGAASTTCGG	TamA	Downes DJ, et al. 2014
Tec1p-A	CATTCC	AbaA	Gavrias V, et al. 1996
Tec1p-B	CATTCYY	AbaA	Gavrias V, et al. 1996
Tec1p-C	CATTCY	AbaA	Gavrias V, et al. 1996
TerR	TCGGHHWYHCGGH	TerR	Gressler M, et al. 2015
XlnR	GGNTAAA	XlnR	Andersen MR, et al.2008
Yap1p-A	TTASTMA	Afyap1	Qiao J, et al. 2008 and Lessing F, et al. 2007
Yap1p-B	TTACTAA	Afyap1	Qiao J, et al. 2008 and Lessing F, et al. 2007

Yap1p-C	TTAGTMAGC	Afyap1	Qiao J, et al. 2008 and Lessing F, et al. 2007
Yap1p-D	MTKASTMA	Afyap1	Qiao J, et al. 2008 and Lessing F, et al. 2007
Zap1p-A	ACCYYNAAGGT	ZafA	Moreno MA, et al. 2007
Zap1p-B	ACCCTMAAGGTYRT	ZafA	Moreno MA, et al. 2007
Zap1p-C	ACCCTAAAGGT	ZafA	Moreno MA, et al. 2007
